# Supplementary material for: Genetic architecture of human plasma lipidome and its link to cardiovascular disease
Source: Nat Commun. 2019 Sep 24;10:4329. doi: 10.1038/s41467-019-11954-8 (PMC6760179; doi:10.1038/s41467-019-11954-8)
Supplement: Supplementary file 1 — Supplementary Information [file 41467_2019_11954_MOESM1_ESM.pdf]

SUPPLEMENTARY INFORMATION

Genetic architecture of human plasma lipidome and its link to cardiovascular disease

Tabassum et al.

Contents

Supplementary Figures ……………………………………………. 2

Supplementary Tables …………………………………………….. 19

References ………………………………………………………. 25

Supplementary Figure 1: Genetic and phenotypic correlations among the lipid species. The upper triangle of the heatmap shows the phenotypic correlations based on plasma levels of lipid species while the lower triangle shows the genetic correlations among the lipid species. The lipid species are arranged in the order of clustering based on genetic correlations as in Figure 2.

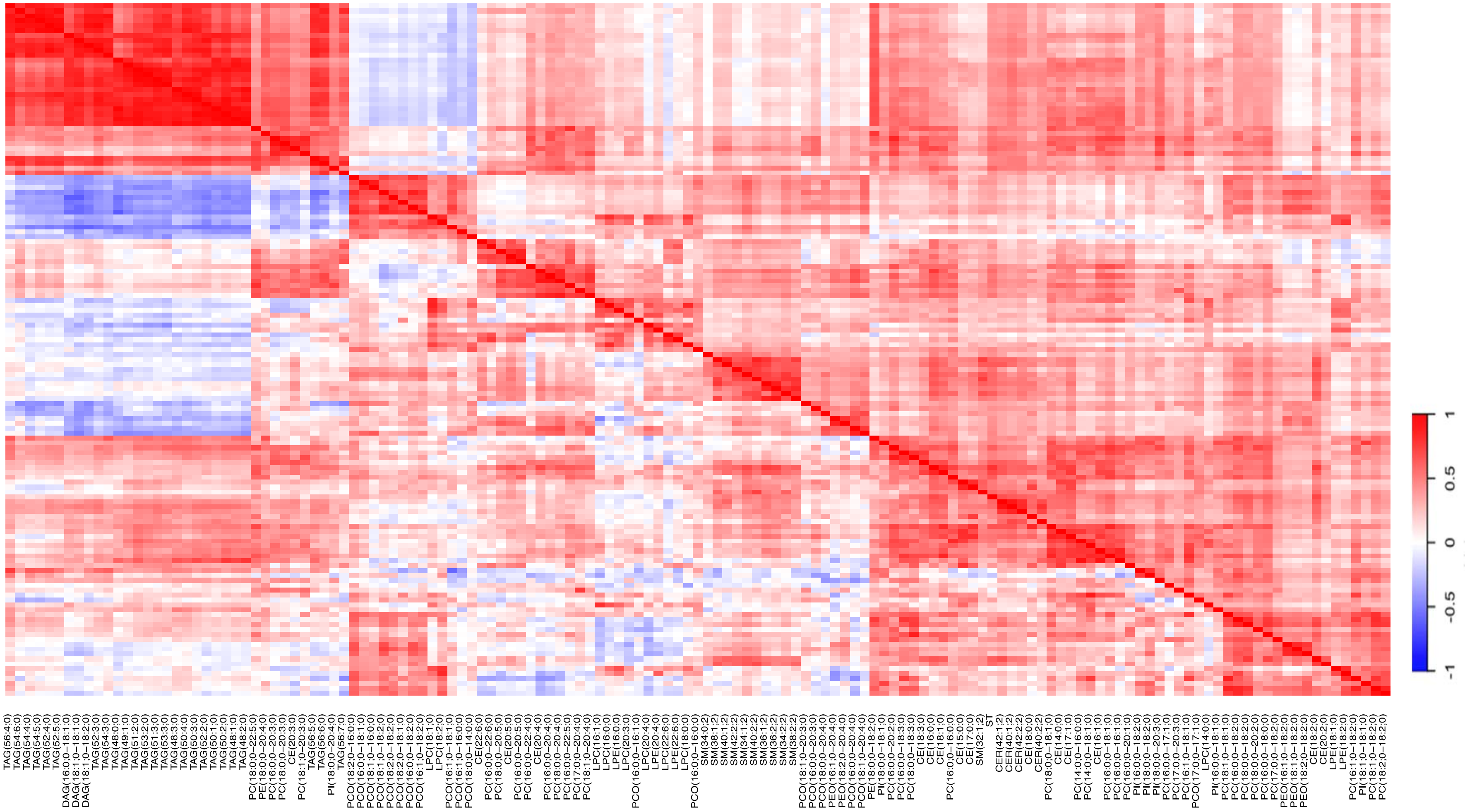

Supplementary Figure 2: Hierarchical clustering of lipid species based on phenotypic correlations between the plasma levels of lipid species. The clusters were generated using plasma levels of all lipid species. Lipids containing polyunsaturated fatty acids C20:5, C20:4, and C22:6 are highlighted with black bars.

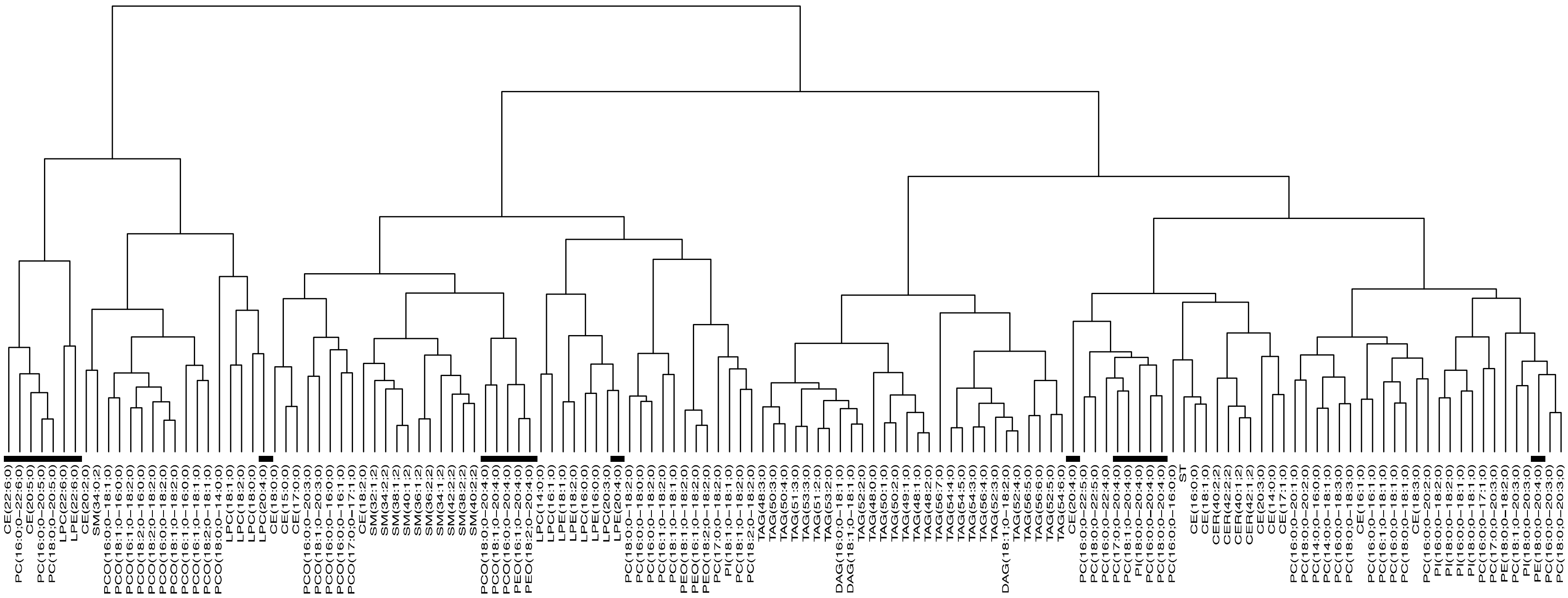

Supplementary Figure 3. Comparison of genetic correlations of traditional lipids with the molecular lipid species in the analysis adjusting for medications and in analysis after removing samples under medications. Each dot on the scatter plots represents individual molecular lipid species.

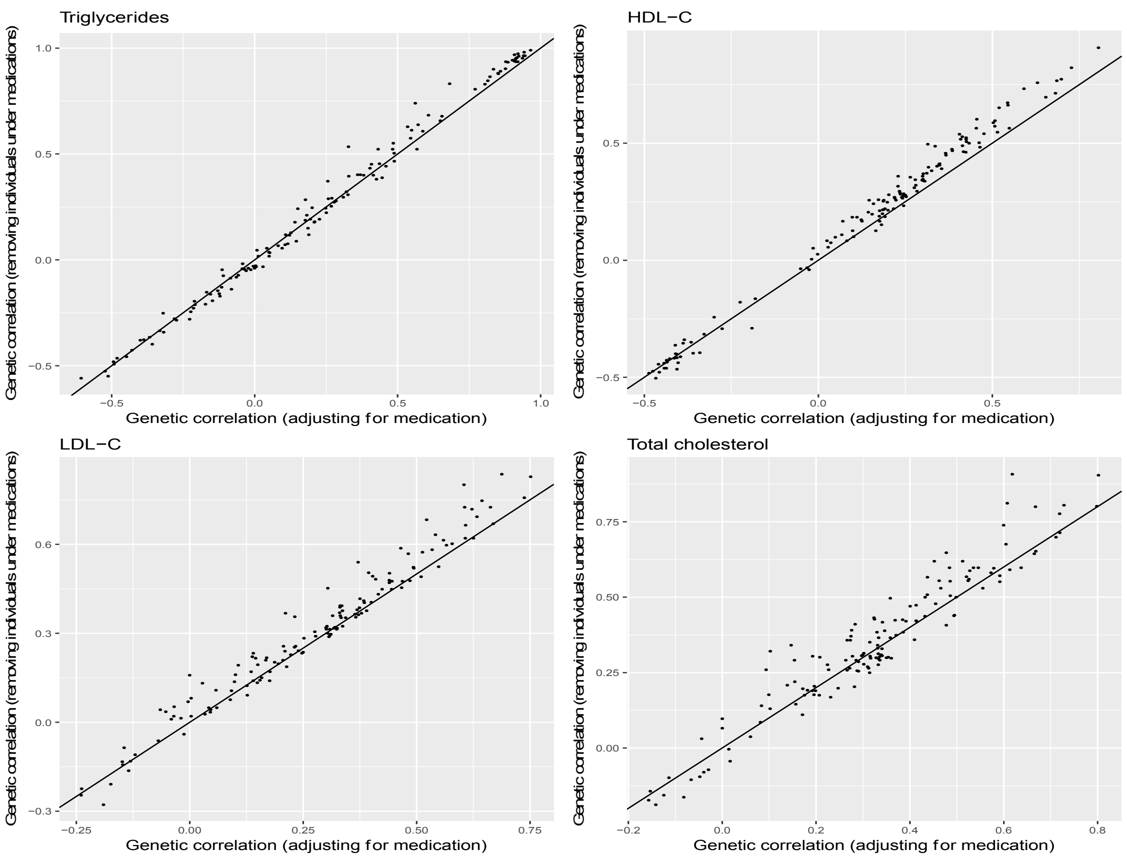

Supplementary Figure 4: Relationship between genotype and phenotype for the lead variants in 35 loci. The plasma levels of the lipid species for each genotype are plotted separately for the three batches (left: EUFAM1, middle: EUFAM2, right: FINRISK) of lipidomics data. Box plot depicts the interquartile range (IQR), median (middle line) and whiskers extending to the largest/smallest values no further than 1.5 times the IQR.

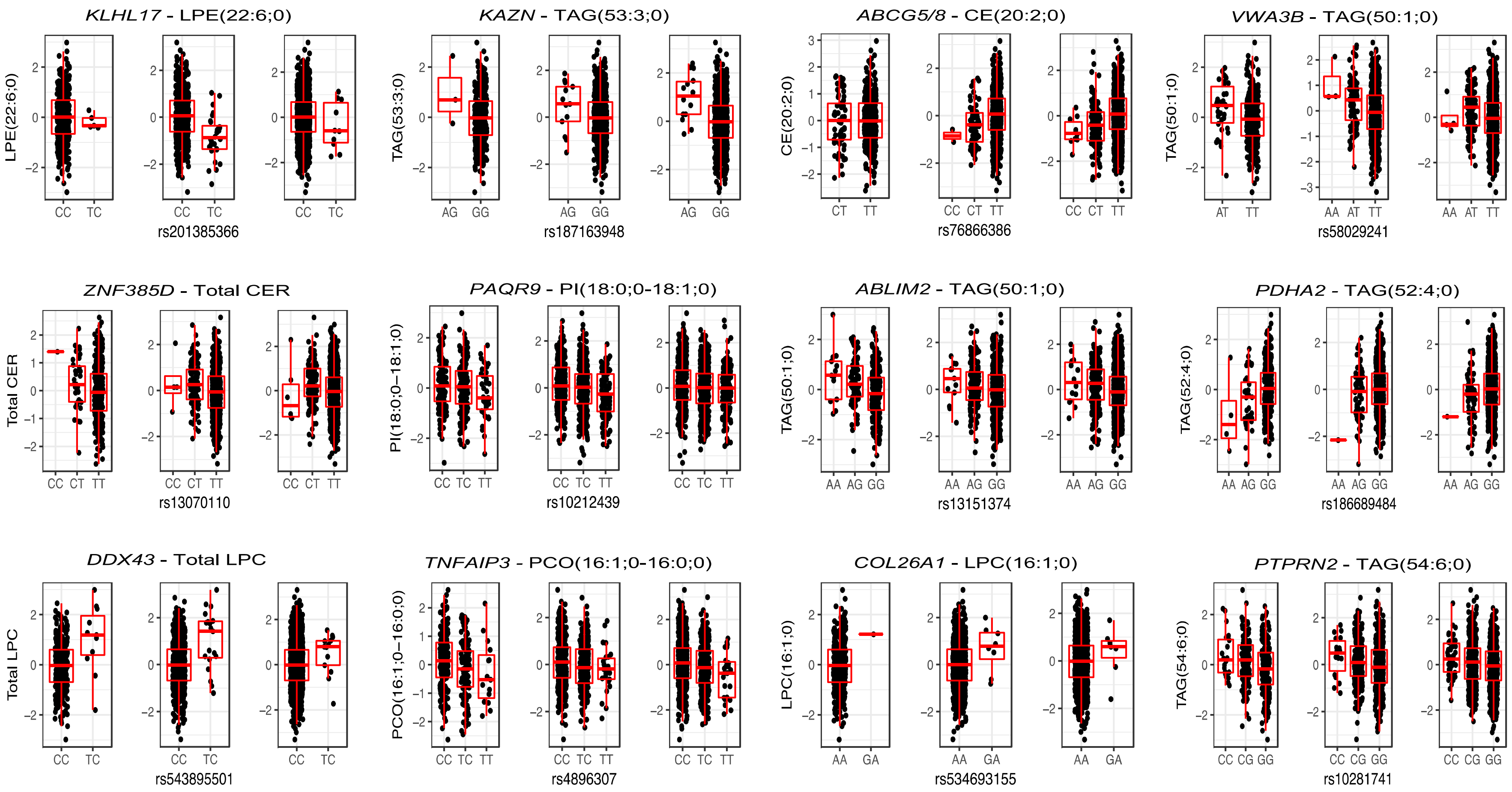

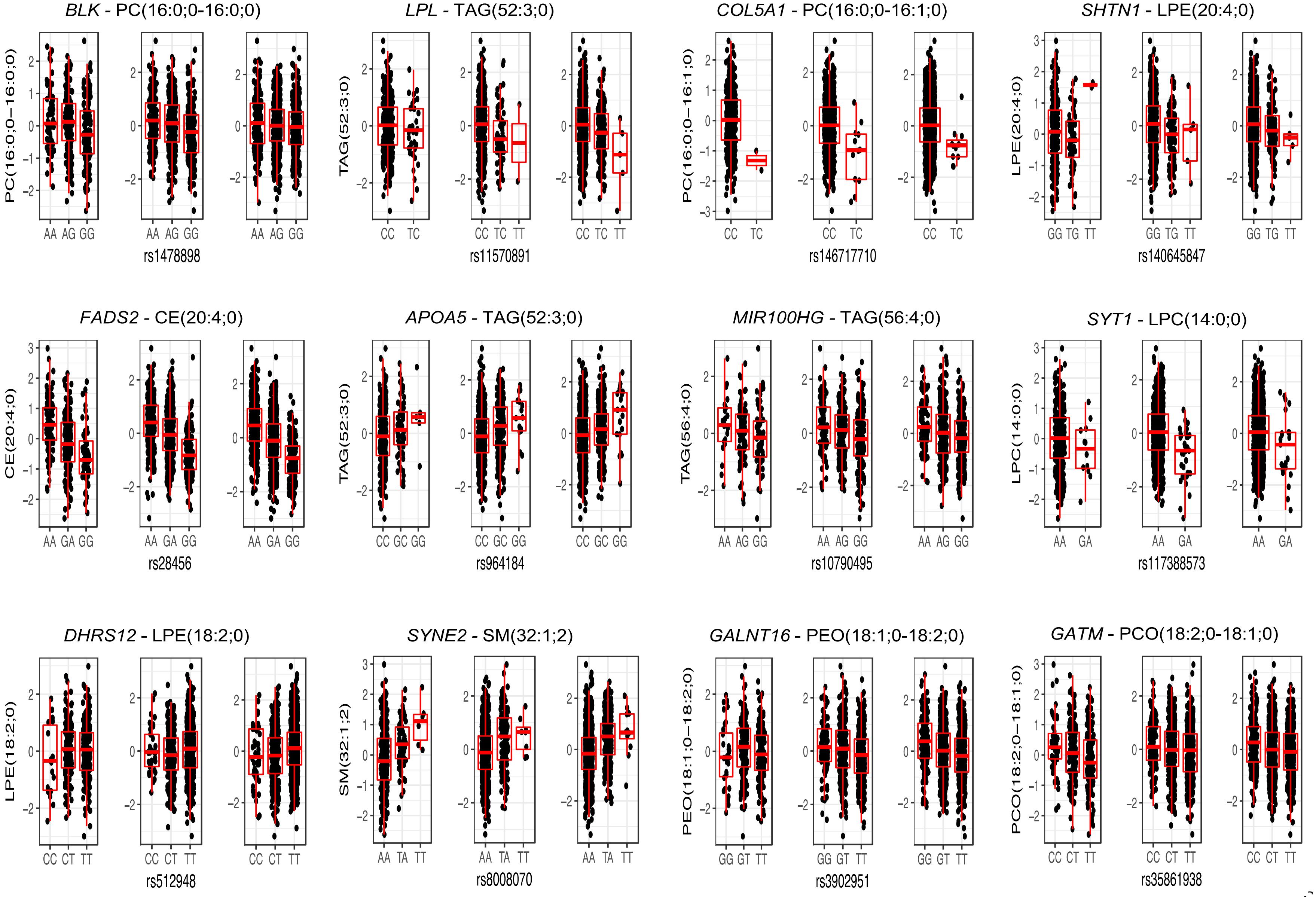
BLK - PC(16:0;0-16:0;0)
PC(16:0;0−16:0;0)
AA AG GG
rs1478898
LPL - TAG(52:3;0)
TAG(52:3;0)
CC TC
CC TC TT
rs11570891
COL5A1 - PC(16:0;0-16:1;0)
PC(16:0;0−16:1;0)
CC TC
rs146717710
SHTN1 - LPE(20:4;0)
LPE(20:4;0)
GG TG TT
rs140645847
FADS2 - CE(20:4;0)
CE(20:4;0)
AA GA GG
rs28456
APOA5 - TAG(52:3;0)
TAG(52:3;0)
CC GC GG
rs964184
MIR100HG - TAG(56:4;0)
TAG(56:4;0)
AA AG GG
rs10790495
SYT1 - LPC(14:0;0)
LPC(14:0;0)
AA GA
rs117388573
DHRS12 - LPE(18:2;0)
LPE(18:2;0)
CC CT TT
rs512948
SYNE2 - SM(32:1;2)
SM(32:1;2)
AA TA TT
rs8008070
GALNT16 - PEO(18:1;0-18:2;0)
PEO(18:1;0−18:2;0)
GG GT TT
rs3902951
GATM - PCO(18:2;0-18:1;0)
PCO(18:2;0−18:1;0)
CC CT TT
rs35861938

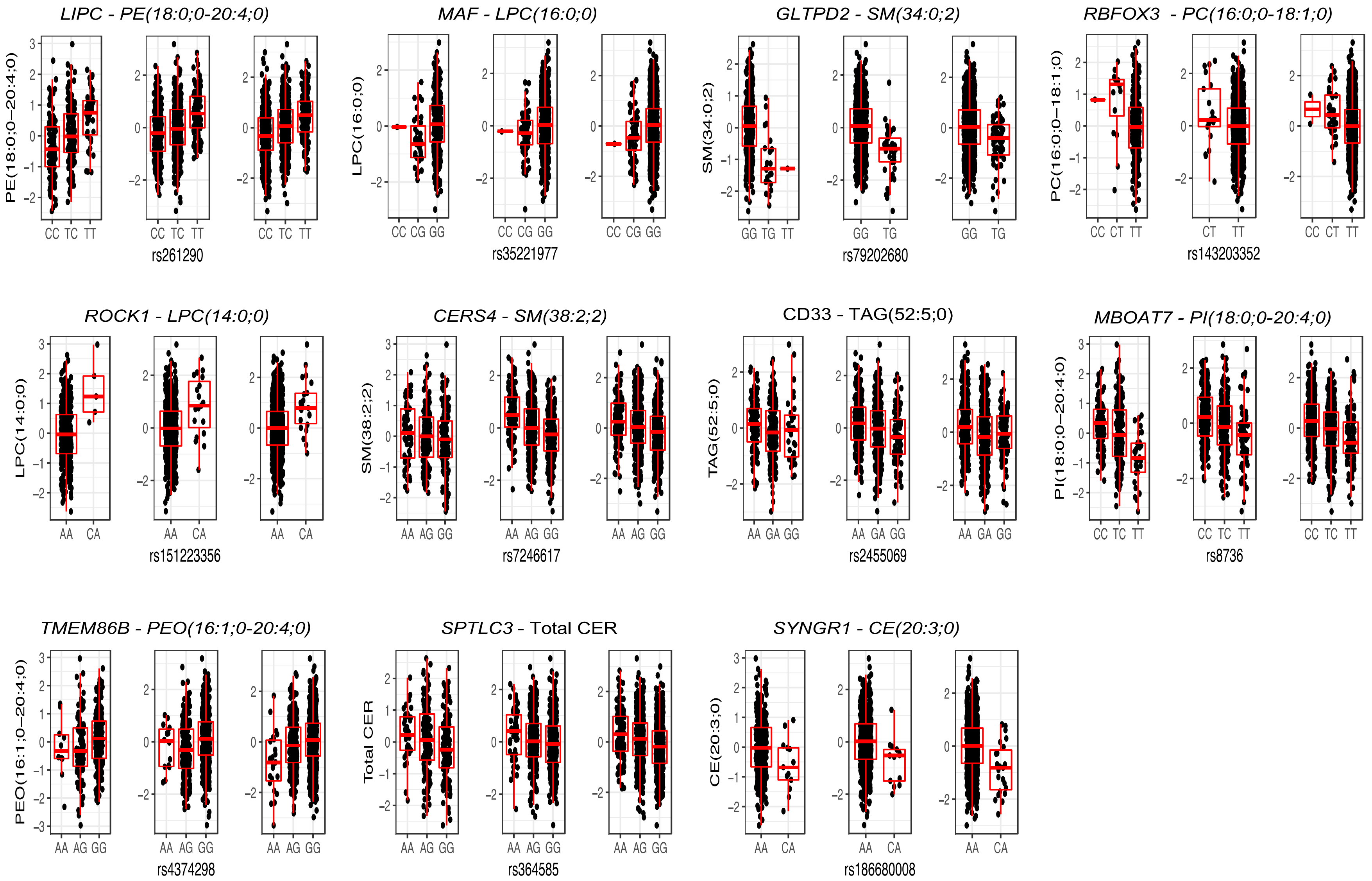
LIPC - PE(18:0;0-20:4;0)
PE(18:0;0−20:4;0)
CC TC TT
rs261290
MAF - LPC(16:0;0)
LPC(16:0;0)
CC CG GG
rs35221977
GLTPD2 - SM(34:0;2)
SM(34:0;2)
GG TG TT
GG TG
rs79202680
RBFOX3 - PC(16:0;0-18:1;0)
PC(16:0;0−18:1;0)
CC CT TT
CT TT
rs143203352
ROCK1 - LPC(14:0;0)
LPC(14:0;0)
AA CA
rs151223356
CERS4 - SM(38:2;2)
SM(38:2;2)
AA AG GG
rs7246617
CD33 - TAG(52:5;0)
TAG(52:5;0)
AA GA GG
rs2455069
MBOAT7 - PI(18:0;0-20:4;0)
PI(18:0;0−20:4;0)
CC TC TT
rs8736
TMEM86B - PEO(16:1;0-20:4;0)
PEO(16:1;0−20:4;0)
AA AG GG
rs4374298
SPTLC3 - Total CER
Total CER
AA AG GG
rs364585
SYNGR1 - CE(20:3;0)
CE(20:3;0)
AA CA
rs186680008

Supplementary Figure 5: Patterns in association of the lead variants in 35 loci with all the 141 lipid species and traditional lipids. The lipid species are grouped according to the lipid classes and arranged by increasing number of C-atoms and double bonds in each class. Association with traditional lipids levels is depicted in the far-right end. The data points are color coded by the direction of effect of alternate alleles on lipid species-decreased level (blue), increased level (red) and inconsistent direction across the cohorts (grey).

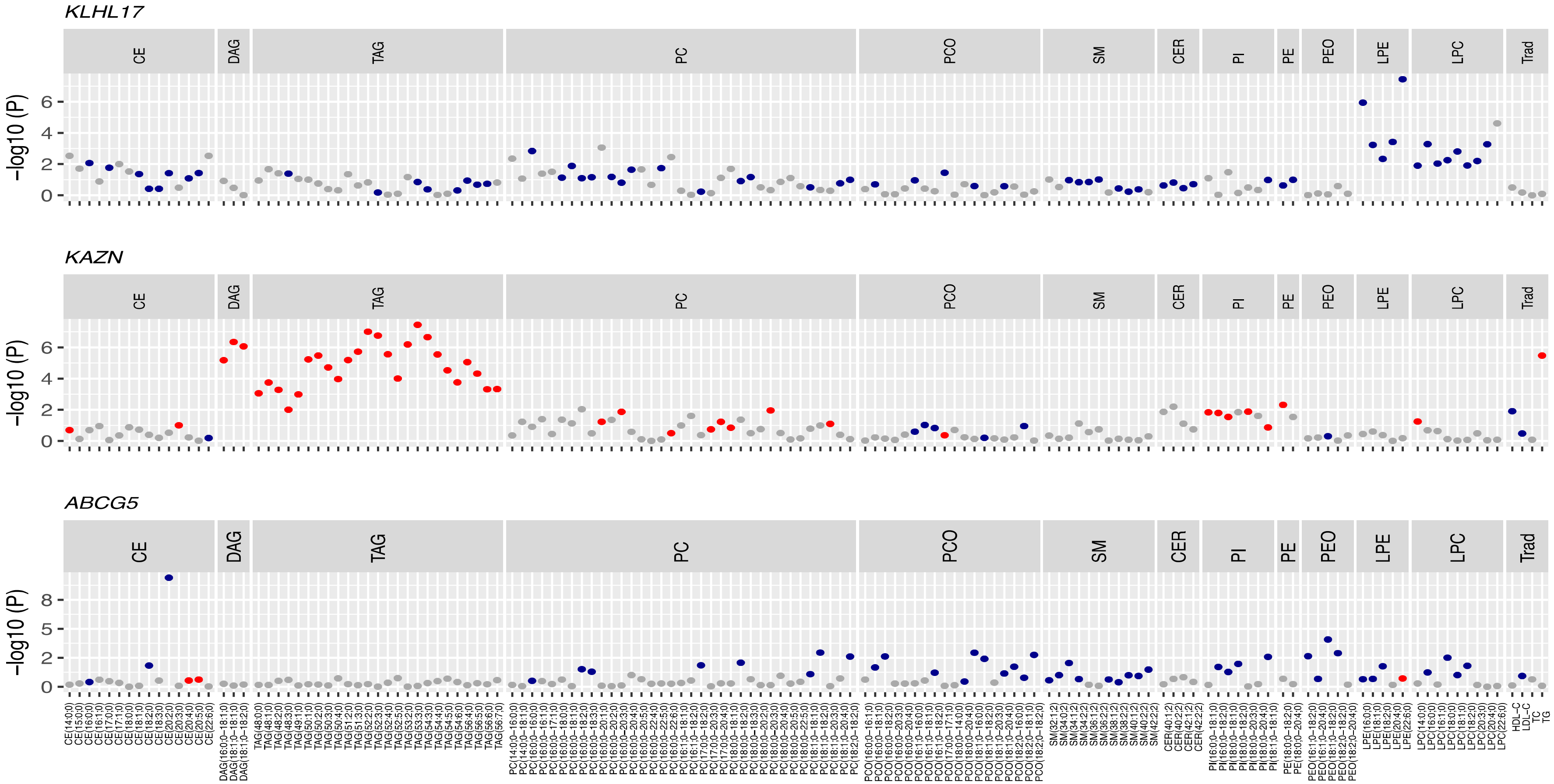

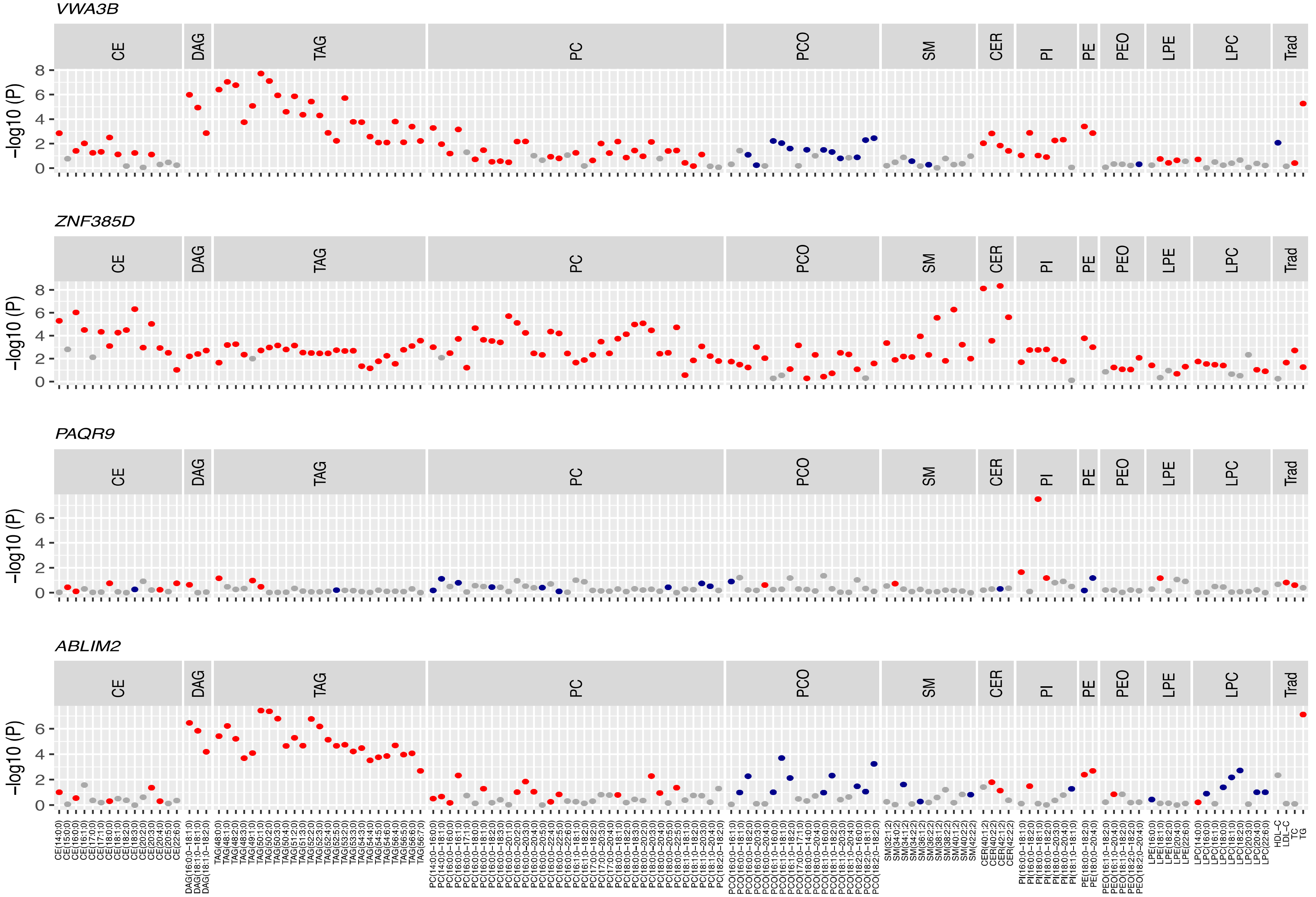
VWA3B
ZNF385D
PAQR9
ABLIM2
CE
DAG
TAG
PC
PCO
SM
CER
PI
PE
PEO
LPE
LPC
Trad
−log10 (P)

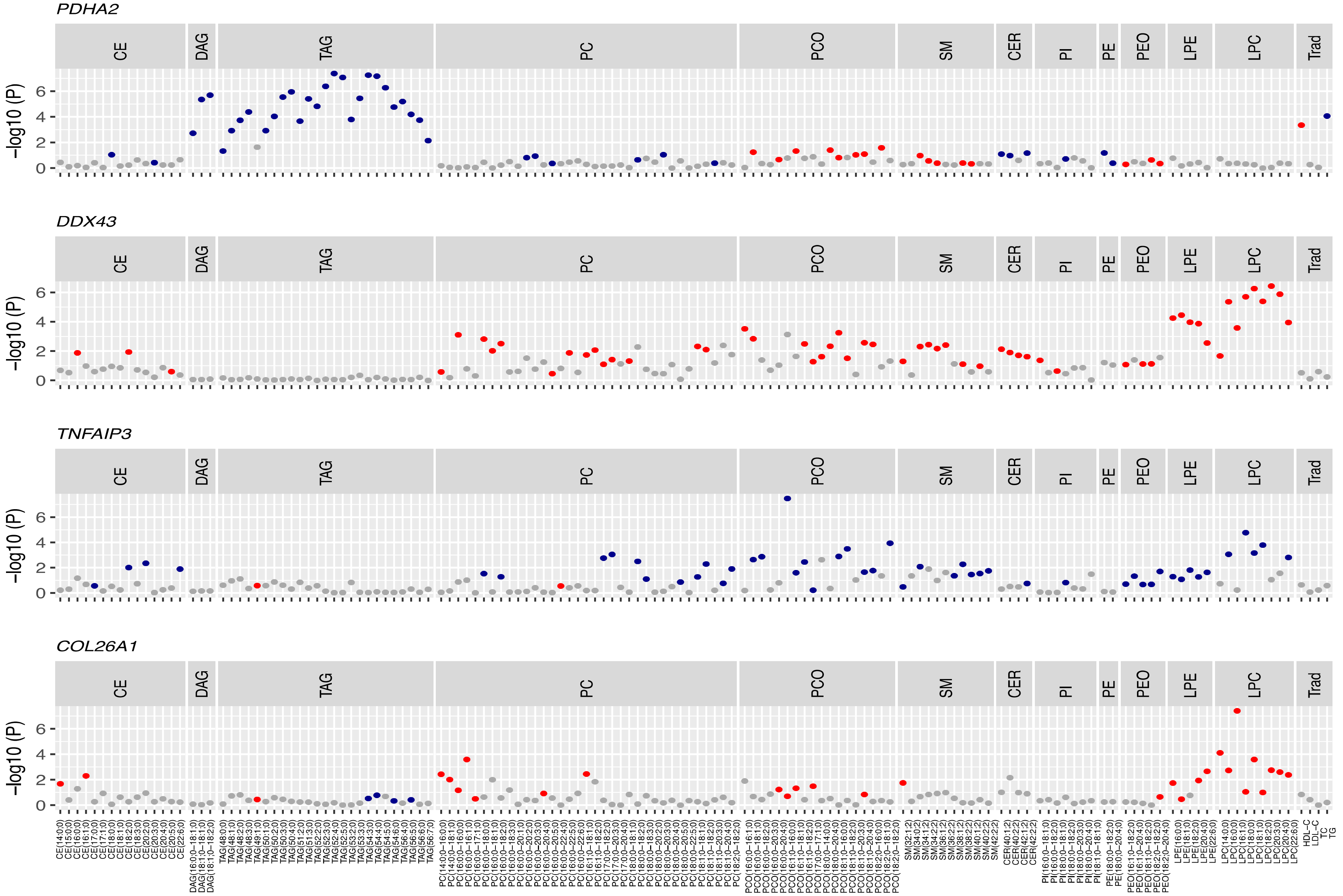
PDHA2
DDX43
TNFAIP3
COL26A1
CE
DAG
TAG
PC
PCO
SM
CER
PI
PE
PEO
LPE
LPC
Trad
-log10 (P)
0
2
4
6

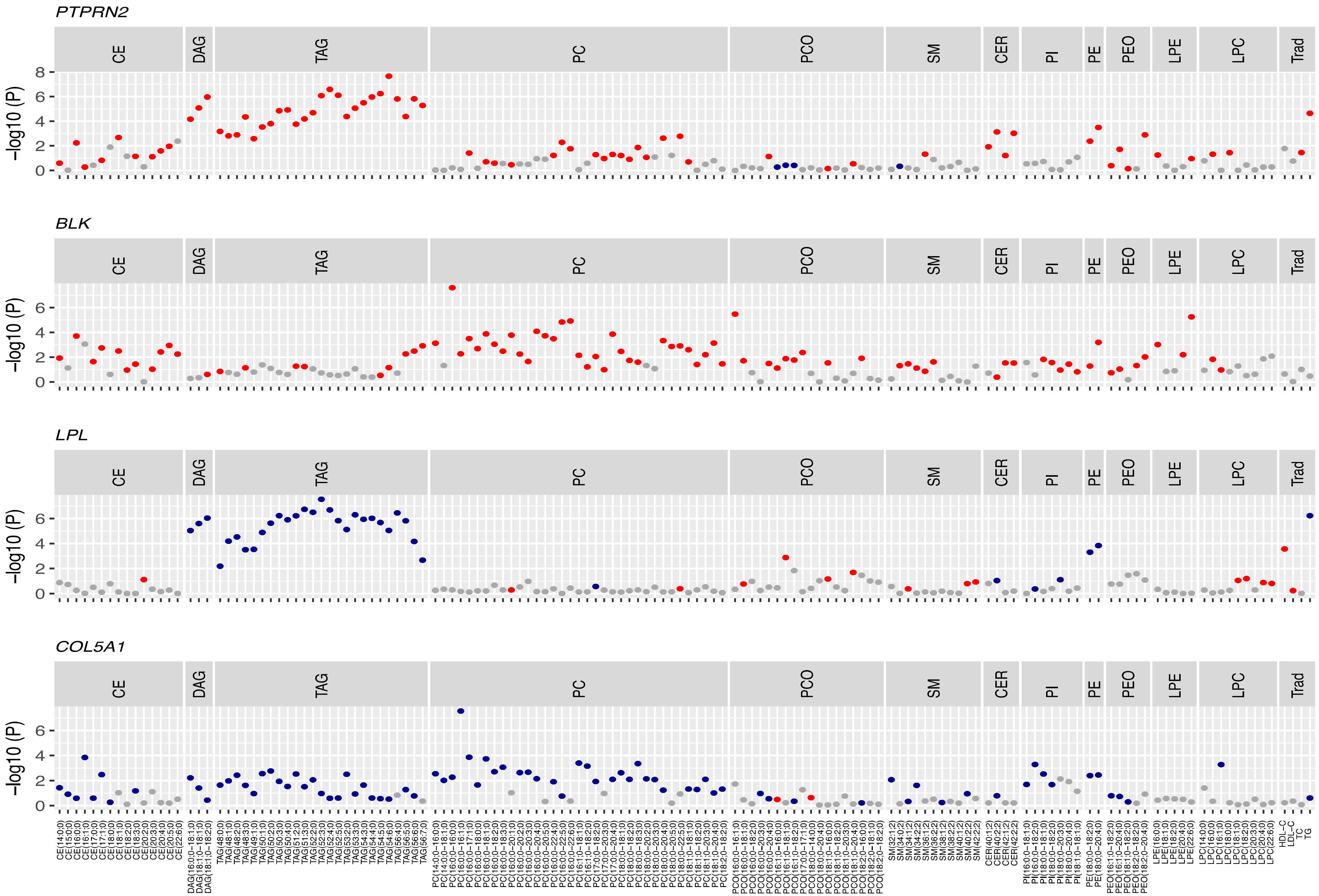
PTPRN2
BLK
LPL
COL5A1
CE
DAG
TAG
PC
PCO
SM
CER
PI
PE
PEO
LPE
LPC
Trad
−log10 (P)

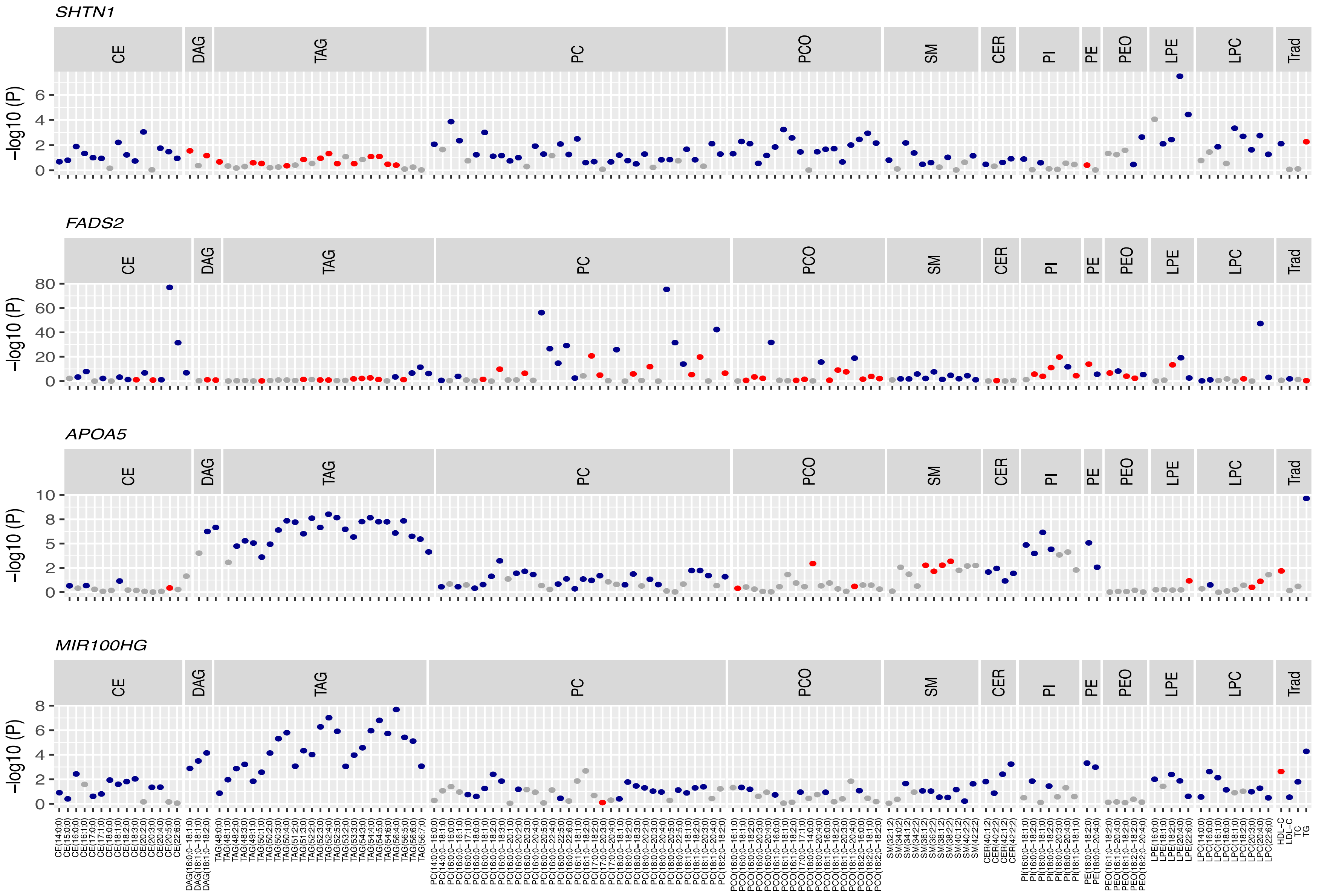

SHTN1
FADS2
APOA5
MIR100HG
CE
DAG
TAG
PC
PCO
SM
CER
PI
PE
PEO
LPE
LPC
Trad
−log10 (P)

87

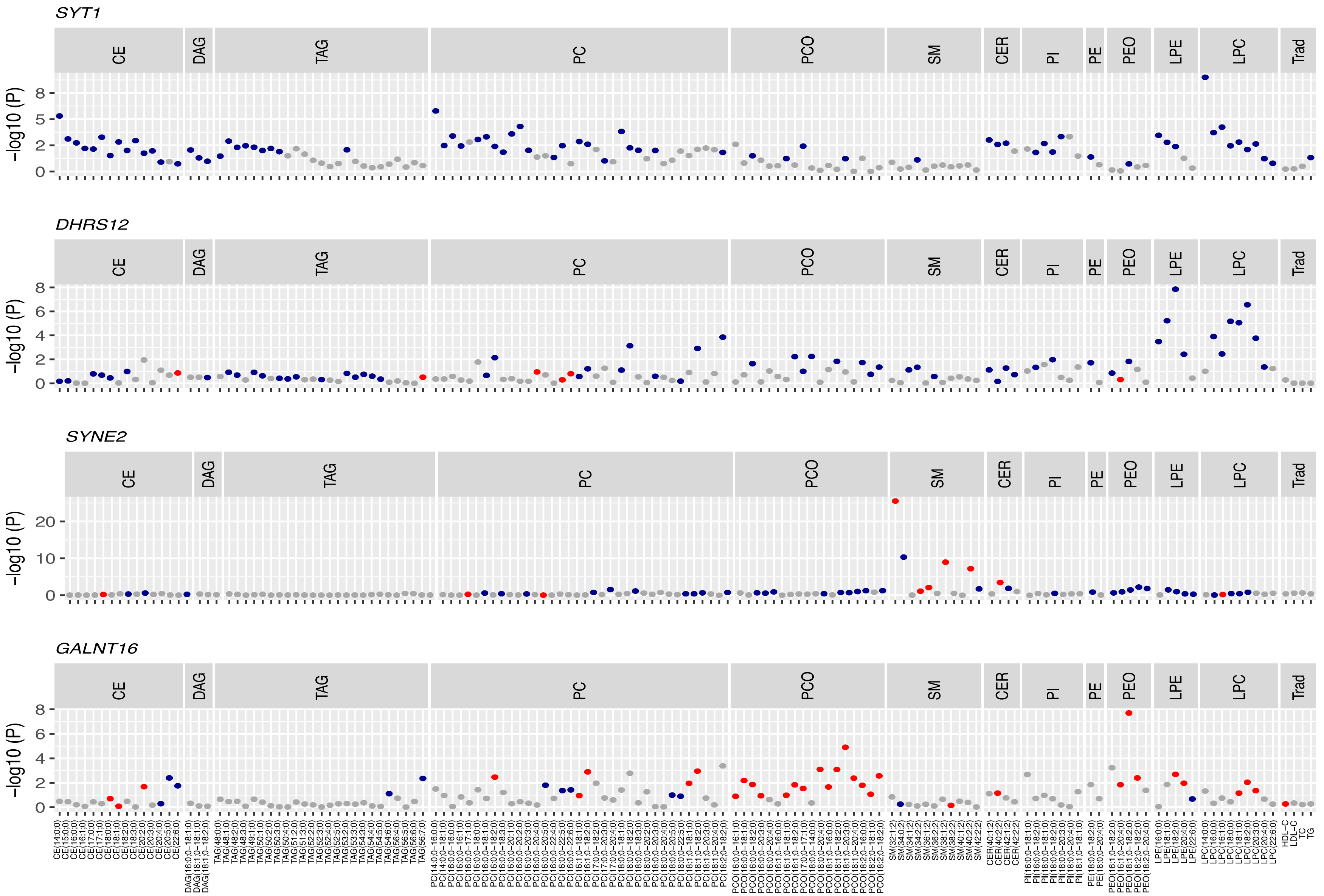
SYT1
DHRS12
SYNE2
GALNT16
CE
DAG
TAG
PC
PCO
SM
CER
PI
PE
PEO
LPE
LPC
Trad
−log10 (P)

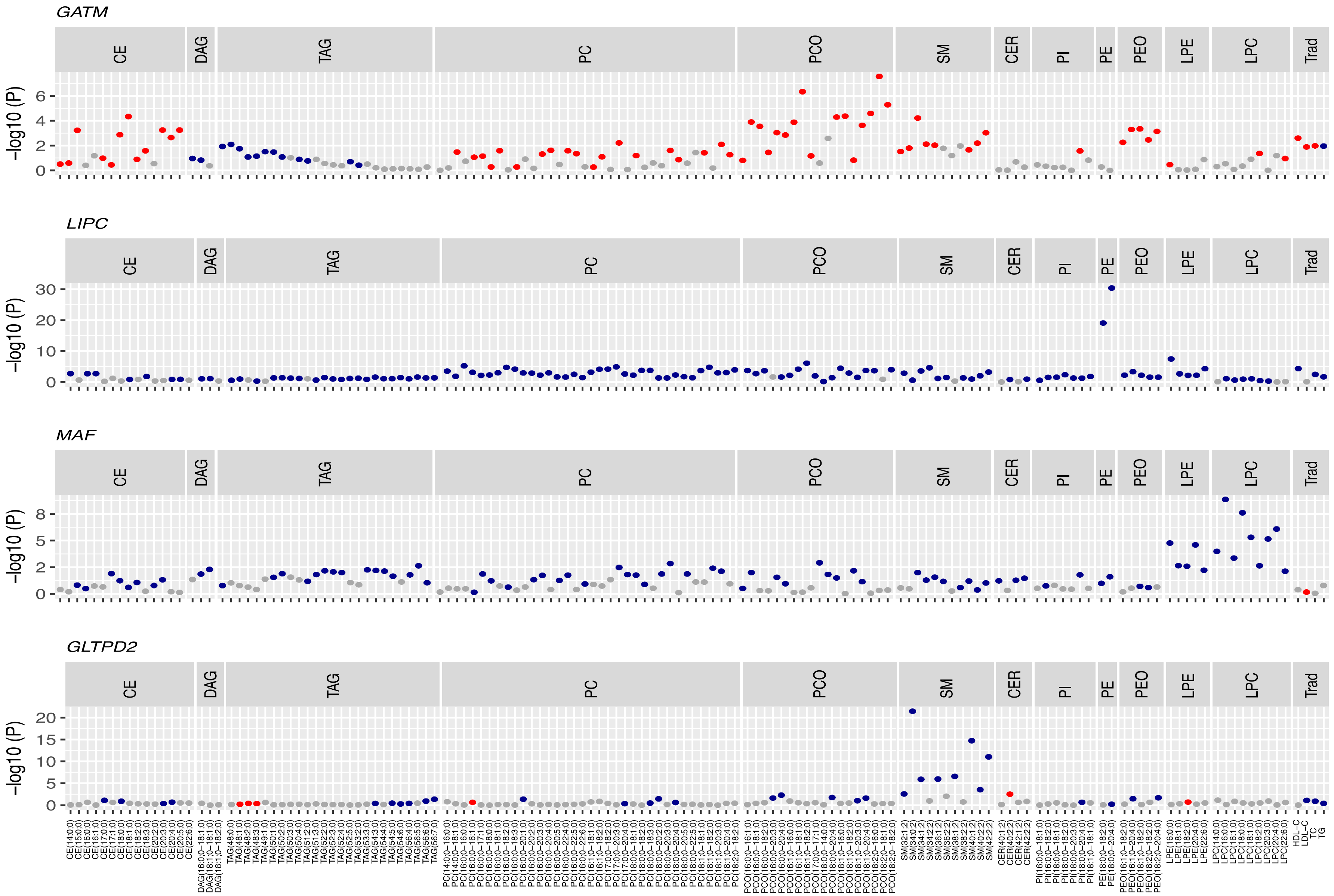
GATM
LIPC
MAF
GLTPD2
CE
DAG
TAG
PC
PCO
SM
CER
PI
PE
PEO
LPE
LPC
Trad
−log10 (P)
HDL−C
LDL−C
TC
TG

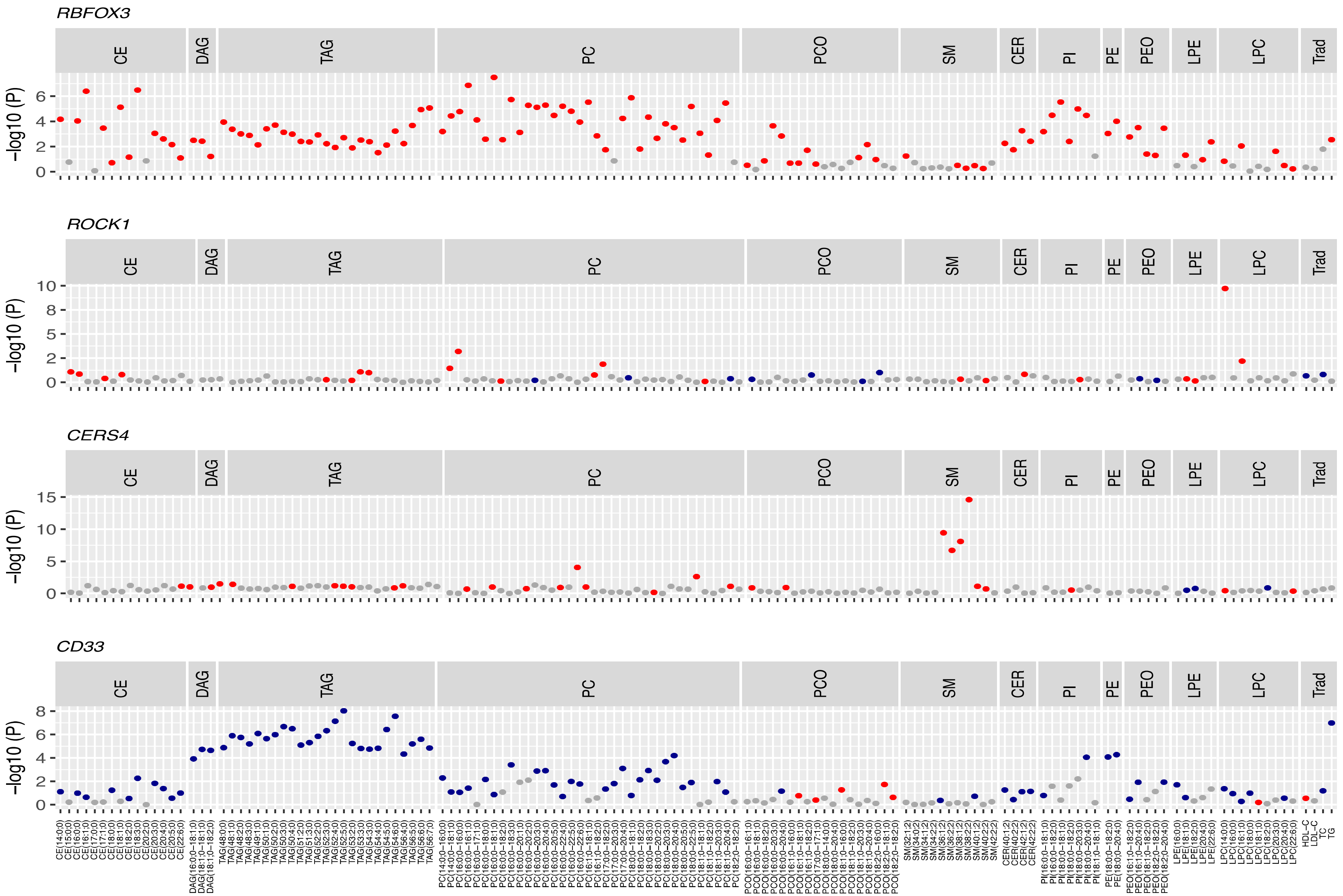
RBFOX3
ROCK1
CERS4
CD33
CE
DAG
TAG
PC
PCO
SM
CER
PI
PE
PEO
LPE
LPC
Trad
−log10 (P)
HDL−C
LDL−C
TC
TG

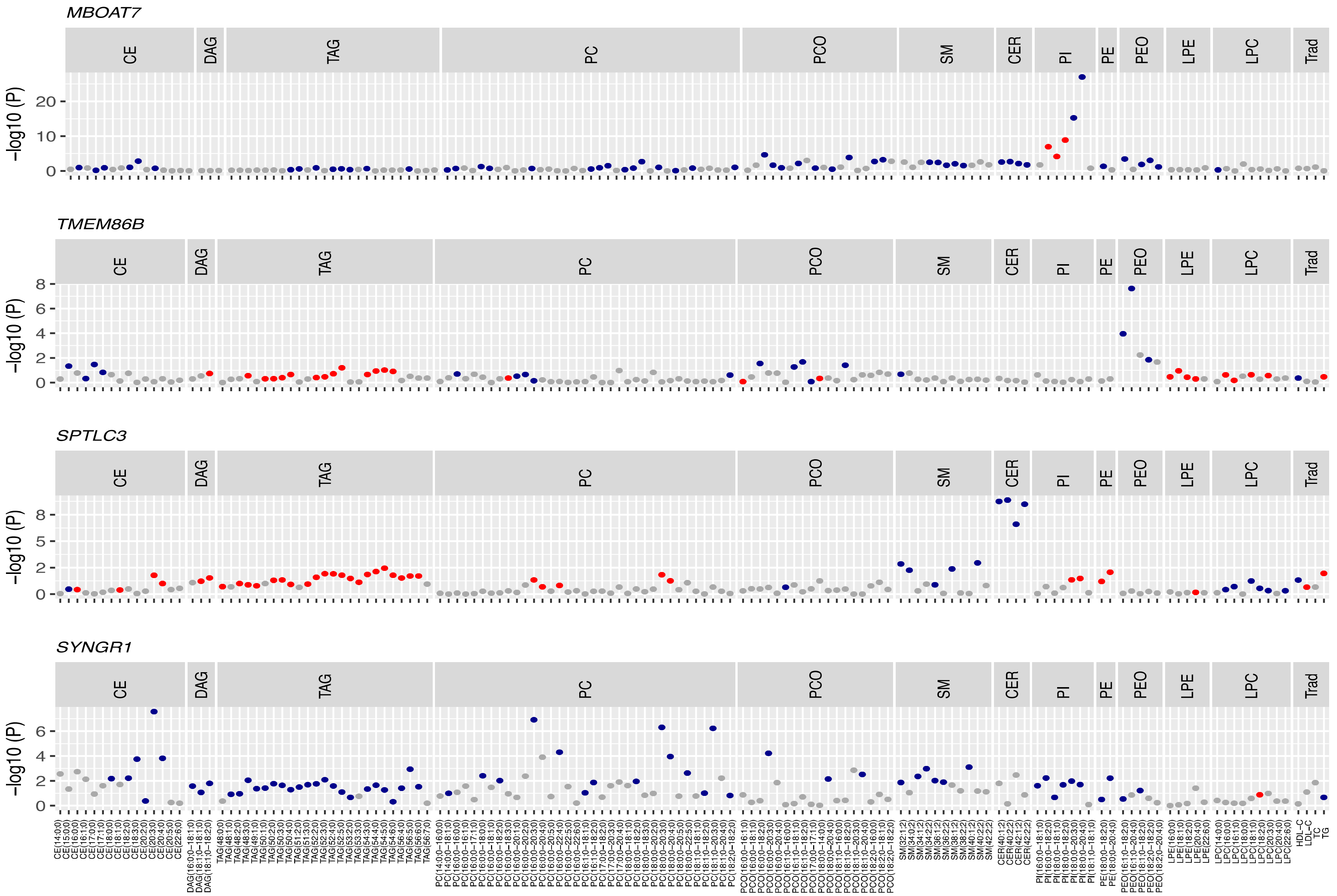
MBOATZ
TMEM86B
SPTLC3
SYNGR1
CE
DAG
TAG
PC
PCO
SM
CER
PI
PE
PEO
LPE
LPC
Trad
−log10 (P)
0
2
4
5
6
8
10
20

Supplementary Figure 6: Comparison of effect sizes of lead variants from the 35 lipid species associated genomic loci in EUFAM and FINRISK cohorts. (a) Column plot with effect sizes in two cohorts. The absolute values of the effect sizes are plotted for clarity (b) Scatter plot of the effect sizes in two cohorts.

a

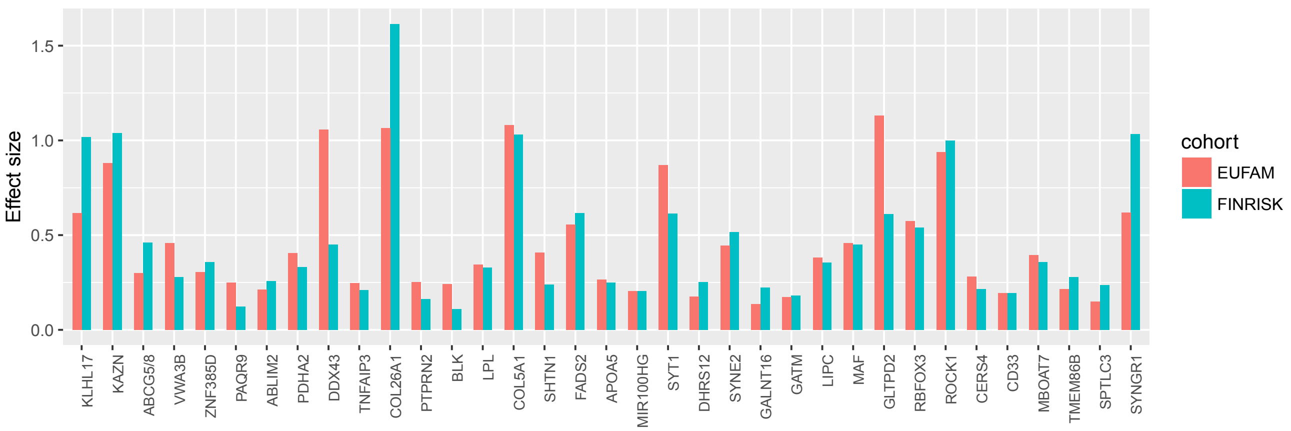

b

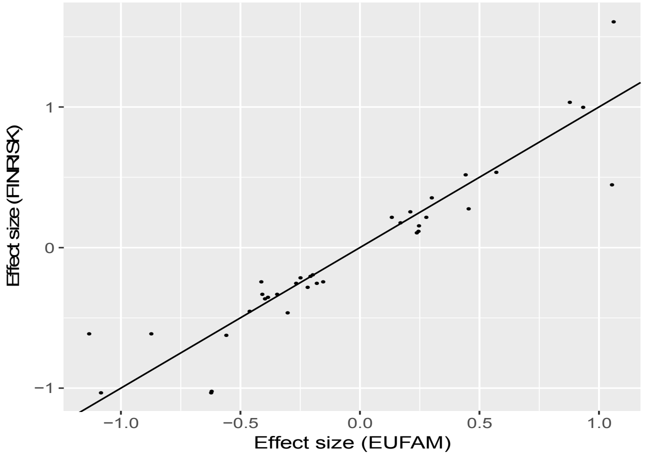

Supplementary Figure 7: Comparison of lipid species detected by different platforms. a) Comparison of all the lipid moieties identified; b) Comparison at subspecies level; c) Comparison at species level. Lipids detected by Metabolon were obtained from Long et al. 2017[1], Biocrates from Illig et al. 2013[2] and Lipotype from this study.

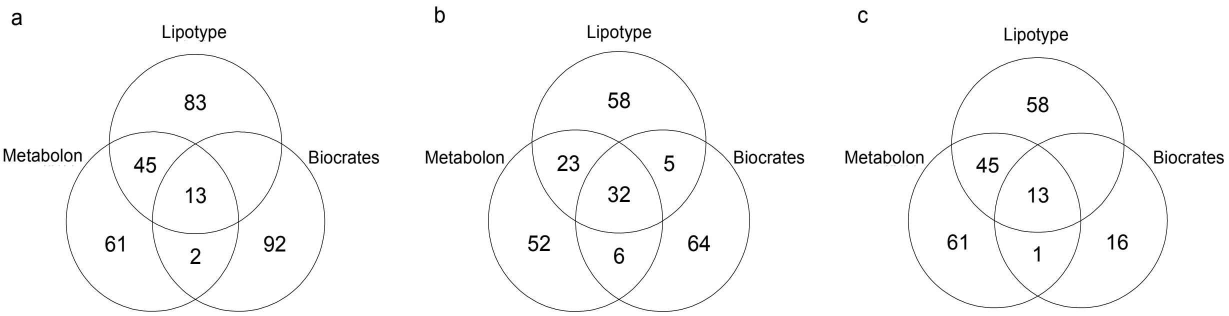

Supplementary Table 1: Basic clinical characteristics of subjects included in the study and passed the QC filtering of lipidomics data

	EUFAM	FINRISK
N	1037	1080
M/F	496/541	497/582
Age (years)	41.2 (14.4)	50.8 (14.1)
BMI (kg m^{-2})	26.2 (4.66)	26.3 (4.51)
Triglycerides (mmol l^{-1})	1.58 (1.31)	1.35 (0.91)
HDL-C (mmol l^{-1})	1.32 (0.40)	1.49 (0.38)
LDL-C (mmol l^{-1})	3.37 (1.04)	3.18 (0.95)
Total cholesterol (mmol l^{-1})	5.23 (1.59)	5.29 (1.10)

Supplementary Table 2: Heritability estimates of lipid species and phenotypic variance explained by previously known variants for traditional lipids

SN	Species	Swiss Lipids Name	Number of carbon atoms	Heritability estimates	SE	Variance explained by known lipid variants	SE
Cholesteryl Esters (CE)							
1	CE(14:0;0)	Sterol ester (27:1/14:0)	14	0.253	0.061	0.084	0.020
2	CE(15:0;0)	Sterol ester (27:1/15:0)	15	0.349	0.058	0.088	0.022
3	CE(16:0;0)	Sterol ester (27:1/16:0)	16	0.245	0.061	0.125	0.022
4	CE(16:1;0)	Sterol ester (27:1/16:1)	16	0.212	0.061	0.084	0.021
5	CE(17:0;0)	Sterol ester (27:1/17:0)	17	0.327	0.055	0.073	0.020
6	CE(17:1;0)	Sterol ester (27:1/17:1)	17	0.344	0.058	0.098	0.022
7	CE(18:0;0)	Sterol ester (27:1/18:0)	18	0.381	0.060	0.092	0.020
8	CE(18:1;0)	Sterol ester (27:1/18:1)	18	0.241	0.059	0.077	0.020
9	CE(18:2;0)	Sterol ester (27:1/18:2)	18	0.264	0.060	0.112	0.021
10	CE(18:3;0)	Sterol ester (27:1/18:3)	18	0.294	0.062	0.112	0.020
11	CE(20:2;0)	Sterol ester (27:1/20:2)	20	0.291	0.066	0.109	0.021
12	CE(20:3;0)	Sterol ester (27:1/20:3)	20	0.265	0.067	0.096	0.021
13	CE(20:4;0)	Sterol ester (27:1/20:4)	20	0.461	0.060	0.218	0.020
14	CE(20:5;0)	Sterol ester (27:1/20:5)	20	0.364	0.062	0.153	0.021
15	CE(22:6;0)	Sterol ester (27:1/22:6)	22	0.412	0.064	0.088	0.021
Diacylglyerides (DAG)							
16	DAG(16:0;0-18:1;0)	Diacylglycerol (16:0_18:1)	34	0.308	0.070	0.128	0.022
17	DAG(18:1;0-18:1;0)	Diacylglycerol (18:1_18:1)	36	0.245	0.071	0.114	0.019
18	DAG(18:1;0-18:2;0)	Diacylglycerol (18:1_18:2)	36	0.252	0.072	0.132	0.021
Triacylglycerides (TAG)							
19	TAG(48:0;0)	Triacylglycerol (48:0)	48	0.299	0.066	0.083	0.022
20	TAG(48:1;0)	Triacylglycerol (48:1)	48	0.305	0.066	0.100	0.021
21	TAG(48:2;0)	Triacylglycerol (48:2)	48	0.293	0.067	0.090	0.020
22	TAG(48:3;0)	Triacylglycerol (48:3)	48	0.237	0.068	0.081	0.022
23	TAG(49:1;0)	Triacylglycerol (49:1)	49	0.310	0.066	0.092	0.022
24	TAG(50:1;0)	Triacylglycerol (50:1)	50	0.294	0.069	0.115	0.021
25	TAG(50:2;0)	Triacylglycerol (50:2)	50	0.253	0.068	0.111	0.020
26	TAG(50:3;0)	Triacylglycerol (50:3)	50	0.232	0.068	0.118	0.021
27	TAG(50:4;0)	Triacylglycerol (50:4)	50	0.219	0.069	0.115	0.022
28	TAG(51:2;0)	Triacylglycerol (51:2)	51	0.318	0.067	0.114	0.020
29	TAG(51:3;0)	Triacylglycerol (51:3)	51	0.252	0.066	0.127	0.021
30	TAG(52:2;0)	Triacylglycerol (52:2)	52	0.288	0.070	0.122	0.020
31	TAG(52:3;0)	Triacylglycerol (52:3)	52	0.247	0.070	0.144	0.021
32	TAG(52:4;0)	Triacylglycerol (52:4)	52	0.228	0.069	0.143	0.021
33	TAG(52:5;0)	Triacylglycerol (52:5)	52	0.238	0.071	0.135	0.021
34	TAG(53:2;0)	Triacylglycerol (53:2)	53	0.366	0.067	0.131	0.022
35	TAG(53:3;0)	Triacylglycerol (53:3)	53	0.262	0.067	0.133	0.022
36	TAG(54:3;0)	Triacylglycerol (54:3)	54	0.240	0.069	0.112	0.020
37	TAG(54:4;0)	Triacylglycerol (54:4)	54	0.203	0.067	0.119	0.021
38	TAG(54:5;0)	Triacylglycerol (54:5)	54	0.229	0.069	0.125	0.021

39	TAG(54:6;0)	Triacylglycerol (54:6)	54	0.291	0.074	0.129	0.021
40	TAG(56:4;0)	Triacylglycerol (56:4)	56	0.308	0.076	0.113	0.022
41	TAG(56:5;0)	Triacylglycerol (56:5)	56	0.322	0.074	0.149	0.021
42	TAG(56:6;0)	Triacylglycerol (56:6)	56	0.263	0.069	0.156	0.021
43	TAG(56:7;0)	Triacylglycerol (56:7)	56	0.337	0.070	0.117	0.021
Phosphatidylcholines (PC)							
44	PC(14:0;0-16:0;0)	Phosphatidylcholine (14:0_16:0)	30	0.227	0.058	0.062	0.019
45	PC(14:0;0-18:1;0)	Phosphatidylcholine (14:0_18:1)	32	0.328	0.060	0.089	0.022
46	PC(16:0;0-16:0;0)	Phosphatidylcholine (16:0_16:0)	32	0.132	0.057	0.104	0.022
47	PC(16:0;0-16:1;0)	Phosphatidylcholine (16:0_16:1)	32	0.116	0.057	0.056	0.018
48	PC(16:0;0-17:1;0)	Phosphatidylcholine (16:0_17:1)	33	0.284	0.062	0.072	0.022
49	PC(16:0;0-18:0;0)	Phosphatidylcholine (16:0_18:0)	34	0.193	0.060	0.078	0.020
50	PC(16:0;0-18:1;0)	Phosphatidylcholine (16:0_18:1)	34	0.160	0.058	0.090	0.022
51	PC(16:0;0-18:2;0)	Phosphatidylcholine (16:0_18:2)	34	0.221	0.063	0.143	0.021
52	PC(16:0;0-18:3;0)	Phosphatidylcholine (16:0_18:3)	34	0.204	0.064	0.109	0.022
53	PC(16:0;0-20:1;0)	Phosphatidylcholine (16:0_20:1)	36	0.166	0.056	0.048	0.019
54	PC(16:0;0-20:2;0)	Phosphatidylcholine (16:0_20:2)	36	0.163	0.064	0.083	0.020
55	PC(16:0;0-20:3;0)	Phosphatidylcholine (16:0_20:3)	36	0.142	0.065	0.070	0.019
56	PC(16:0;0-20:4;0)	Phosphatidylcholine (16:0_20:4)	36	0.352	0.063	0.176	0.019
57	PC(16:0;0-20:5;0)	Phosphatidylcholine (16:0_20:5)	36	0.321	0.063	0.124	0.020
58	PC(16:0;0-22:4;0)	Phosphatidylcholine (16:0_22:4)	38	0.184	0.063	0.078	0.019
59	PC(16:0;0-22:5;0)	Phosphatidylcholine (16:0_22:5)	38	0.351	0.065	0.156	0.021
60	PC(16:0;0-22:6;0)	Phosphatidylcholine (16:0_22:6)	38	0.323	0.066	0.061	0.020
61	PC(16:1;0-18:1;0)	Phosphatidylcholine (16:1_18:1)	34	0.172	0.057	0.094	0.020
62	PC(16:1;0-18:2;0)	Phosphatidylcholine (16:1_18:2)	34	0.181	0.056	0.129	0.020
63	PC(17:0;0-18:2;0)	Phosphatidylcholine (17:0_18:2)	35	0.334	0.061	0.105	0.020
64	PC(17:0;0-20:3;0)	Phosphatidylcholine (17:0_20:3)	37	0.419	0.071	0.101	0.023
65	PC(17:0;0-20:4;0)	Phosphatidylcholine (17:0_20:4)	37	0.539	0.062	0.137	0.020
66	PC(18:0;0-18:1;0)	Phosphatidylcholine (18:0_18:1)	36	0.232	0.057	0.096	0.022
67	PC(18:0;0-18:2;0)	Phosphatidylcholine (18:0_18:2)	36	0.334	0.063	0.132	0.021
68	PC(18:0;0-18:3;0)	Phosphatidylcholine (18:0_18:3)	36	0.244	0.064	0.080	0.021
69	PC(18:0;0-20:2;0)	Phosphatidylcholine (18:0_20:2)	38	0.348	0.064	0.108	0.020
70	PC(18:0;0-20:3;0)	Phosphatidylcholine (18:0_20:3)	38	0.261	0.067	0.078	0.020
71	PC(18:0;0-20:4;0)	Phosphatidylcholine (18:0_20:4)	38	0.471	0.061	0.206	0.019
72	PC(18:0;0-20:5;0)	Phosphatidylcholine (18:0_20:5)	38	0.297	0.064	0.134	0.021
73	PC(18:0;0-22:5;0)	Phosphatidylcholine (18:0_22:5)	40	0.299	0.066	0.105	0.020
74	PC(18:1;0-18:1;0)	Phosphatidylcholine (18:1_18:1)	36	0.272	0.058	0.110	0.020
75	PC(18:1;0-18:2;0)	Phosphatidylcholine (18:1_18:2)	36	0.312	0.061	0.156	0.021
76	PC(18:1;0-20:3;0)	Phosphatidylcholine (18:1_20:3)	38	0.175	0.059	0.067	0.019
77	PC(18:1;0-20:4;0)	Phosphatidylcholine (18:1_20:4)	38	0.367	0.064	0.162	0.020
78	PC(18:2;0-18:2;0)	Phosphatidylcholine (18:2_18:2)	36	0.257	0.061	0.097	0.020
Phosphatidylcholine-ethers (PCO)							
79	PCO(16:0;0-16:0;0)	Phosphatidylcholine (O-16:0_16:0)	32	0.281	0.063	0.053	0.021
80	PCO(16:0;0-16:1;0)	Phosphatidylcholine (O-16:0_16:1)	32	0.205	0.067	0.049	0.018
81	PCO(16:0;0-18:1;0)	Phosphatidylcholine (O-16:0_18:1)	34	0.254	0.063	0.089	0.021
82	PCO(16:0;0-18:2;0)	Phosphatidylcholine (O-16:0_18:2)	34	0.287	0.061	0.095	0.021
83	PCO(16:0;0-20:3;0)	Phosphatidylcholine (O-16:0_20:3)	36	0.145	0.057	0.058	0.018

84	PCO(16:0;0-20:4;0)	Phosphatidylcholine (O-16:0_20:4)	36	0.309	0.062	0.134	0.020
85	PCO(16:1;0-16:0;0)	Phosphatidylcholine (O-16:1_16:0)	32	0.319	0.062	0.052	0.019
86	PCO(16:1;0-18:1;0)	Phosphatidylcholine (O-16:1_18:1)	34	0.361	0.062	0.111	0.022
87	PCO(16:1;0-18:2;0)	Phosphatidylcholine (O-16:1_18:2)	34	0.323	0.064	0.094	0.020
88	PCO(17:0;0-17:1;0)	Phosphatidylcholine (O-17:0_17:1)	34	0.296	0.058	0.079	0.021
89	PCO(18:0;0-14:0;0)	Phosphatidylcholine (O-18:0_14:0)	32	0.382	0.065	0.067	0.021
90	PCO(18:0;0-20:4;0)	Phosphatidylcholine (O-18:0_20:4)	38	0.223	0.063	0.079	0.018
91	PCO(18:1;0-16:0;0)	Phosphatidylcholine (O-18:1_16:0)	34	0.332	0.062	0.107	0.021
92	PCO(18:1;0-18:2;0)	Phosphatidylcholine (O-18:1_18:2)	36	0.306	0.064	0.093	0.019
93	PCO(18:1;0-20:3;0)	Phosphatidylcholine (O-18:1_20:3)	38	0.233	0.063	0.089	0.021
94	PCO(18:1;0-20:4;0)	Phosphatidylcholine (O-18:1_20:4)	38	0.243	0.059	0.105	0.019
95	PCO(18:2;0-16:0;0)	Phosphatidylcholine (O-18:2_16:0)	34	0.282	0.063	0.074	0.020
96	PCO(18:2;0-18:1;0)	Phosphatidylcholine (O-18:2_18:1)	36	0.335	0.072	0.062	0.017
97	PCO(18:2;0-18:2;0)	Phosphatidylcholine (O-18:2_18:2)	36	0.305	0.062	0.080	0.019
Sphingomyelins (SM)							
98	SM(32:1;2)	Sphingomyelin (d32:1)	32	0.353	0.060	0.144	0.024
99	SM(34:0;2)	Sphingomyelin (d34:0)	34	0.411	0.061	0.150	0.024
100	SM(34:1;2)	Sphingomyelin (d34:1)	34	0.313	0.059	0.098	0.021
101	SM(34:2;2)	Sphingomyelin (d34:2)	34	0.375	0.064	0.104	0.022
102	SM(36:1;2)	Sphingomyelin (d36:1)	36	0.320	0.070	0.080	0.020
103	SM(36:2;2)	Sphingomyelin (d36:2)	36	0.410	0.069	0.081	0.021
104	SM(38:1;2)	Sphingomyelin (d38:1)	38	0.272	0.068	0.092	0.021
105	SM(38:2;2)	Sphingomyelin (d38:2)	38	0.350	0.073	0.063	0.019
106	SM(40:1;2)	Sphingomyelin (d40:1)	40	0.307	0.064	0.114	0.021
107	SM(40:2;2)	Sphingomyelin (d40:2)	40	0.241	0.063	0.104	0.021
108	SM(42:2;2)	Sphingomyelin (d42:2)	42	0.329	0.061	0.135	0.022
Ceramides (Cer)							
109	CER(40:1;2)	Ceramide (d40:1)	40	0.397	0.062	0.121	0.021
110	CER(40:2;2)	Ceramide (d40:2)	40	0.355	0.061	0.135	0.023
111	CER(42:1;2)	Ceramide (d42:1)	42	0.389	0.060	0.120	0.022
112	CER(42:2;2)	Ceramide (d42:2)	42	0.391	0.060	0.116	0.021
Phosphatidylinositols (PI)							
113	PI(16:0;0-18:1;0)	Phosphatidylinositol (16:0_18:1)	34	0.222	0.064	0.079	0.022
114	PI(16:0;0-18:2;0)	Phosphatidylinositol (16:0_18:2)	34	0.106	0.062	0.120	0.022
115	PI(18:0;0-18:1;0)	Phosphatidylinositol (18:0_18:1)	36	0.197	0.061	0.139	0.023
116	PI(18:0;0-18:2;0)	Phosphatidylinositol (18:0_18:2)	36	0.167	0.063	0.141	0.022
117	PI(18:0;0-20:3;0)	Phosphatidylinositol (18:0_20:3)	38	0.197	0.059	0.104	0.018
118	PI(18:0;0-20:4;0)	Phosphatidylinositol (18:0_20:4)	38	0.308	0.065	0.115	0.019
119	PI(18:1;0-18:1;0)	Phosphatidylinositol (18:1_18:1)	36	0.179	0.063	0.089	0.020
Phosphatidylethanolamines (PE)							
120	PE(18:0;0-18:2;0)	Phosphatidylethanolamine (18:0_18:2)	36	0.333	0.072	0.196	0.022
121	PE(18:0;0-20:4;0)	Phosphatidylethanolamine (18:0_20:4)	38	0.362	0.067	0.203	0.021
Phosphatidylethanolamine-ethers (PEO)							
122	PEO(16:1;0-18:2;0)	Phosphatidylethanolamine (O-16:1_18:2)	34	0.344	0.064	0.087	0.020
123	PEO(16:1;0-20:4;0)	Phosphatidylethanolamine (O-16:1_20:4)	36	0.356	0.065	0.076	0.019
124	PEO(18:1;0-18:2;0)	Phosphatidylethanolamine (O-18:1_18:2)	36	0.389	0.059	0.083	0.019
125	PEO(18:2;0-18:2;0)	Phosphatidylethanolamine (O-18:2_18:2)	36	0.333	0.066	0.050	0.017

126	PEO(18:2;0-20:4;0)	Phosphatidylethanolamine (O-18:2_20:4)	38	0.337	0.065	0.066	0.020
Lysophosphatidylethanolamines (LPE)							
127	LPE(16:0;0)	Phosphatidylethanolamine (16:0_0:0)	16	0.404	0.064	0.087	0.022
128	LPE(18:1;0)	Phosphatidylethanolamine (18:1_0:0)	18	0.230	0.061	0.058	0.021
129	LPE(18:2;0)	Phosphatidylethanolamine (18:2_0:0)	18	0.191	0.058	0.099	0.020
130	LPE(20:4;0)	Phosphatidylethanolamine (20:4_0:0)	20	0.266	0.063	0.075	0.018
131	LPE(22:6;0)	Phosphatidylethanolamine (22:6_0:0)	22	0.445	0.061	0.076	0.021
Lysophosphatidylcholines (LPC)							
132	LPC(14:0;0)	Phosphatidylcholine (14:0_0:0)	14	0.279	0.060	0.059	0.022
133	LPC(16:0;0)	Phosphatidylcholine (16:0_0:0)	16	0.432	0.060	0.047	0.020
134	LPC(16:1;0)	Phosphatidylcholine (16:1_0:0)	16	0.259	0.059	0.041	0.020
135	LPC(18:0;0)	Phosphatidylcholine (18:0_0:0)	18	0.337	0.064	0.028	0.018
136	LPC(18:1;0)	Phosphatidylcholine (18:1_0:0)	18	0.385	0.065	0.040	0.021
137	LPC(18:2;0)	Phosphatidylcholine (18:2_0:0)	18	0.293	0.063	0.054	0.020
138	LPC(20:3;0)	Phosphatidylcholine (20:3_0:0)	20	0.307	0.067	0.051	0.020
139	LPC(20:4;0)	Phosphatidylcholine (20:4_0:0)	20	0.418	0.067	0.142	0.020
140	LPC(22:6;0)	Phosphatidylcholine (22:6_0:0)	22	0.502	0.062	0.090	0.023
Free cholesterol (ST)							
141	ST	cholesterol	NA	0.258	0.060	0.088	0.021
Traditional Lipids							
142	TG	NA	NA	0.368	0.074	0.150	0.021
143	HDL-C	NA	NA	0.482	0.063	0.170	0.021
144	LDL-C	NA	NA	0.463	0.062	0.152	0.022
145	TC	NA	NA	0.418	0.063	0.174	0.024

Variance explained by known SNPs was estimated for all known SNPs (N=557) for traditional lipid measures (HLD-C, LDL-C, triglycerides and total cholesterol) identified previously through GWASs. *Measured separately through biochemical assays. SE: Standard Error

Supplementary Table 3: List of cardiovascular phenotypes included in the PheWAS from the FinnGen and UK Biobank

S.N.	Phenotype	N (FinnGen)	Number of cases in FinnGen	Number of controls in FinnGen	N (UKBB)	Number of cases in UKBB	Number of controls in UKBB	UKBB GWAS Data Source
1	Stroke	82564	7144	75420	361194	6146	355048	http://www.nealelab.is/uk-biobank/
2	Peripheral atherosclerosis	77601	2181	75420	361194	504	360690	http://www.nealelab.is/uk-biobank/
3	Atrial fibrillation and flutter	63622	7244	56378	395739	14820	380919	ftp://share.sph.umich.edu/UKBB_SAIGE_HRC/
4	Angina pectoris	92142	6382	85760	393278	16175	377103	ftp://share.sph.umich.edu/UKBB_SAIGE_HRC/
5	Arterial embolism and thrombosis	92627	278	92349	401516	921	400595	ftp://share.sph.umich.edu/UKBB_SAIGE_HRC/
6	Atherosclerosis	94545	2196	92349	401919	1324	400595	ftp://share.sph.umich.edu/UKBB_SAIGE_HRC/
7	Atrioventricular [AV] block	75855	718	75137	383044	2125	380919	ftp://share.sph.umich.edu/UKBB_SAIGE_HRC/
8	Cardiac arrest	56721	343	56378	381846	927	380919	ftp://share.sph.umich.edu/UKBB_SAIGE_HRC/
9	Cardiomyopathy	76052	915	75137	407026	1247	405779	ftp://share.sph.umich.edu/UKBB_SAIGE_HRC/
10	Cerebrovascular diseases	96499	5742	90757	407759	8742	399017	ftp://share.sph.umich.edu/UKBB_SAIGE_HRC/
11	Major coronary heart disease event	96499	7123	89376	361194	351037	10157	http://www.nealelab.is/uk-biobank/
12	Coronary atherosclerosis	93421	7661	85760	397126	20023	377103	ftp://share.sph.umich.edu/UKBB_SAIGE_HRC/
13	Heart failure	89269	786	88483	407103	4269	402834	ftp://share.sph.umich.edu/UKBB_SAIGE_HRC/
14	Hypertension	91302	22142	69160	408343	77977	330366	ftp://share.sph.umich.edu/UKBB_SAIGE_HRC/
15	Hypertensive Heart Disease	75971	1626	74345	332085	1719	330366	ftp://share.sph.umich.edu/UKBB_SAIGE_HRC/
16	Intracerebral haemmorrhage	90474	668	89806	408261	9244	399017	ftp://share.sph.umich.edu/UKBB_SAIGE_HRC/
17	Ischaemic heart disease	96499	11139	85360	408458	31355	377103	ftp://share.sph.umich.edu/UKBB_SAIGE_HRC/
18	Myocardial infarction	89825	4065	85760	388806	11703	377103	ftp://share.sph.umich.edu/UKBB_SAIGE_HRC/
19	Peripheral artery disease	94732	2383	92349	405034	4439	400595	ftp://share.sph.umich.edu/UKBB_SAIGE_HRC/
20	Subarachnoid haemmorrhage	90341	576	89765	399829	812	399017	ftp://share.sph.umich.edu/UKBB_SAIGE_HRC/
21	Transient ischemic attack	93589	3378	90211	406815	7798	399017	ftp://share.sph.umich.edu/UKBB_SAIGE_HRC/
22	Venous thromboembolism	96499	3303	93196	361194	4620	356574	http://www.nealelab.is/uk-biobank/
23	Arterial Stiffness	NA	NA	NA	132475	NA	NA	This study
24	Carotid intima media thickness	NA	NA	NA	18510	NA	NA	This study
25	Augmentation index	NA	NA	NA	20910	NA	NA	This study

References

1. Long, T. et al. Whole-genome sequencing identifies common-to-rare variants associated with human blood metabolites. *Nat. Genet.* **49**, 568-578 (2017).
2. Illig, T. et al. A genome-wide perspective of genetic variation in human metabolism. *Nat. Genet.* **42**, 137-141 (2010).
