# Supplementary material for: Genetic architecture of human plasma lipidome and its link to cardiovascular disease
Source: Nat Commun. 2019 Sep 24;10:4329. doi: 10.1038/s41467-019-11954-8 (PMC6760179; doi:10.1038/s41467-019-11954-8)
Supplement: Supplementary file 3 — Reporting Summary [file 41467_2019_11954_MOESM3_ESM.pdf]

nature research

Corresponding author(s): Samuli Ripatti

Last updated by author(s): Aug 5, 2019

Reporting Summary

Nature Research wishes to improve the reproducibility of the work that we publish. This form provides structure for consistency and transparency in reporting. For further information on Nature Research policies, see Authors & Referees and the Editorial Policy Checklist.

Statistics

For all statistical analyses, confirm that the following items are present in the figure legend, table legend, main text, or Methods section.

n/a	Confirmed	
☐	☒	The exact sample size (n) for each experimental group/condition, given as a discrete number and unit of measurement
☐	☒	A statement on whether measurements were taken from distinct samples or whether the same sample was measured repeatedly
☐	☒	The statistical test(s) used AND whether they are one- or two-sided<br>*Only common tests should be described solely by name; describe more complex techniques in the Methods section.*
☐	☒	A description of all covariates tested
☐	☒	A description of any assumptions or corrections, such as tests of normality and adjustment for multiple comparisons
☐	☒	A full description of the statistical parameters including central tendency (e.g. means) or other basic estimates (e.g. regression coefficient) AND variation (e.g. standard deviation) or associated estimates of uncertainty (e.g. confidence intervals)
☐	☒	For null hypothesis testing, the test statistic (e.g. F, t, r) with confidence intervals, effect sizes, degrees of freedom and P value noted<br>*Give P values as exact values whenever suitable.*
☒	☐	For Bayesian analysis, information on the choice of priors and Markov chain Monte Carlo settings
☒	☐	For hierarchical and complex designs, identification of the appropriate level for tests and full reporting of outcomes
☐	☒	Estimates of effect sizes (e.g. Cohen's d, Pearson's r), indicating how they were calculated

Our web collection on statistics for biologists contains articles on many of the points above.

Software and code

Policy information about availability of computer code

Data collection	LipidXplorer (Lipid identification software)
Data analysis	We used following softwares for imputation and association analyses:IMPUTE2, Eagle 2.3.5, Beagle 4.1, biMM, GCTA, SNPTESTv2.5, METAL, PLink2.0, SPAtest R package. We used R 3.3.3 for data transformations, visualization and plotting of the results, including packages ggplot2 and heatmap.2.

For manuscripts utilizing custom algorithms or software that are central to the research but not yet described in published literature, software must be made available to editors/reviewers. We strongly encourage code deposition in a community repository (e.g. GitHub). See the Nature Research guidelines for submitting code & software for further information.

Data

Policy information about availability of data

All manuscripts must include a data availability statement. This statement should provide the following information, where applicable:

- Accession codes, unique identifiers, or web links for publicly available datasets
- A list of figures that have associated raw data
- A description of any restrictions on data availability

The full lipidomics GWAS summary level data are available on the web-based database [https://mqtl.fimm.fi]. Similarly, the PheWAS summary data can be obtained through [https://www.leelabsg.org/resources] and [http://www.nealelab.is/uk-biobank/]. The data presented in the figures and other summary level data are contained within the Supplementary Files and Supplementary Data. Other data are available through the Institute for Molecular Medicine Finland Data Access Committee on reasonable request after appropriate ethical approval.

Field-specific reporting

Please select the one below that is the best fit for your research. If you are not sure, read the appropriate sections before making your selection.

☒ Life sciences ☐ Behavioural & social sciences ☐ Ecological, evolutionary & environmental sciences

For a reference copy of the document with all sections, see nature.com/documents/nr-reporting-summary-flat.pdf

Life sciences study design

All studies must disclose on these points even when the disclosure is negative.

Sample size

We did not perform sample size calculation. The study represent the largest genetic study, to data, of the detailed lipidomic profiles resolved to the acyl chain composition. The study only focus on common variants (>5%) and low-frequency variants (0.5% to 5%) to maximize the statistical power.
The post hoc power calculation show that our sample size of 2,181 indivduals in lipidomics GWAS analyses provided 80% power to detect SNPs with effect size of about 0.14 at genome-wide significance.

Data exclusions

Samples and variants based on standard guidelines and quality control procedures were excluded. The exclusion criteria are detailed in the methods section of the manuscript.

Replication

We performed separate genome-wide association analyses for the cohorts and meta-analyzed the results. Only the results with same directions of effect in the cohorts were considered significant and reported in the manuscript. Moreover, we confirmed the associations at previously identified lipid loci.

Randomization

The study does not include allocation of participants to different experimental groups.

Blinding

As this is an observation study and participants were not allocated to groups , blinding is not relevant to this study.

Reporting for specific materials, systems and methods

We require information from authors about some types of materials, experimental systems and methods used in many studies. Here, indicate whether each material, system or method listed is relevant to your study. If you are not sure if a list item applies to your research, read the appropriate section before selecting a response.

Materials & experimental systems

n/a	Involved in the study	
☒	☐	Antibodies
☒	☐	Eukaryotic cell lines
☒	☐	Palaeontology
☒	☐	Animals and other organisms
☐	☒	Human research participants
☒	☐	Clinical data

Methods

n/a	Involved in the study	
☒	☐	ChIP-seq
☒	☐	Flow cytometry
☒	☐	MRI-based neuroimaging

Human research participants

Policy information about studies involving human research participants

Population characteristics

This study included participants from different study cohorts. The key characteristics of the participants in the cohorts are described in Supplementary Table 1.

Recruitment

Detailed recruitment procedures are provided in the Method sections.

Ethics oversight

The study was conducted in accordance with the principles of the Helsinki declaration. Written informed consent was obtained from all the study participants. The study protocols were approved by the ethics committees of the participating centers (The Hospital District of Helsinki and Uusimaa Coordinating Ethics committees, approval No. 184/13/03/00/12). For the Finnish Institute of Health and Welfare (THL) driven FinnGen preparatory project (here called FinnGen), all patients and control subjects had provided informed consent for biobank research, based on the Finnish Biobank Act. Alternatively, older cohorts were based on study specific consents and later transferred to the THL Biobank after approval by Valvira, the National Supervisory Authority for Welfare and Health. Recruitment protocols followed the biobank protocols approved by Valvira. The Ethical Review Board of the Hospital District of Helsinki and Uusimaa approved the FinnGen study protocol Nr HUS/990/2017. The FinnGen preparatory project is approved by THL, approval numbers THL/2031/6.02.00/2017, amendments THL/341/6.02.00/2018, THL/2222/6.02.00/2018 and THL/283/6.02.00/2019. All DNA samples and data in this study were pseudonymized.

Note that full information on the approval of the study protocol must also be provided in the manuscript.
