# Supplementary material for: Genetic architecture of human plasma lipidome and its link to cardiovascular disease
Source: Nat Commun. 2019 Sep 24;10:4329. doi: 10.1038/s41467-019-11954-8 (PMC6760179; doi:10.1038/s41467-019-11954-8)
Supplement: Supplementary file 4 — Description of Additional Supplementary Files [file 41467_2019_11954_MOESM4_ESM.pdf]

# Description of Additional Supplementary Files

**Supplementary Data 1: Phenotypic and genetic correlation matrix for all lipid species.**

Lower triangle contains pearson's correlation coefficient for pairwise correlation between the plasma levels of lipid species while upper triangle presents genetic correlation values as calculated using biMM.

**Supplementary Data 2: List of all genome-wide significant associations ($P<5.0x10^{-8}$) between SNPs and lipid species in our study.**

Variants are designated as new if not located within 1Mb of any previously reported variants for lipids (any of the traditional lipid measures and molecular lipid species); and as independent signal in known locus if located within 1 Mb but $r^2<0.20$ with the previous lead variants and confirmed by conditional analysis.

**Supplementary Data 3: Association statistics for the lead variants in 35 genome-wide significant loci.**

Association statistics for the lead variants in the 35 identified loci in all the three cohorts separately and meta-analyses are provided along with the conditional analyses with the known variants in the loci and analyses adjusted for traditional lipids.

**Supplementary Data 4: Associations of lipid species associated loci with lipid related metabolites in previous metabolomics GWAS.**

All the previously reported association at the identified loci are presented. Out of the 35 identified loci, 11 loci were reported to associate with at least one of the lipid related metabolites analyzed in any previous mGWAS at $P<5.0x10^{-8}$.

**Supplementary Data 5: Association of 141 lipid species with known variants identified in previous metabolomics GWAS.**

The associations of variants previously identified in metabolomics GWAS with 141 lipid species in our study are presented. A total of 126 variants from 46 loci were available in our dataset out of 132 reported variants.

**Supplementary Data 6: Comparison of SNP-metabolite associations between previous metabolomics GWAS and the current study.**

The comparison of associations of 134 SNP-metabolite pairs identified in previous GWASs with that of observed in our study are presented.

**Supplementary Data 7. Regional plots for lipid species associated loci**.

The regional plots showing associations of variants within 2Mb region of the identified loci with all lipid species. Only the associations with $P<1.0 \times 10^{-2}$ for meta-analyses of genome-wide association analyses in three batch and consistent in the directions of effects are shown.

**Supplementary Data 8: Association of lead variants in lipid species associated loci with CVD outcomes in PheWAS.**

Association of the lead variants in the identified loci with CVD related outcomes in the FinnGen and UKBB datasets and their meta-analyses are presented.

**Supplementary Data 9: List of variants that have previously been associated with traditional lipids and were included in the present study.**

All the variants that are identified through previous GWASs for traditional lipids (HDL-C, LDL-C, triglycerides and total cholesterol) and available in our dataset are listed.

**Supplementary Data 10: Associations between lipid species and 557 known variants previously identified for traditional lipids.**

Associations of 557 known variants listed in Supplementary Data 9, with 141 lipid species were evaluated. All associations with $P<0.05$ are listed.

**Supplementary Data 11: Effect of fasting duration on the plasma levels of molecular lipid species in the FINRISK cohort.**

The median plasma levels of lipid species between FINRISK samples fasted over 6 hours and FINRISK samples fasted less than 6 hours are compared.

**Supplementary Data 12: List of lipids detected by different platforms- Metabolon, Biocrates and Lipotype.**

Lipid species detected by three commonly used platforms- Metabolon, Biocrates and Lipotype are compared.

**Supplementary Data 13: Genomic inflation factor (λ) for association statistics of three batches and meta-analysis.**

Genomic inflation factor (λ) in all the three batches and meta-analyses for all the lipid species are presented.
