# Supplementary material for: Genetic architecture of human plasma lipidome and its link to cardiovascular disease
Source: Nat Commun. 2019 Sep 24;10:4329. doi: 10.1038/s41467-019-11954-8 (PMC6760179; doi:10.1038/s41467-019-11954-8)
Supplement: Supplementary file 11 — Supplementary Data 7 [file 41467_2019_11954_MOESM11_ESM.pdf]

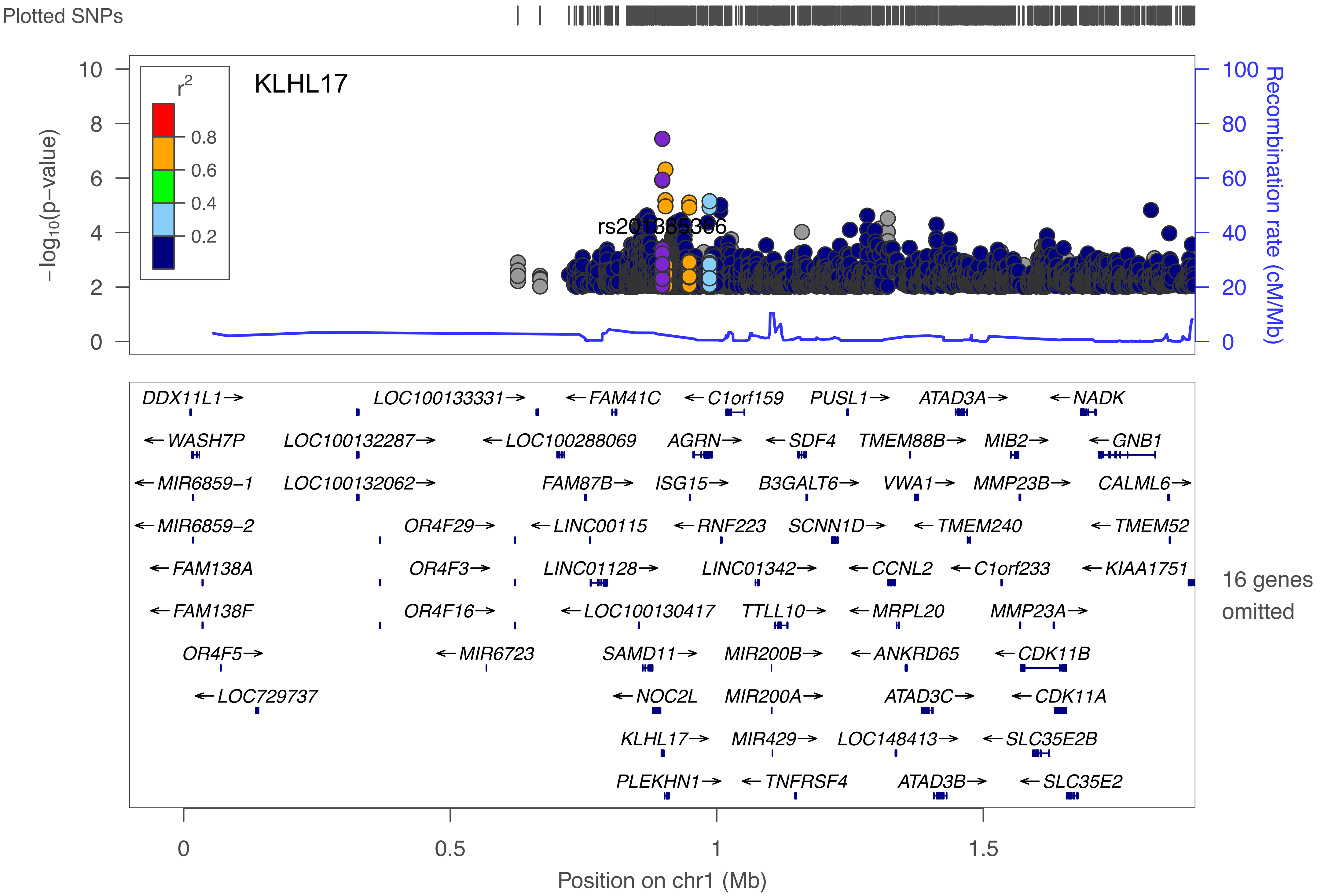

Plotted SNPs
KLHL17
r2
0.8
0.6
0.4
0.2
rs201385366
−log10(p−value)
Recombination rate (cM/Mb)
10
8
6
4
2
0
100
80
60
40
20
0
DDX11L1→
LOC100133331→
←FAM41C
←C1orf159
PUSL1→
ATAD3A→
←NADK
←WASH7P
LOC100132287→
←LOC100288069
AGRN→
←SDF4
TMEM88B→
MIB2→
←GNB1
←MIR6859−1
LOC100132062→
FAM87B→
ISG15→
B3GALT6→
VWA1→
MMP23B→
CALML6→
←MIR6859−2
OR4F29→
←LINC00115
←RNF223
SCNN1D→
←TMEM240
←TMEM52
←FAM138A
OR4F3→
LINC01128→
LINC01342→
←CCNL2
←C1orf233
←KIAA1751
←FAM138F
OR4F16→
←LOC100130417
TTLL10→
←MRPL20
MMP23A→
OR4F5→
←MIR6723
SAMD11→
MIR200B→
←ANKRD65
←CDK11B
←LOC729737
←NOC2L
MIR200A→
ATAD3C→
←CDK11A
KLHL17→
MIR429→
LOC148413→
←SLC35E2B
PLEKHN1→
←TNFRSF4
ATAD3B→
←SLC35E2
16 genes omitted
0
0.5
1
1.5
Position on chr1 (Mb)

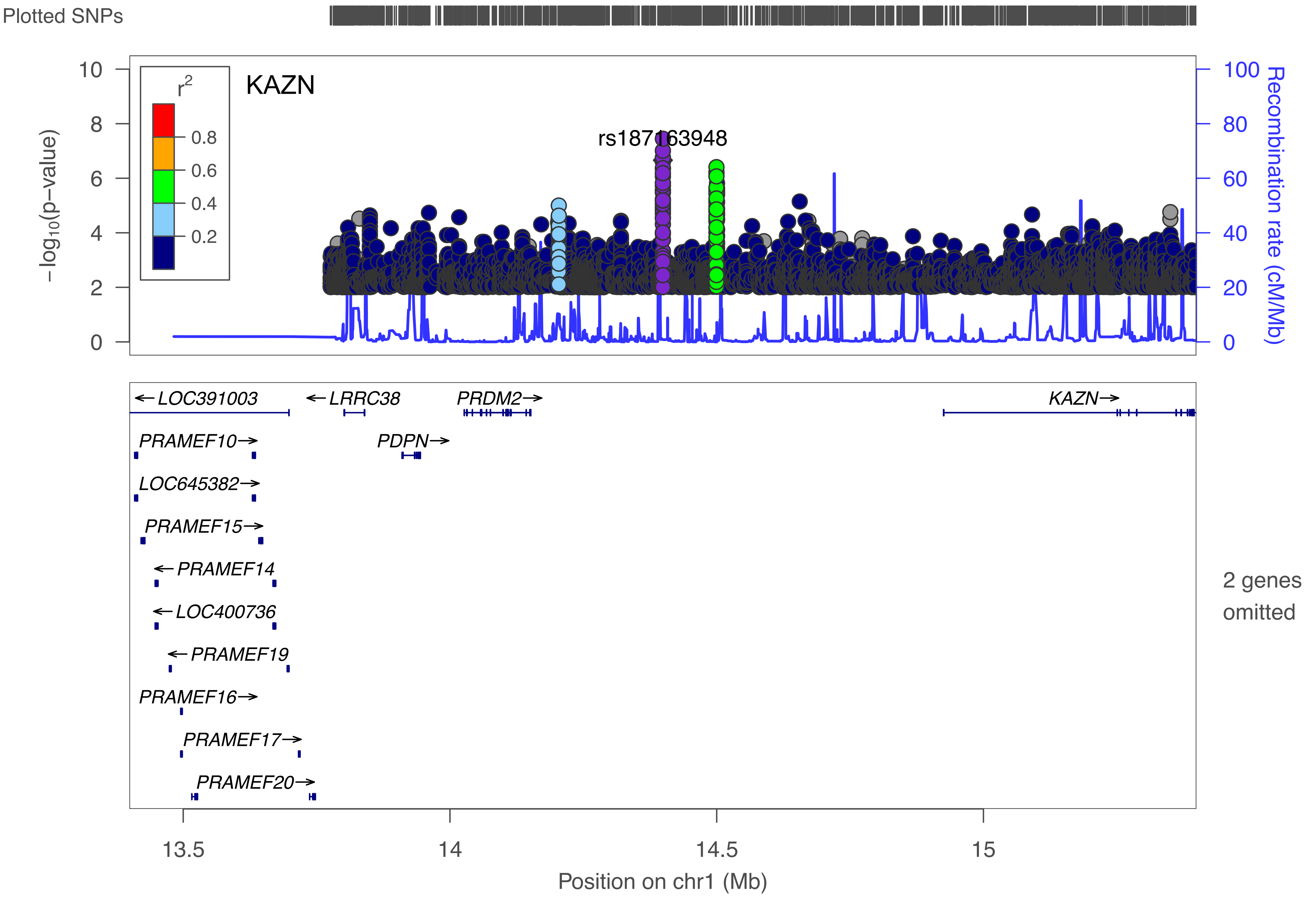

Plotted SNPs
KAZN
rs187163948
r2
0.8
0.6
0.4
0.2
−log10(p−value)
Recombination rate (cM/Mb)
LOC391003
LRRC38
PRDM2
KAZN
PRAMEF10
PDPN
LOC645382
PRAMEF15
PRAMEF14
LOC400736
PRAMEF19
PRAMEF16
PRAMEF17
PRAMEF20
2 genes omitted
13.5
14
14.5
15
Position on chr1 (Mb)

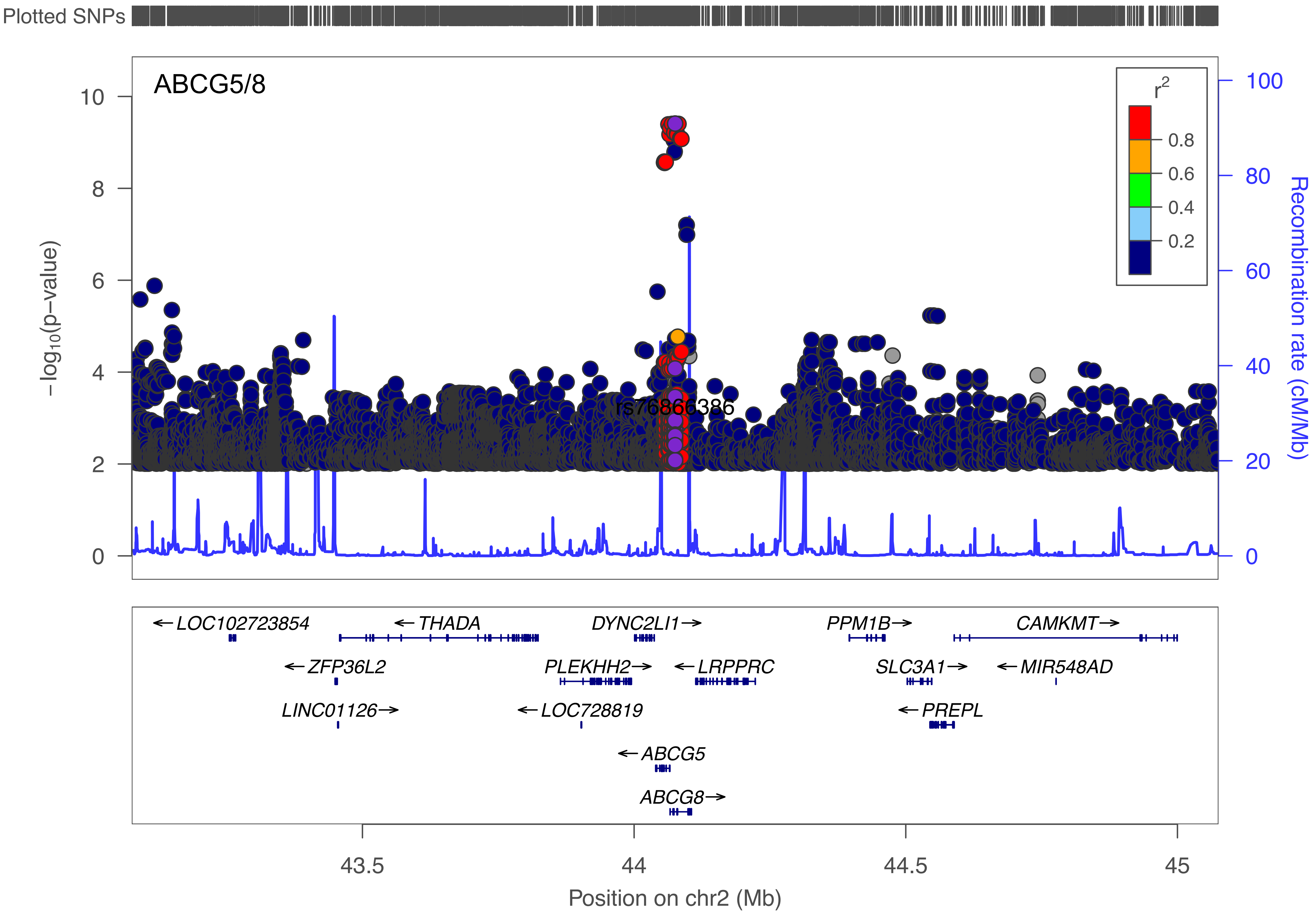
Plotted SNPs
ABCG5/8
−log10(p−value)
Recombination rate (cM/Mb)
r2
0.8
0.6
0.4
0.2
rs76866386
←LOC102723854
←THADA
DYNC2LI1→
PPM1B→
CAMKMT→
←ZFP36L2
PLEKHH2→
←LRPPRC
SLC3A1→
←MIR548AD
LINC01126→
←LOC728819
←PREPL
←ABCG5
ABCG8→
43.5
44
44.5
45
Position on chr2 (Mb)

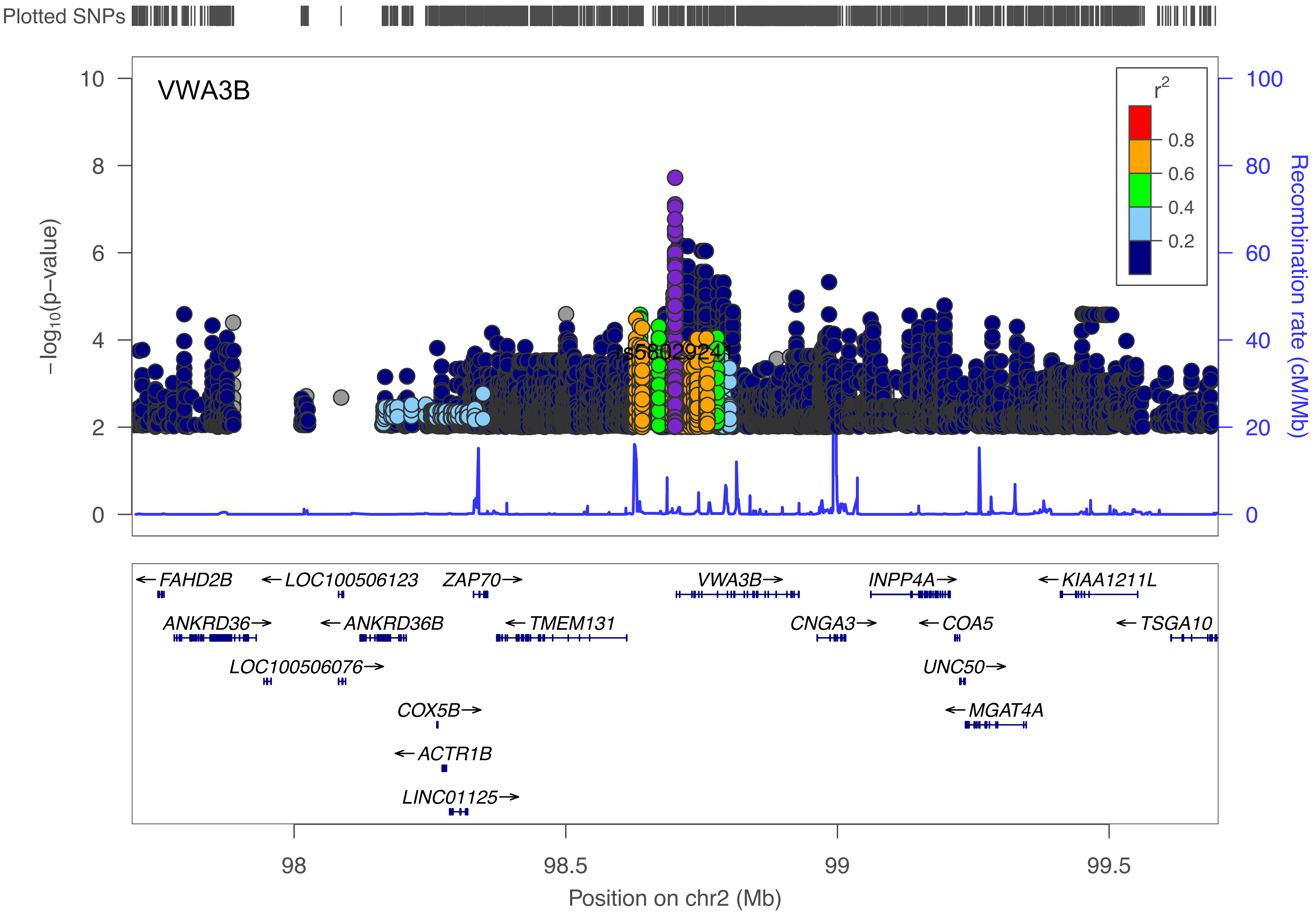

Plotted SNPs
VWA3B
r2
0.8
0.6
0.4
0.2
rs58029241
−log10(p−value)
Recombination rate (cM/Mb)
10
8
6
4
2
0
100
80
60
40
20
0
FAHD2B
LOC100506123
ZAP70
VWA3B
INPP4A
KIAA1211L
ANKRD36
ANKRD36B
TMEM131
CNGA3
COA5
TSGA10
LOC100506076
UNC50
COX5B
MGAT4A
ACTR1B
LINC01125
98
98.5
99
99.5
Position on chr2 (Mb)

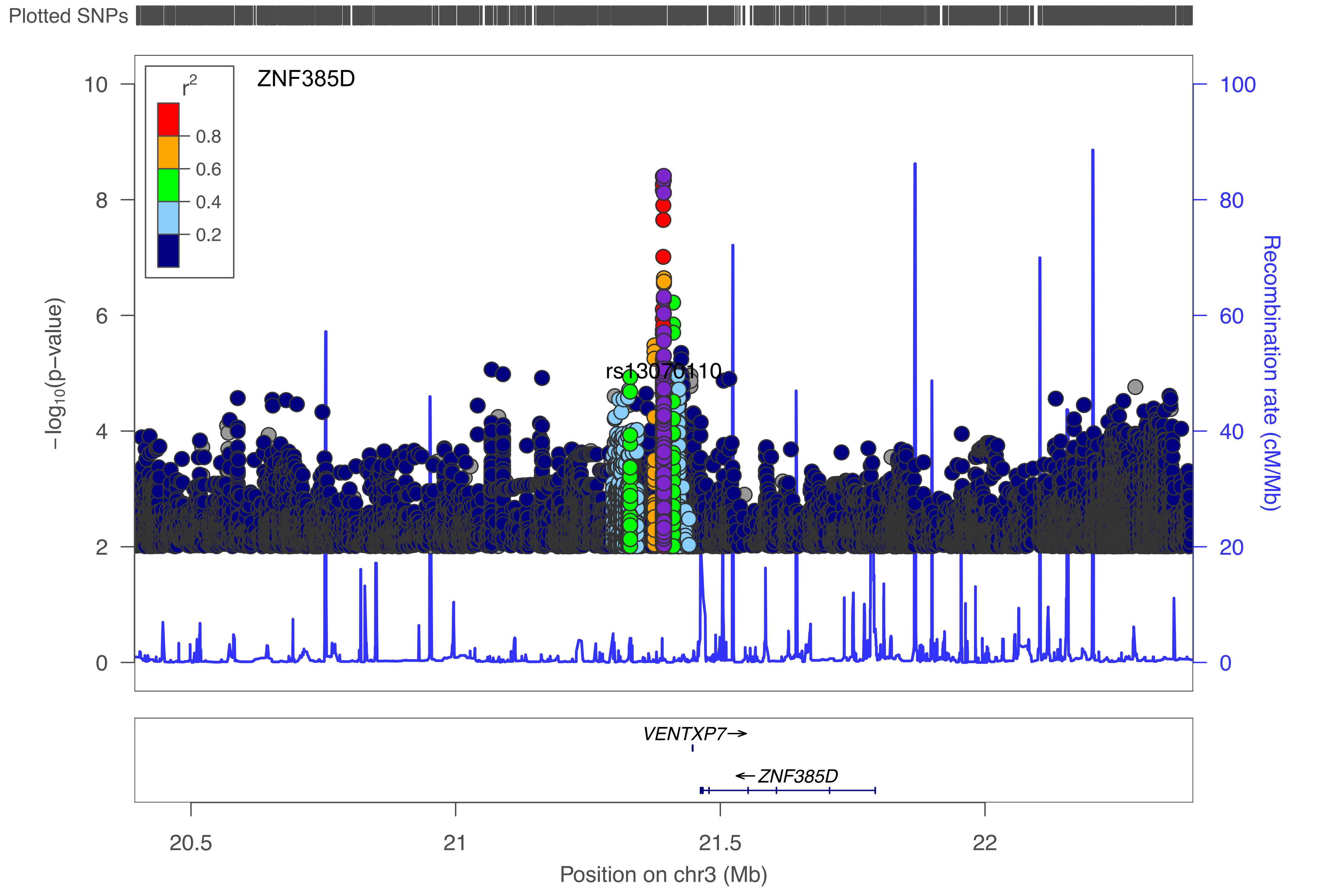

Plotted SNPs
ZNF385D
r2
0.8
0.6
0.4
0.2
rs13070110
−log10(p−value)
Recombination rate (cM/Mb)
VENTXP7→
←ZNF385D
20.5
21
21.5
22
Position on chr3 (Mb)

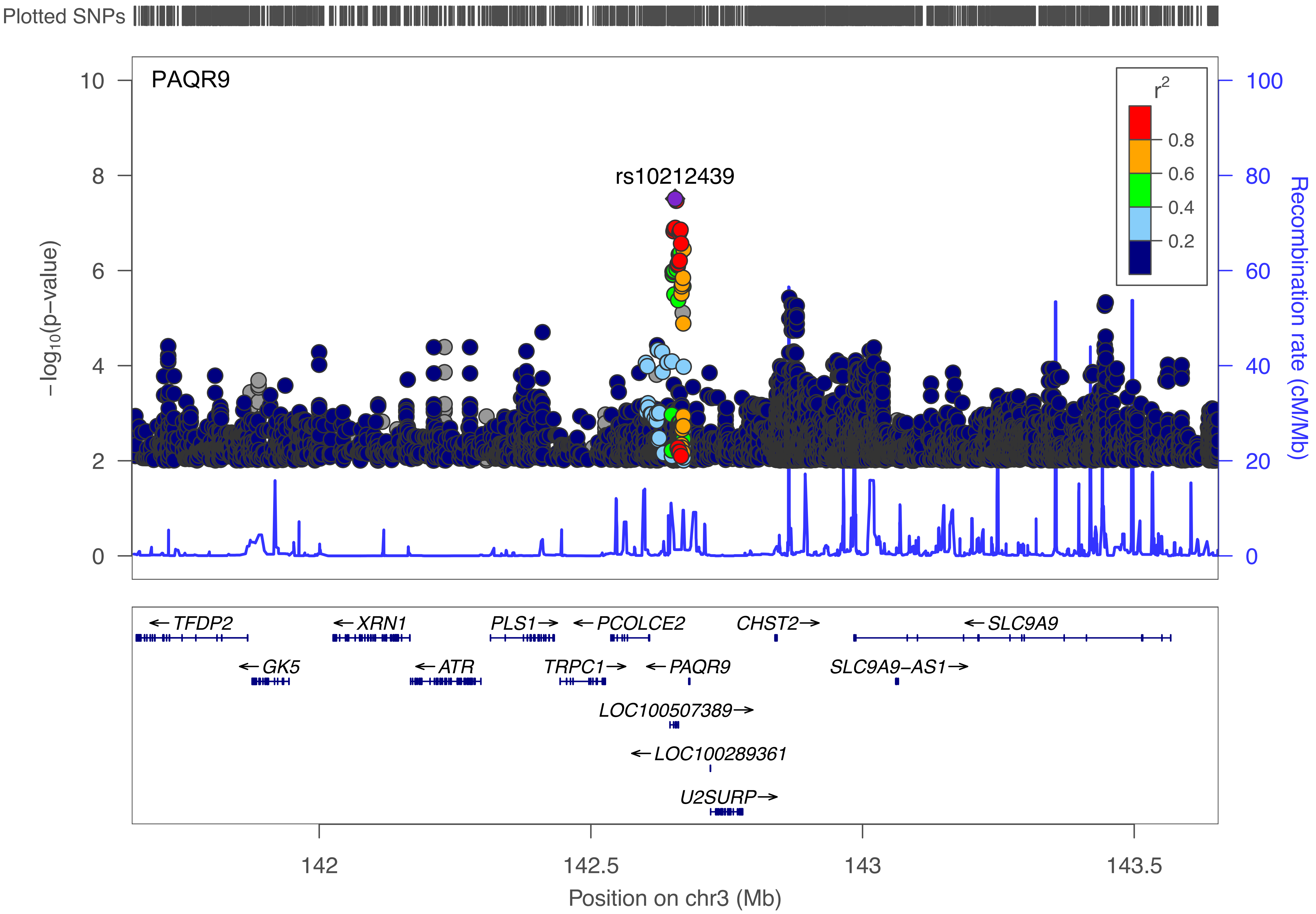

Plotted SNPs
PAQR9
rs10212439
r2
0.8
0.6
0.4
0.2
10
8
6
4
2
0
100
80
60
40
20
0
−log10(p−value)
Recombination rate (cM/Mb)
←TFDP2
←XRN1
PLS1→
←PCOLCE2
CHST2→
←SLC9A9
←GK5
←ATR
TRPC1→
←PAQR9
SLC9A9−AS1→
LOC100507389→
←LOC100289361
U2SURP→
142
142.5
143
143.5
Position on chr3 (Mb)

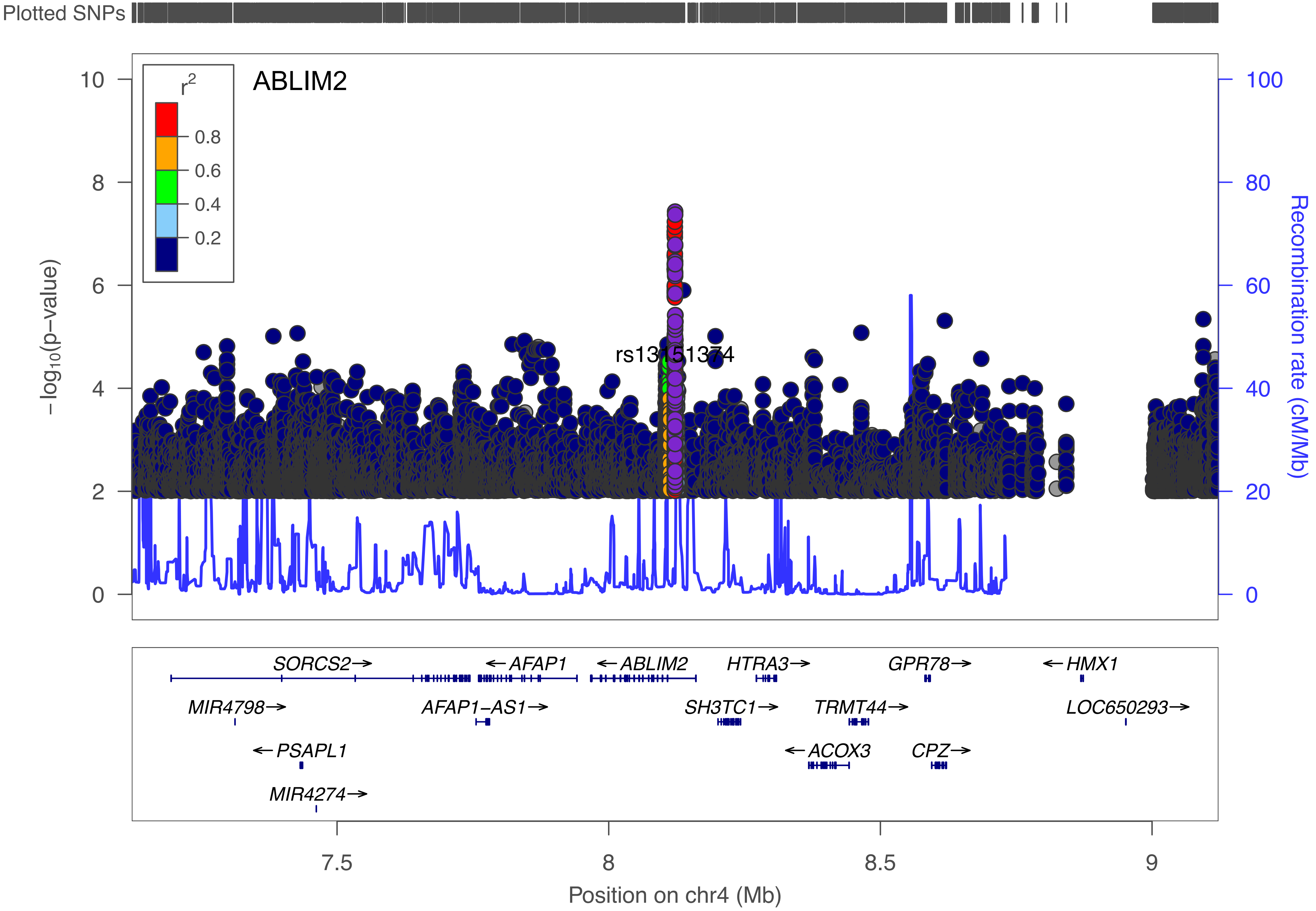

Plotted SNPs
ABLIM2
r2
0.8
0.6
0.4
0.2
rs13151374
−log10(p−value)
Recombination rate (cM/Mb)
10
8
6
4
2
0
100
80
60
40
20
0
SORCS2→
←AFAP1
←ABLIM2
HTRA3→
GPR78→
←HMX1
MIR4798→
AFAP1−AS1→
SH3TC1→
TRMT44→
LOC650293→
←PSAPL1
←ACOX3
CPZ→
MIR4274→
7.5
8
8.5
9
Position on chr4 (Mb)

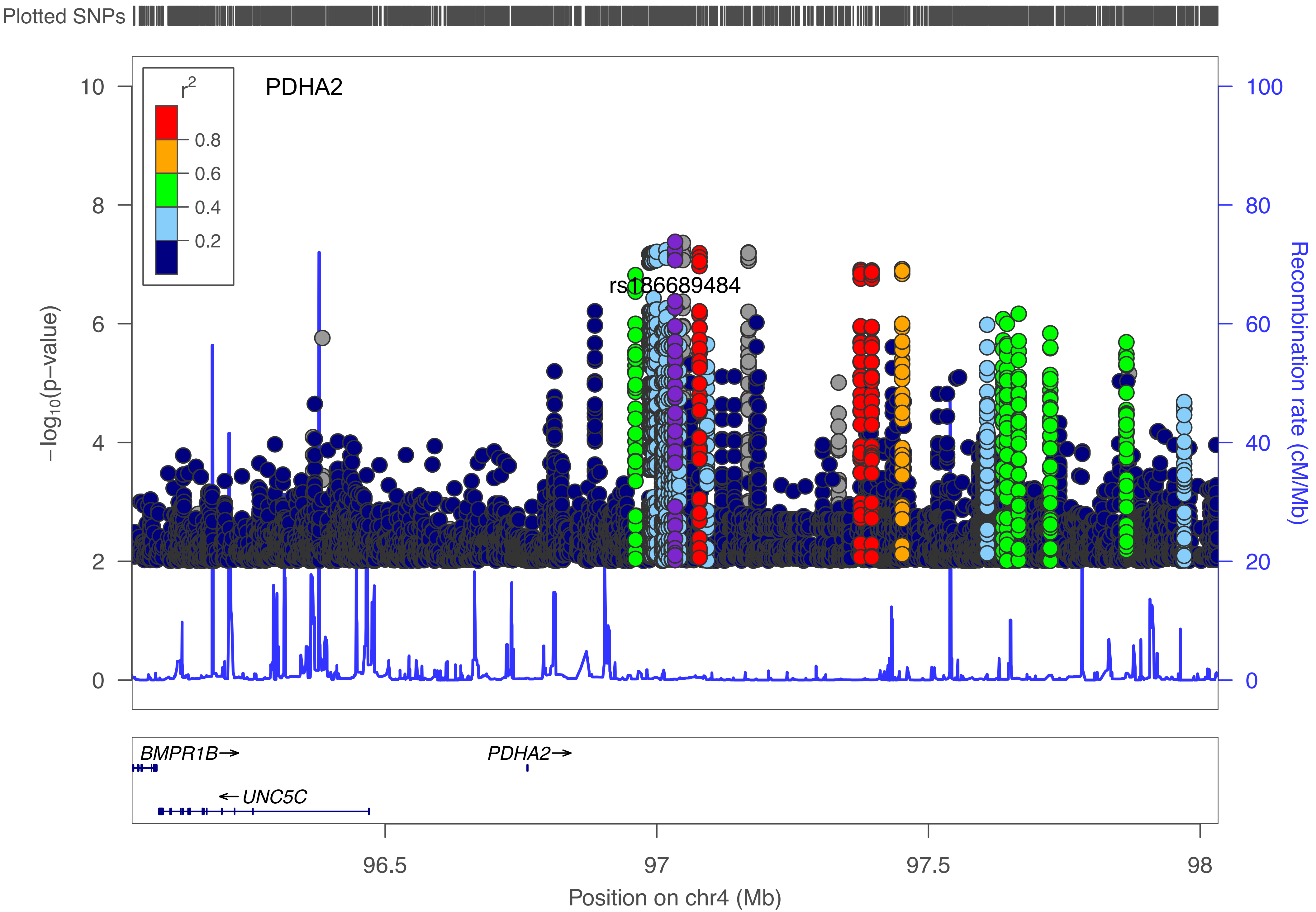

Plotted SNPs
PDHA2
r2
0.8
0.6
0.4
0.2
rs186689484
−log10(p−value)
Recombination rate (cM/Mb)
0
2
4
6
8
10
0
20
40
60
80
100
BMPR1B→
PDHA2→
←UNC5C
96.5
97
97.5
98
Position on chr4 (Mb)

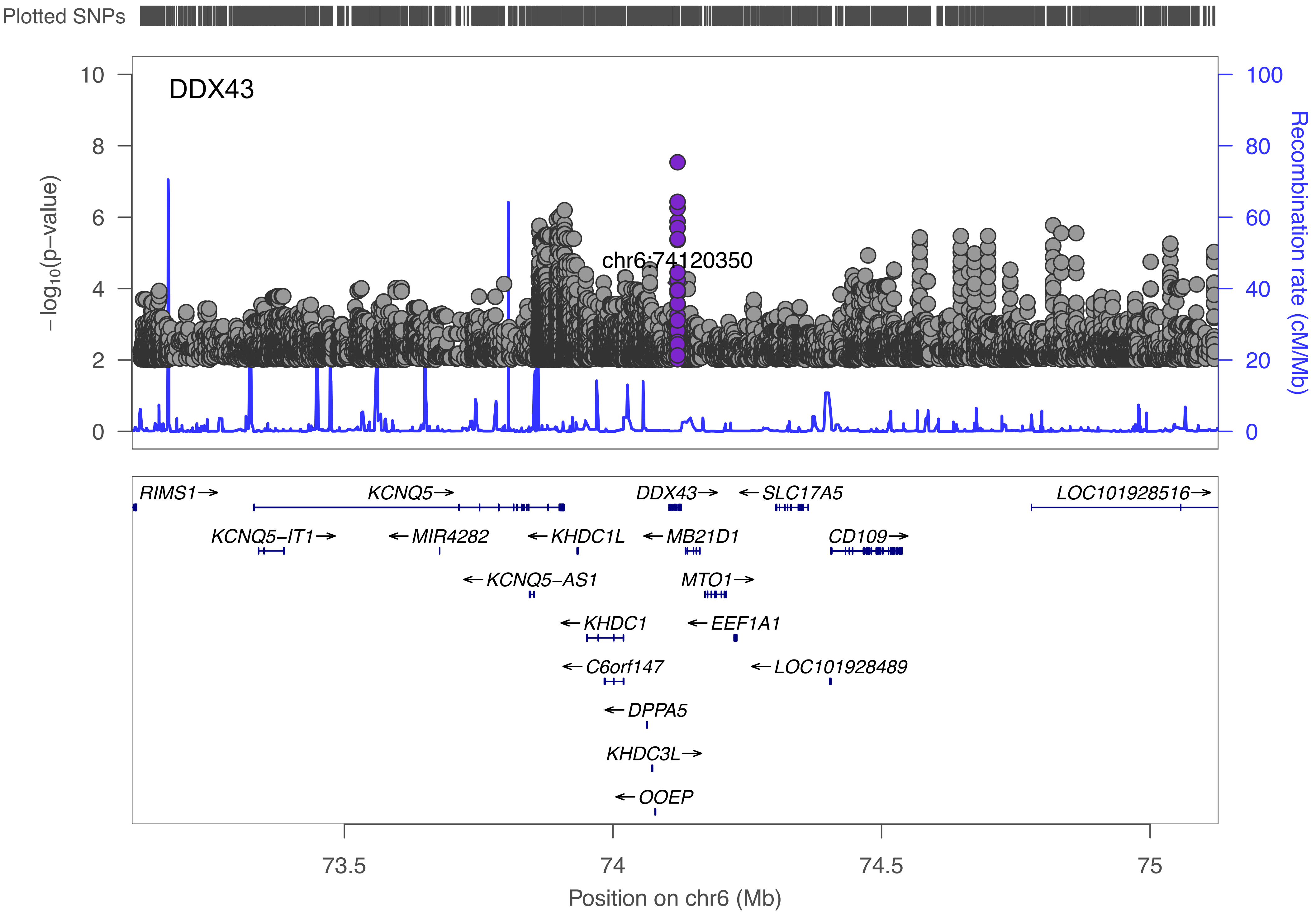
Plotted SNPs
DDX43
chr6:74120350
-log10(p-value)
Recombination rate (cM/Mb)
10
8
6
4
2
0
100
80
60
40
20
0
RIMS1→
KCNQ5→
DDX43→
←SLC17A5
LOC101928516→
KCNQ5-IT1→
←MIR4282
←KHDC1L
←MB21D1
CD109→
←KCNQ5-AS1
MTO1→
←KHDC1
←EEF1A1
←C6orf147
←LOC101928489
←DPPA5
KHDC3L→
←OOEP
73.5
74
74.5
75
Position on chr6 (Mb)

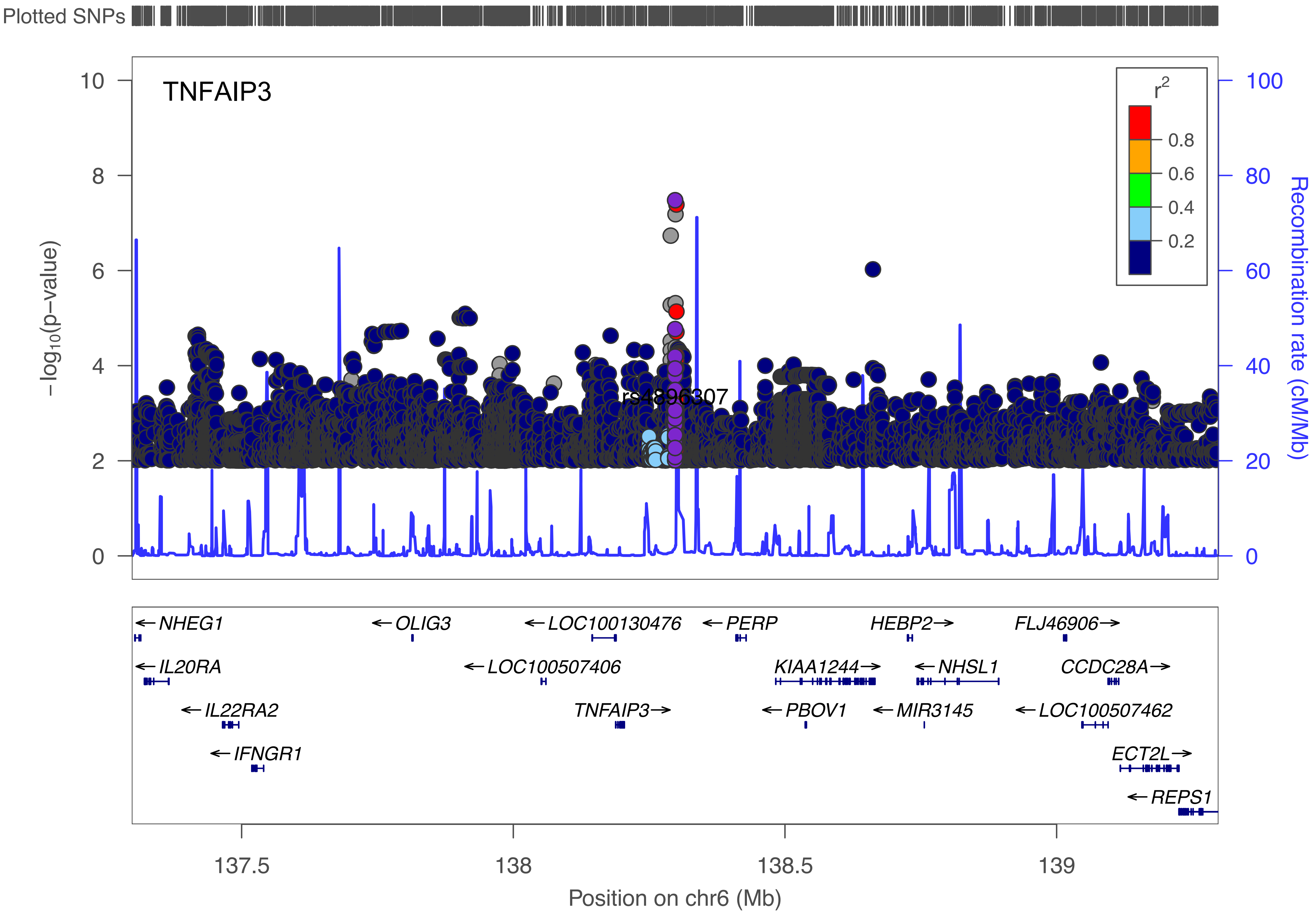

Plotted SNPs
TNFAIP3
r2
0.8
0.6
0.4
0.2
rs4896307
−log10(p−value)
Recombination rate (cM/Mb)
10
8
6
4
2
0
100
80
60
40
20
0
NHEG1
IL20RA
IL22RA2
IFNGR1
OLIG3
LOC100130476
LOC100507406
TNFAIP3
PERP
KIAA1244
PBOV1
HEBP2
NHSL1
MIR3145
FLJ46906
CCDC28A
LOC100507462
ECT2L
REPS1
137.5
138
138.5
139
Position on chr6 (Mb)

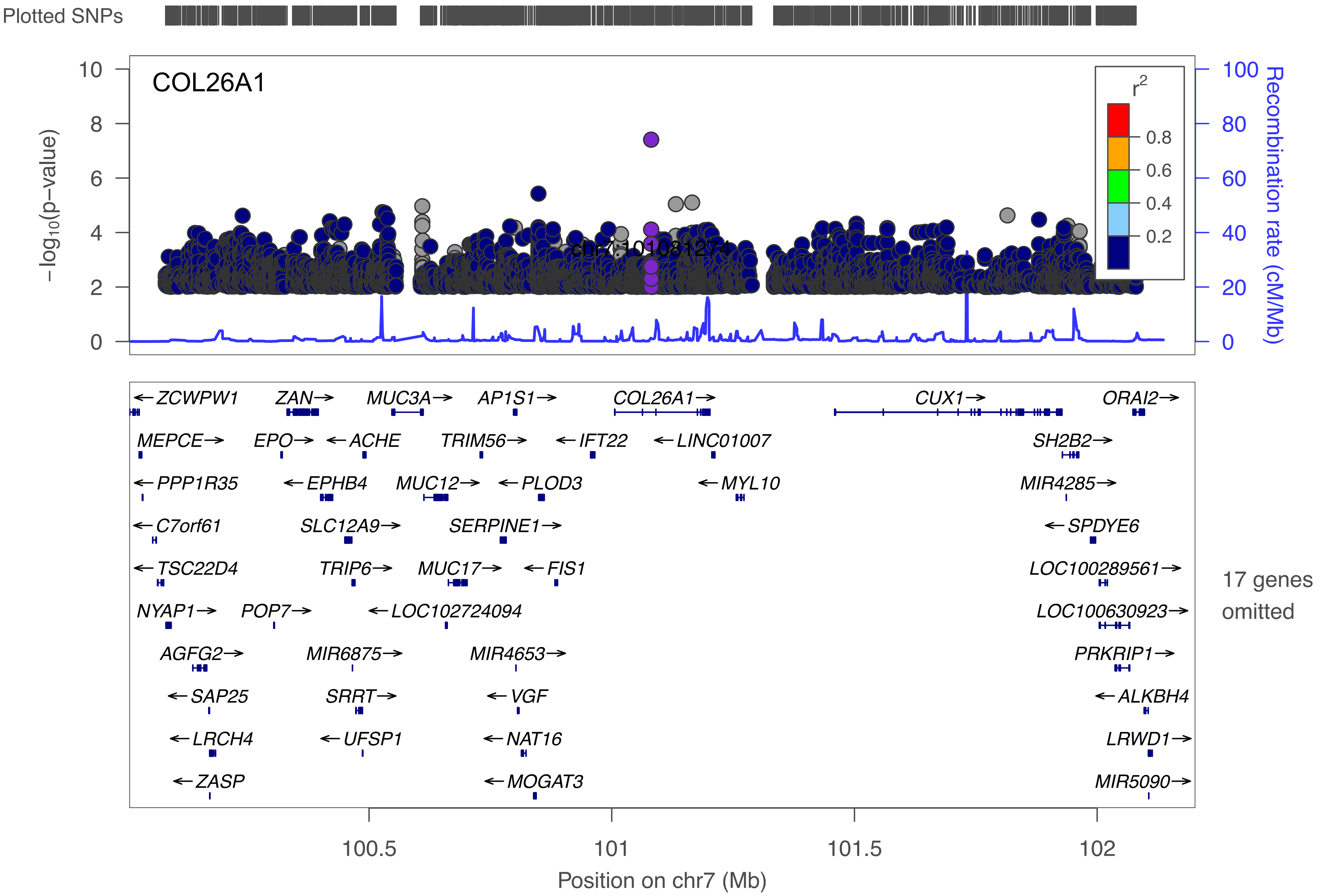

Plotted SNPs
COL26A1
chr7:101081274
r2
0.8
0.6
0.4
0.2
−log10(p−value)
Recombination rate (cM/Mb)
ZCWPW1
ZAN
MUC3A
AP1S1
COL26A1
CUX1
ORAI2
MEPCE
EPO
ACHE
TRIM56
IFT22
LINC01007
SH2B2
PPP1R35
EPHB4
MUC12
PLOD3
MYL10
MIR4285
C7orf61
SLC12A9
SERPINE1
SPDYE6
TSC22D4
TRIP6
MUC17
FIS1
LOC100289561
NYAP1
POP7
LOC102724094
LOC100630923
AGFG2
MIR6875
MIR4653
PRKRIP1
SAP25
SRRT
VGF
ALKBH4
LRCH4
UFSP1
NAT16
LRWD1
ZASP
MOGAT3
MIR5090
17 genes omitted
100.5
101
101.5
102
Position on chr7 (Mb)

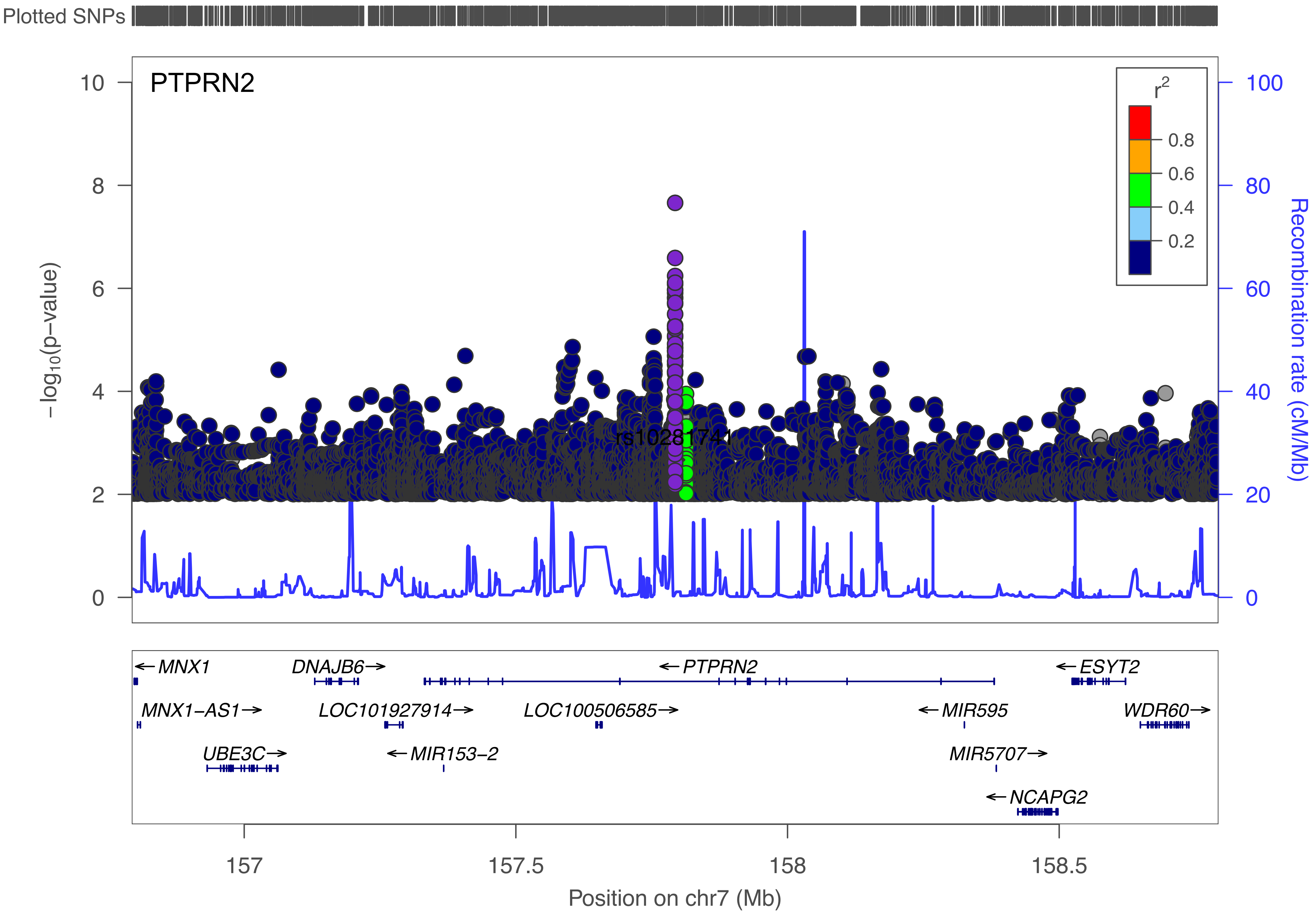

Plotted SNPs
PTPRN2
r2
0.8
0.6
0.4
0.2
rs10281741
10
8
6
4
2
0
100
80
60
40
20
0
−log10(p−value)
Recombination rate (cM/Mb)
MNX1
DNAJB6
PTPRN2
ESYT2
MNX1−AS1
LOC101927914
LOC100506585
MIR595
WDR60
UBE3C
MIR153−2
MIR5707
NCAPG2
157
157.5
158
158.5
Position on chr7 (Mb)

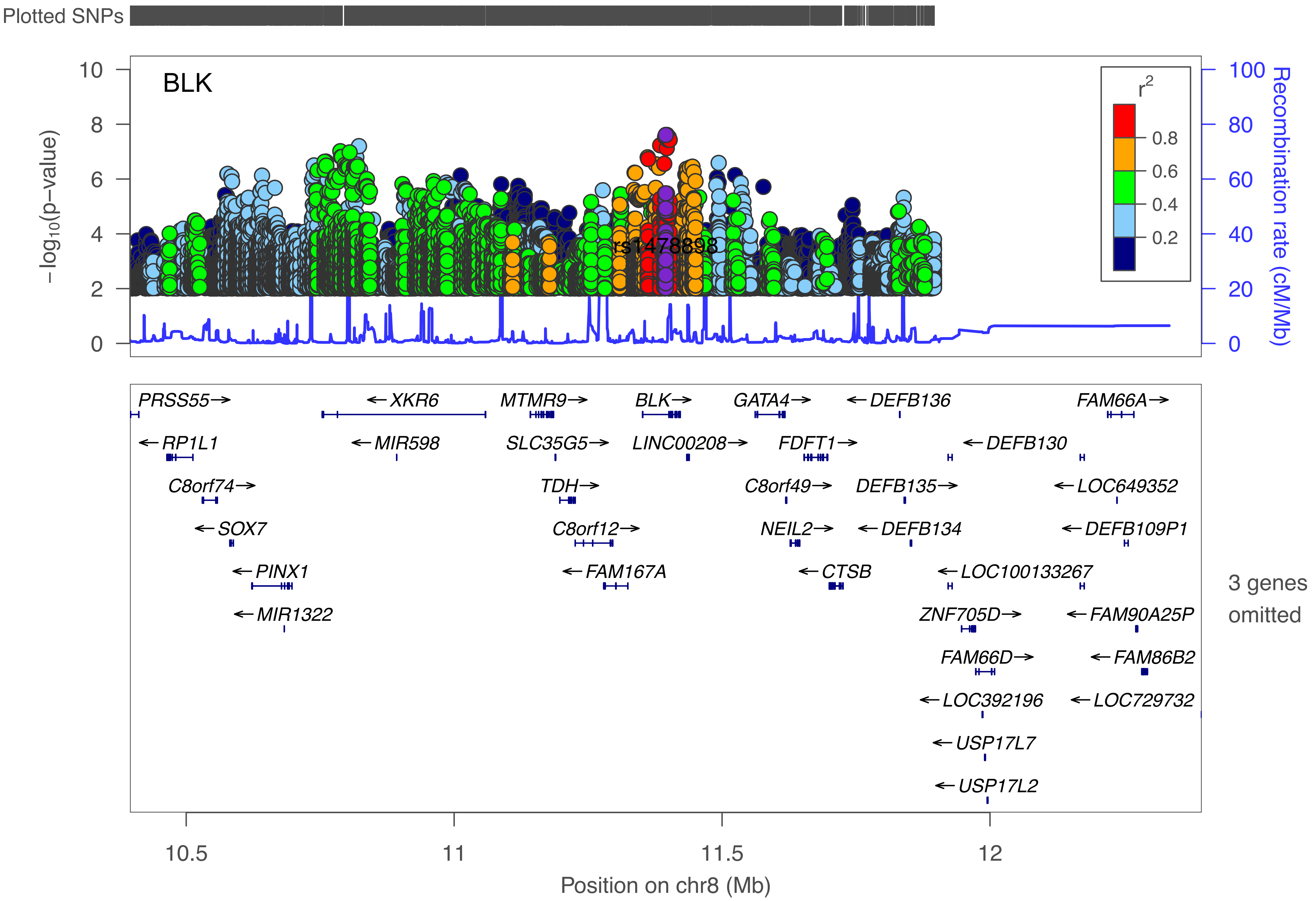

Plotted SNPs
BLK
rs1478898
r2
0.8
0.6
0.4
0.2
−log10(p−value)
Recombination rate (cM/Mb)
10
8
6
4
2
0
100
80
60
40
20
0
PRSS55→
←XKR6
MTMR9→
BLK→
GATA4→
←DEFB136
FAM66A→
←RP1L1
←MIR598
SLC35G5→
LINC00208→
FDFT1→
←DEFB130
C8orf74→
TDH→
C8orf49→
DEFB135→
←LOC649352
←SOX7
C8orf12→
NEIL2→
←DEFB134
←DEFB109P1
←PINX1
←FAM167A
←CTSB
←LOC100133267
←MIR1322
ZNF705D→
←FAM90A25P
FAM66D→
←FAM86B2
←LOC392196
←LOC729732
←USP17L7
←USP17L2
3 genes omitted
10.5
11
11.5
12
Position on chr8 (Mb)

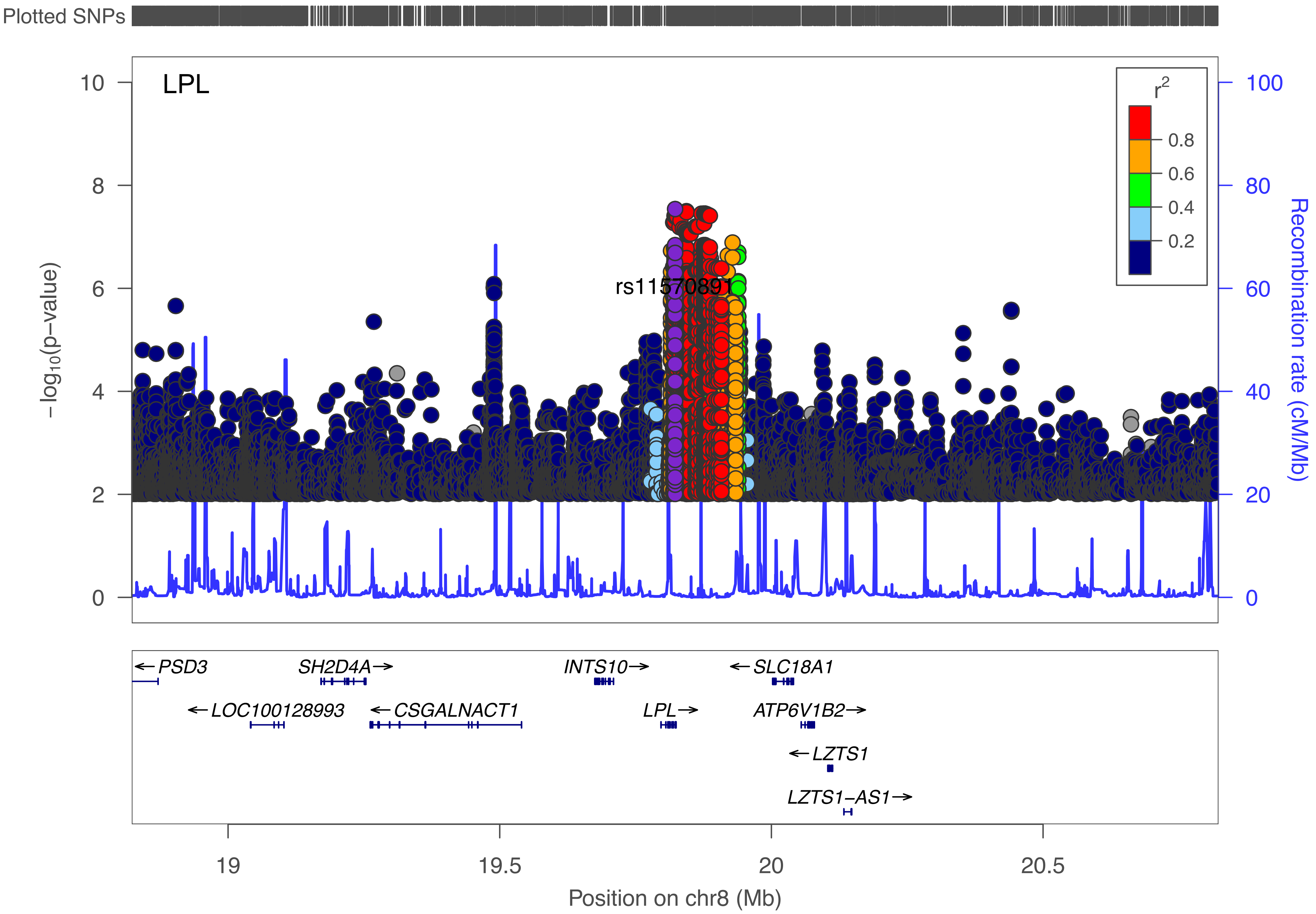
Plotted SNPs
LPL
rs11570891
r2
0.8
0.6
0.4
0.2
10
8
6
4
2
0
100
80
60
40
20
0
−log10(p−value)
Recombination rate (cM/Mb)
←PSD3
SH2D4A→
INTS10→
←SLC18A1
←LOC100128993
←CSGALNACT1
LPL→
ATP6V1B2→
←LZTS1
LZTS1−AS1→
19
19.5
20
20.5
Position on chr8 (Mb)

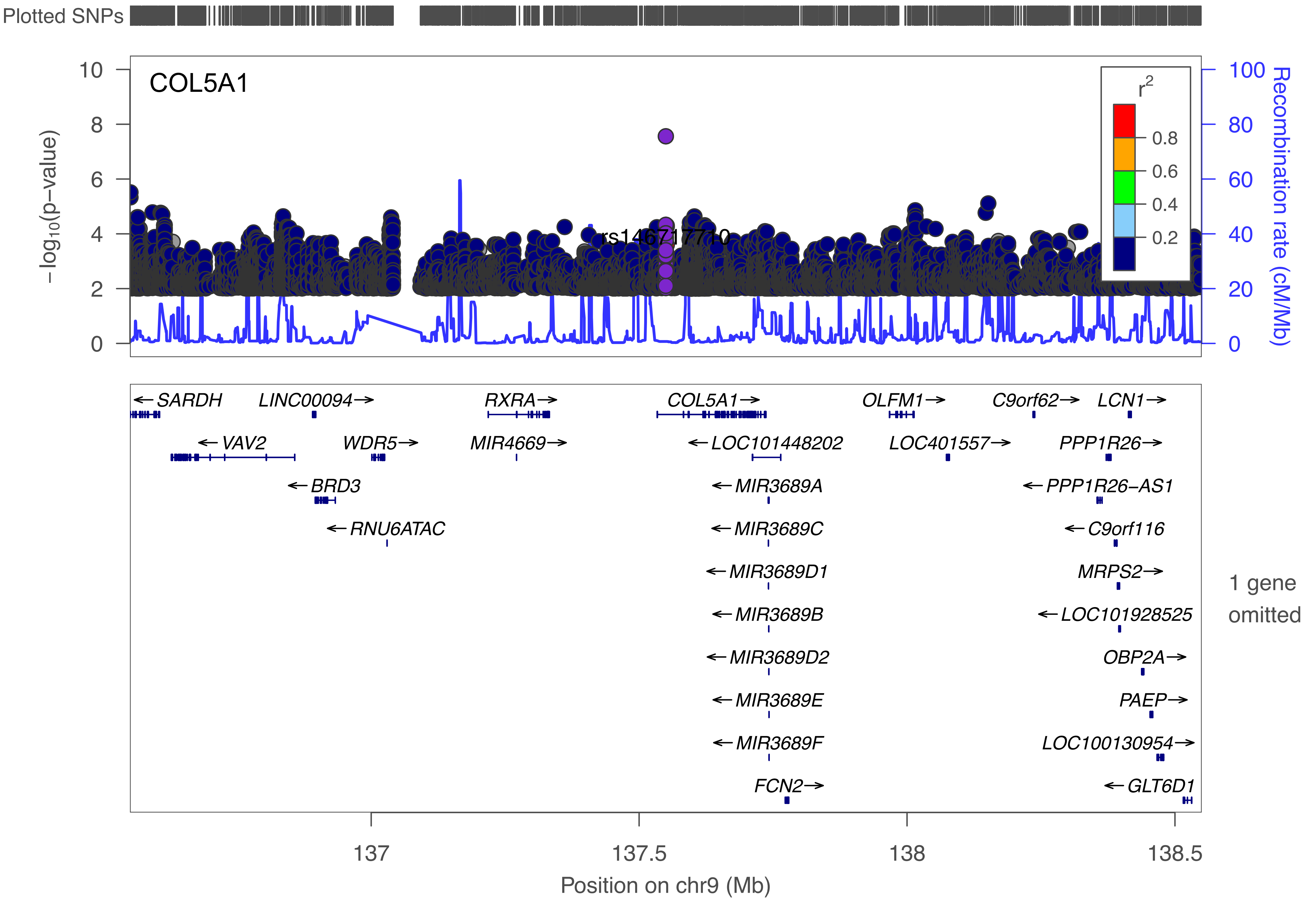

Plotted SNPs
COL5A1
rs146717710
r2
0.8
0.6
0.4
0.2
10
8
6
4
2
0
−log10(p−value)
100
80
60
40
20
0
Recombination rate (cM/Mb)
← SARDH
LINC00094 →
RXRA →
COL5A1 →
OLFM1 →
C9orf62 →
LCN1 →
← VAV2
WDR5 →
MIR4669 →
← LOC101448202
LOC401557 →
PPP1R26 →
← BRD3
← MIR3689A
← PPP1R26−AS1
← RNU6ATAC
← MIR3689C
← C9orf116
← MIR3689D1
MRPS2 →
1 gene omitted
← MIR3689B
← LOC101928525
← MIR3689D2
OBP2A →
← MIR3689E
PAEP →
← MIR3689F
LOC100130954 →
FCN2 →
← GLT6D1
137
137.5
138
138.5
Position on chr9 (Mb)

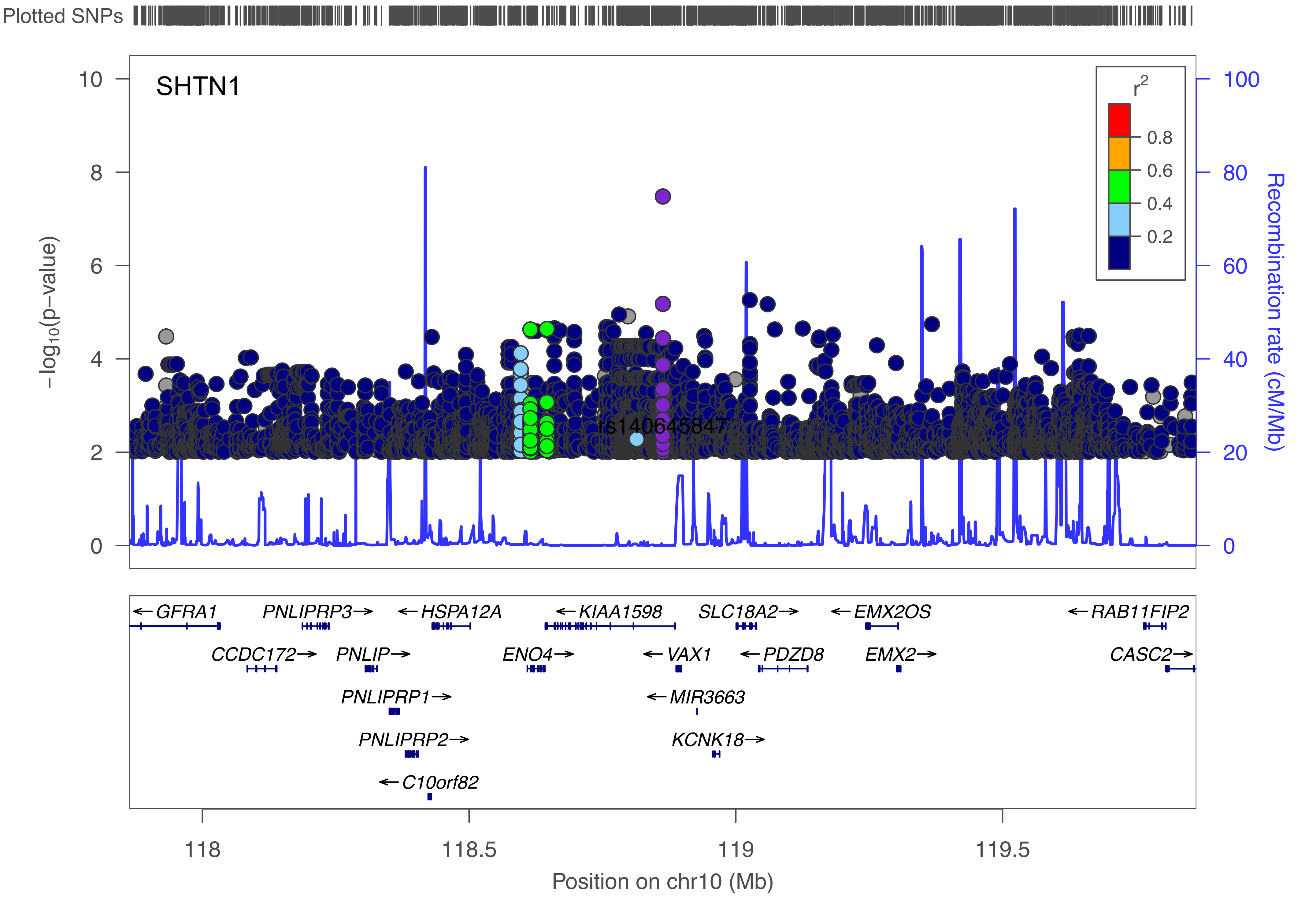

Plotted SNPs
SHTN1
r2
0.8
0.6
0.4
0.2
rs140645847
10
8
6
4
2
0
100
80
60
40
20
0
-log10(p-value)
Recombination rate (cM/Mb)
GFRA1
PNLIPRP3
HSPA12A
KIAA1598
SLC18A2
EMX2OS
RAB11FIP2
CCDC172
PNLIP
ENO4
VAX1
PDZD8
EMX2
CASC2
PNLIPRP1
MIR3663
PNLIPRP2
KCNK18
C10orf82
118
118.5
119
119.5
Position on chr10 (Mb)

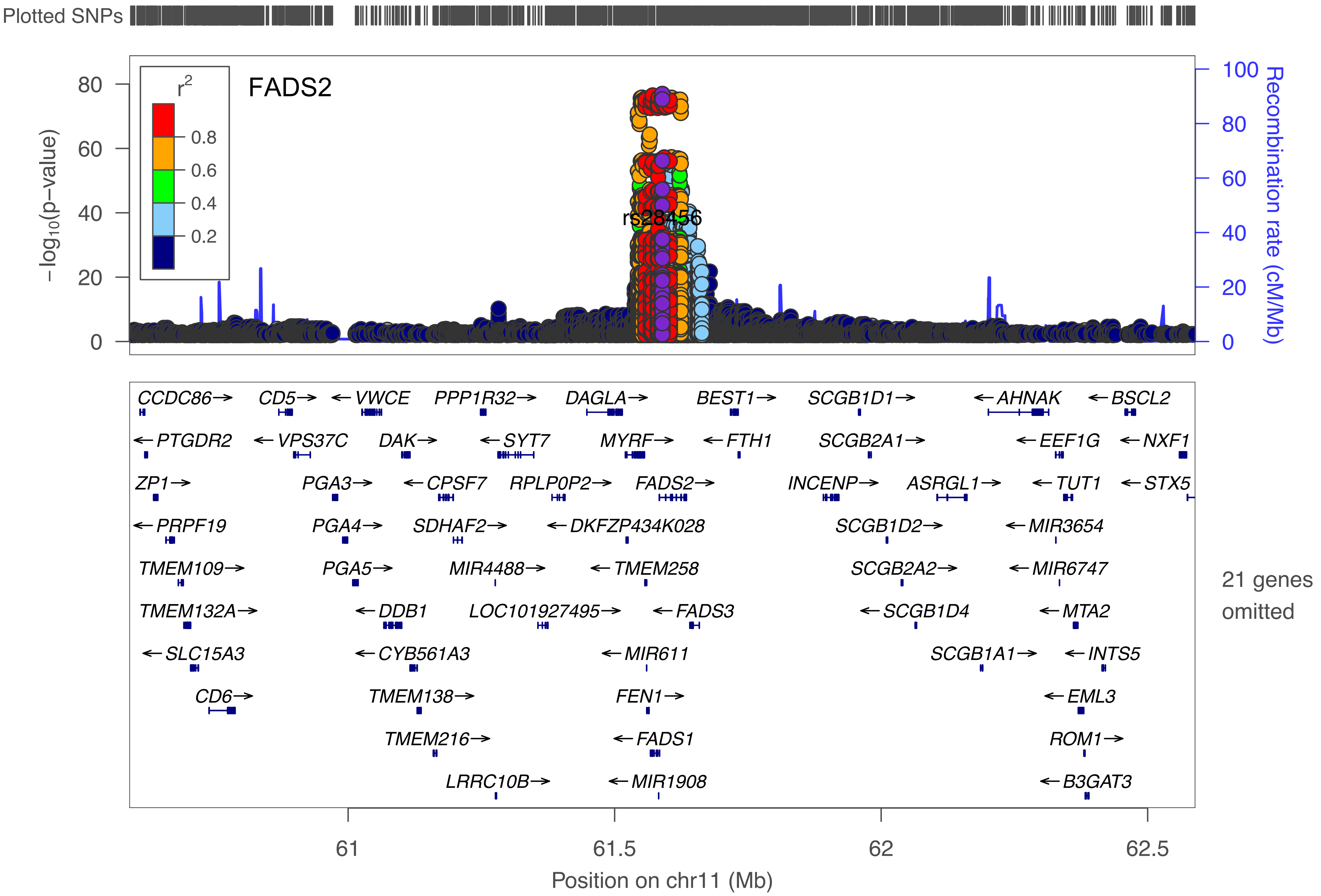

Plotted SNPs
FADS2
r^2
0.8
0.6
0.4
0.2
$-\log_{10}$(p−value)
0
20
40
60
80
Recombination rate (cM/Mb)
0
20
40
60
80
100
rs28456
CCDC86→
CD5→
←VWCE
PPP1R32→
DAGLA→
BEST1→
SCGB1D1→
←AHNAK
←BSCL2
←PTGDR2
←VPS37C
DAK→
←SYT7
MYRF→
←FTH1
SCGB2A1→
←EEF1G
←NXF1
ZP1→
PGA3→
←CPSF7
RPLP0P2→
FADS2→
INCENP→
ASRGL1→
←TUT1
←STX5
←PRPF19
PGA4→
SDHAF2→
←DKFZP434K028
SCGB1D2→
←MIR3654
TMEM109→
PGA5→
MIR4488→
←TMEM258
SCGB2A2→
←MIR6747
TMEM132A→
←DDB1
LOC101927495→
←FADS3
←SCGB1D4
←MTA2
←SLC15A3
←CYB561A3
←MIR611
SCGB1A1→
←INTS5
CD6→
TMEM138→
FEN1→
←EML3
TMEM216→
←FADS1
ROM1→
LRRC10B→
←MIR1908
←B3GAT3
21 genes omitted
61
61.5
62
62.5
Position on chr11 (Mb)

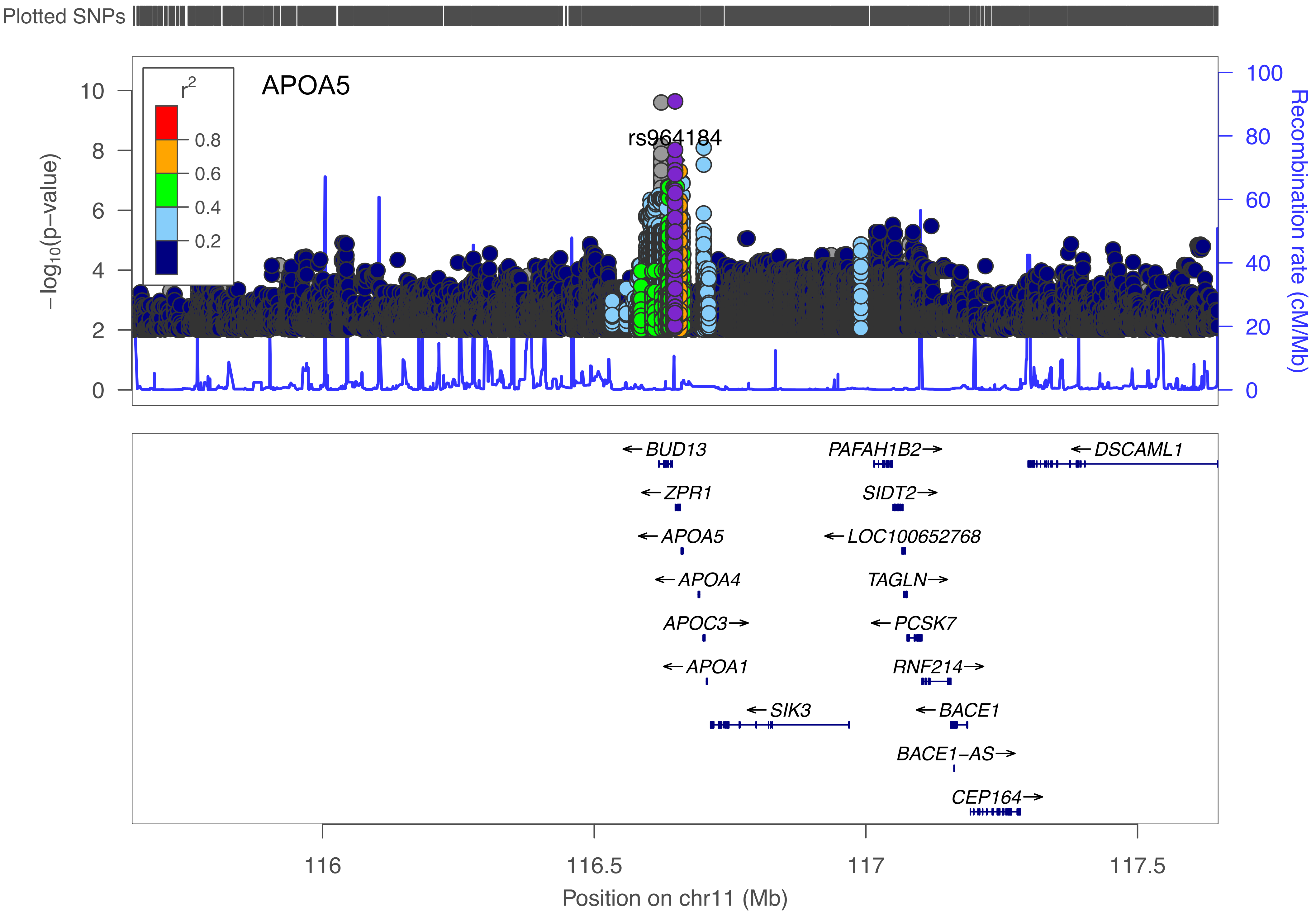

Plotted SNPs
APOA5
r2
0.8
0.6
0.4
0.2
rs964184
−log10(p−value)
Recombination rate (cM/Mb)
BUD13
ZPR1
APOA5
APOA4
APOC3
APOA1
SIK3
PAFAH1B2
SIDT2
LOC100652768
TAGLN
PCSK7
RNF214
BACE1
BACE1−AS
CEP164
DSCAML1
116
116.5
117
117.5
Position on chr11 (Mb)

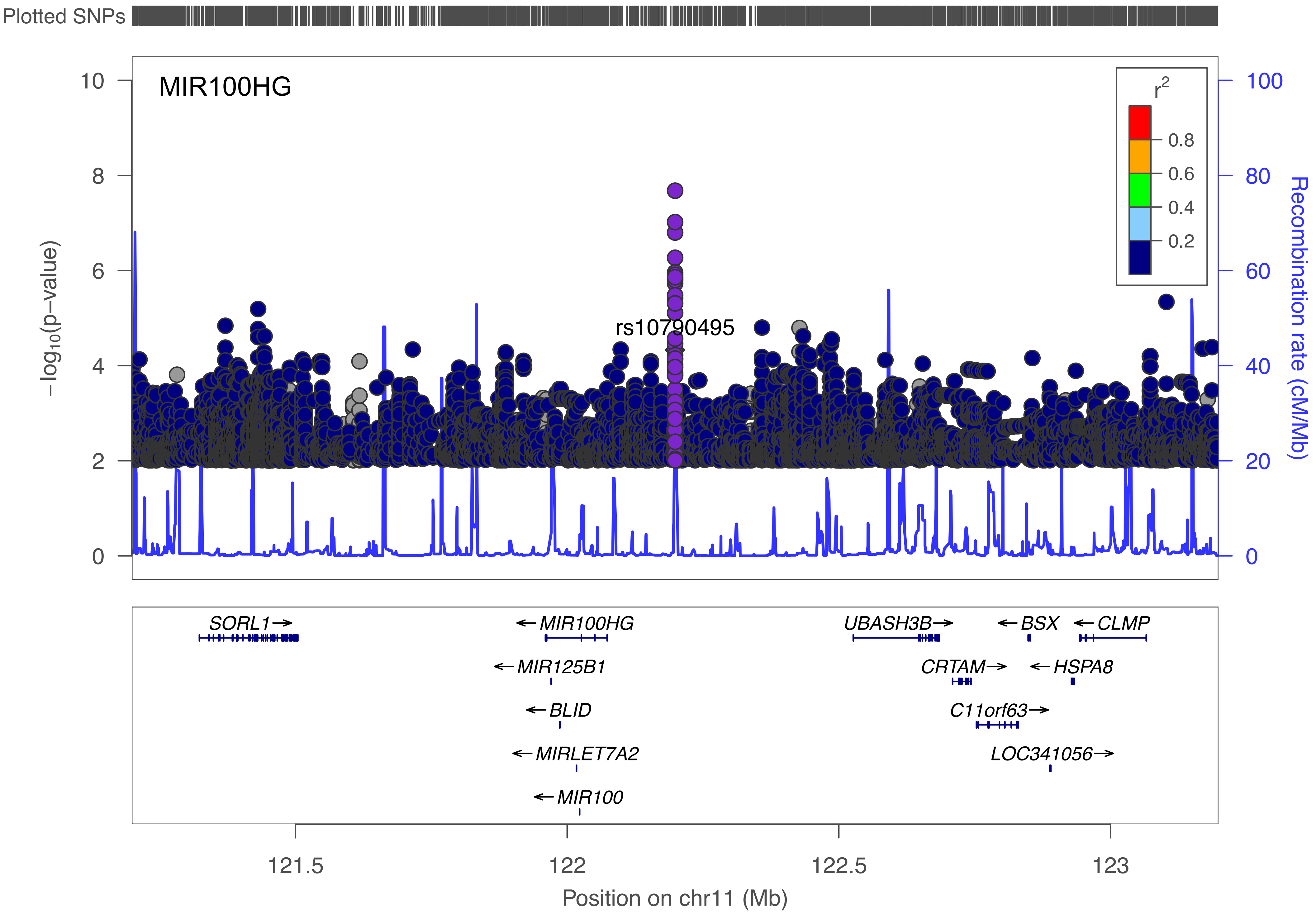

Plotted SNPs
MIR100HG
rs10790495
r2
0.8
0.6
0.4
0.2
−log10(p−value)
Recombination rate (cM/Mb)
0
2
4
6
8
10
0
20
40
60
80
100
SORL1→
←MIR100HG
←MIR125B1
←BLID
←MIRLET7A2
←MIR100
UBASH3B→
CRTAM→
C11orf63→
LOC341056→
←BSX
←HSPA8
←CLMP
121.5
122
122.5
123
Position on chr11 (Mb)

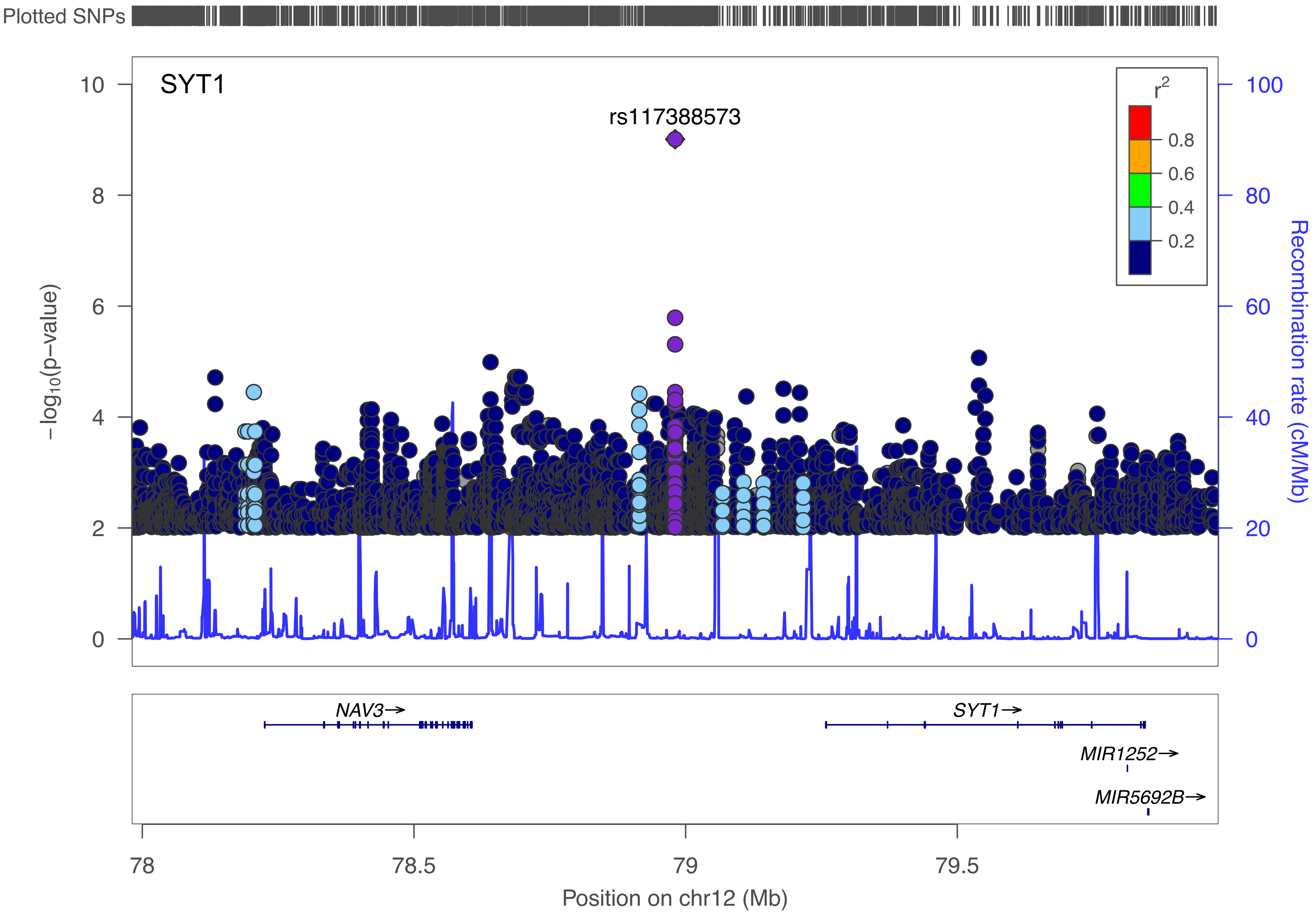

Plotted SNPs
SYT1
rs117388573
10
8
6
4
2
0
−log10(p−value)
100
80
60
40
20
0
Recombination rate (cM/Mb)
r2
0.8
0.6
0.4
0.2
NAV3→
SYT1→
MIR1252→
MIR5692B→
78
78.5
79
79.5
Position on chr12 (Mb)

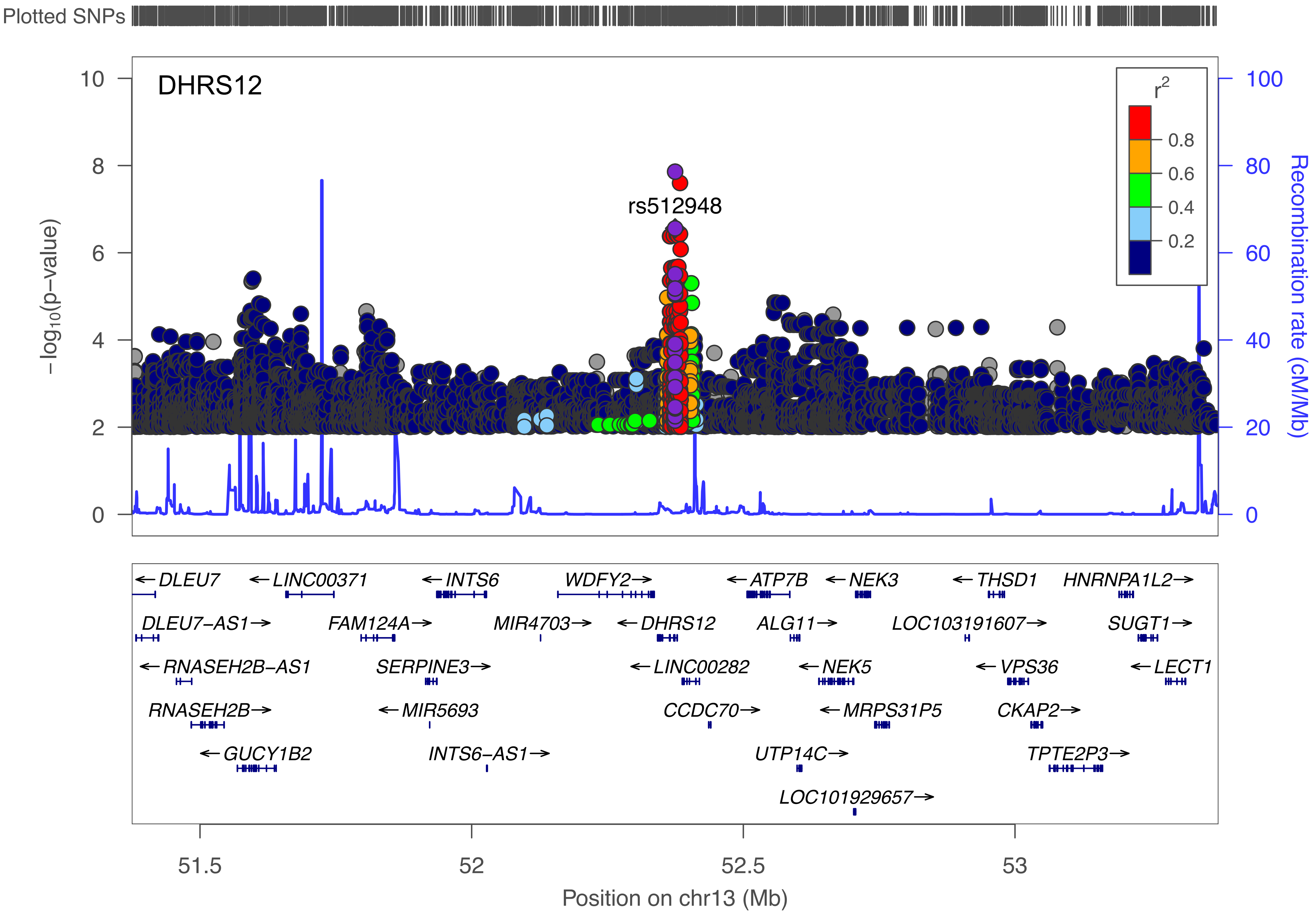

Plotted SNPs
DHRS12
rs512948
r2
0.8
0.6
0.4
0.2
−log10(p−value)
Recombination rate (cM/Mb)
0
2
4
6
8
10
20
40
60
80
100
DLEU7
LINC00371
INTS6
WDFY2
ATP7B
NEK3
THSD1
HNRNPA1L2
DLEU7−AS1
FAM124A
MIR4703
DHRS12
ALG11
LOC103191607
SUGT1
RNASEH2B−AS1
SERPINE3
LINC00282
NEK5
VPS36
LECT1
RNASEH2B
MIR5693
CCDC70
MRPS31P5
CKAP2
GUCY1B2
INTS6−AS1
UTP14C
TPTE2P3
LOC101929657
51.5
52
52.5
53
Position on chr13 (Mb)

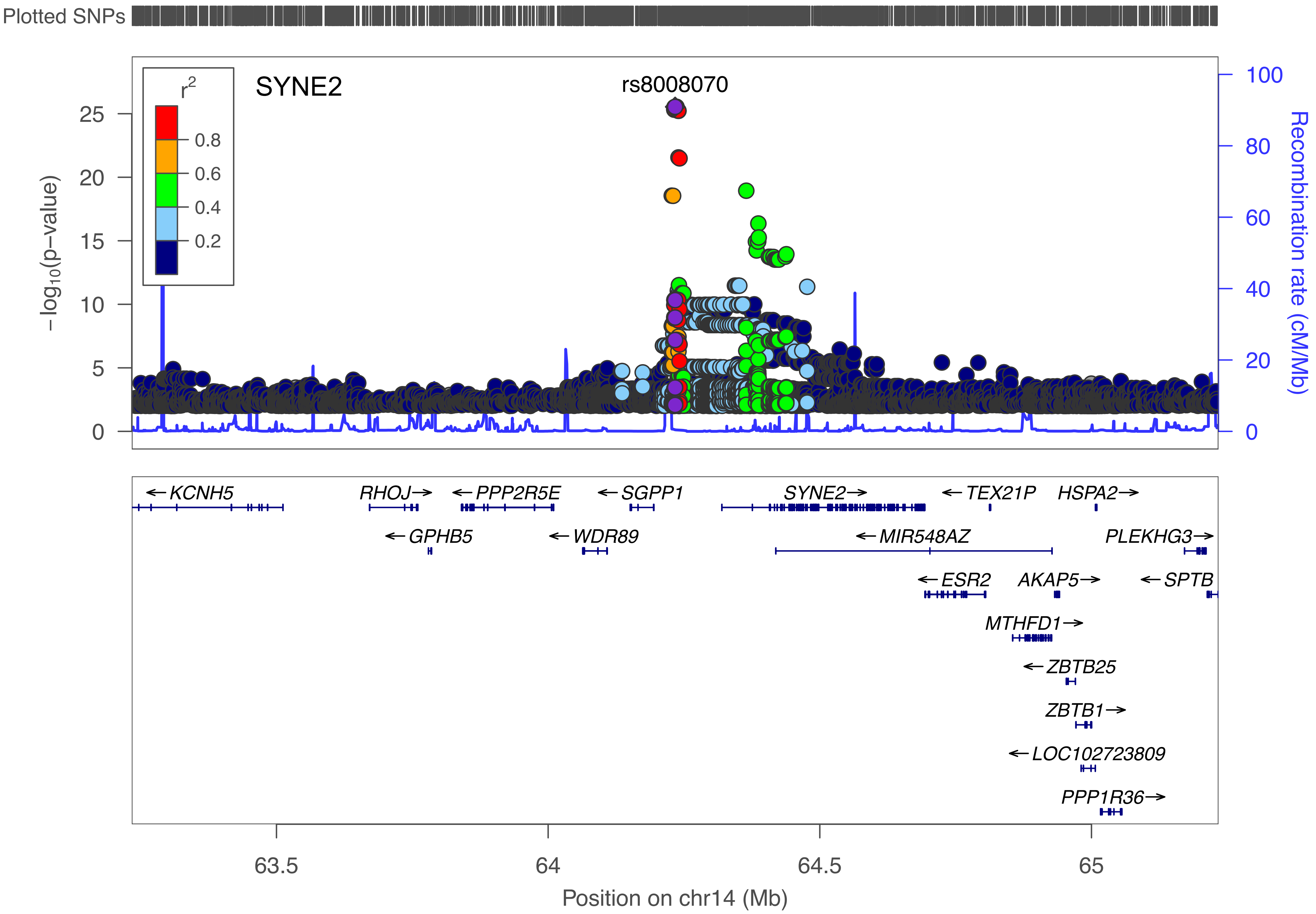

Plotted SNPs
SYNE2
rs8008070
r²
0.8
0.6
0.4
0.2
−log10(p−value)
Recombination rate (cM/Mb)
0
5
10
15
20
25
0
20
40
60
80
100
←KCNH5
RHOJ→
←PPP2R5E
←SGPP1
SYNE2→
←TEX21P
HSPA2→
←GPHB5
←WDR89
←MIR548AZ
PLEKHG3→
←ESR2
AKAP5→
←SPTB
MTHFD1→
←ZBTB25
ZBTB1→
←LOC102723809
PPP1R36→
63.5
64
64.5
65
Position on chr14 (Mb)

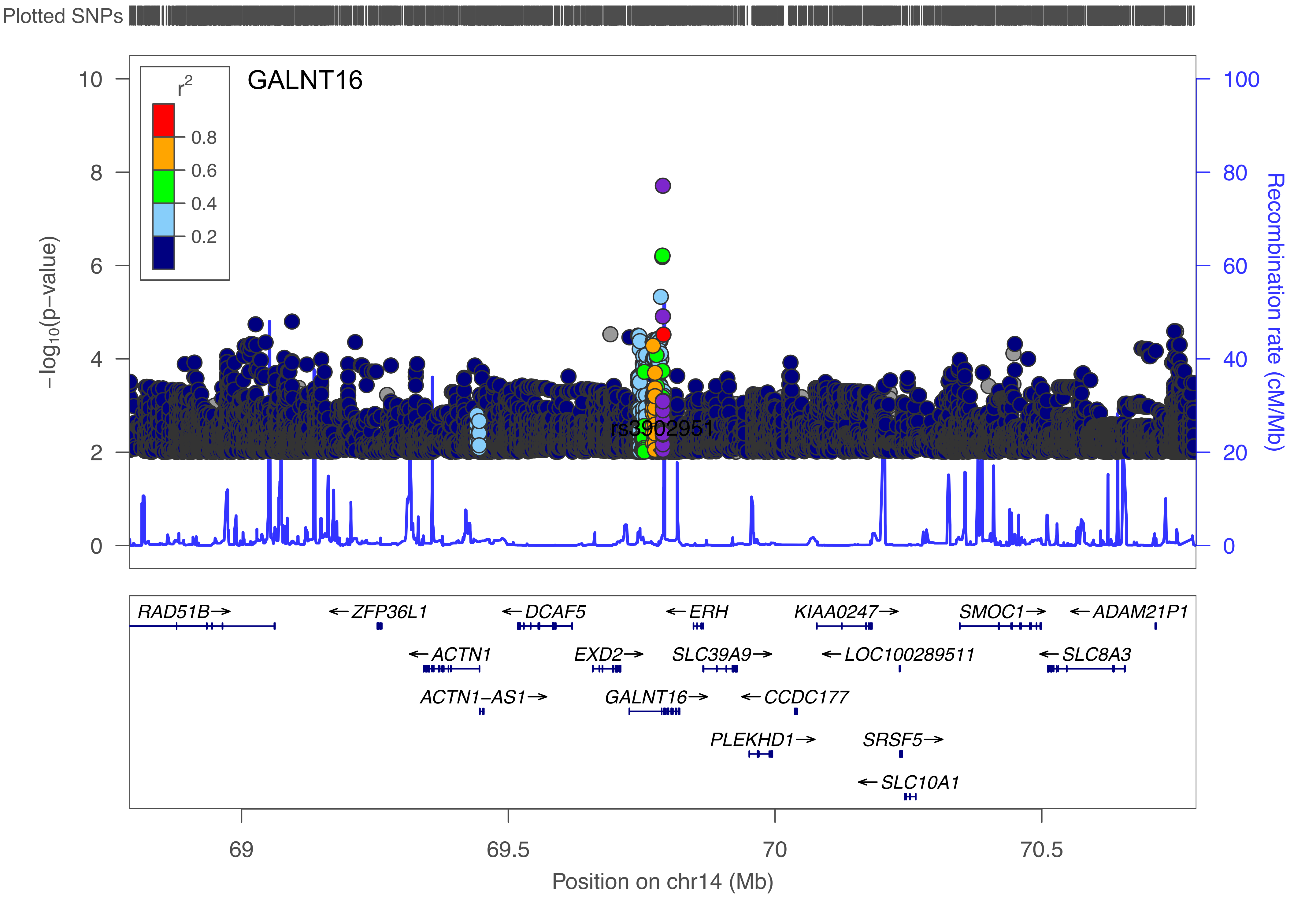
Plotted SNPs
GALNT16
r2
0.8
0.6
0.4
0.2
rs3902951
−log10(p−value)
Recombination rate (cM/Mb)
RAD51B→
←ZFP36L1
←DCAF5
←ERH
KIAA0247→
SMOC1→
←ADAM21P1
←ACTN1
EXD2→
SLC39A9→
←LOC100289511
←SLC8A3
ACTN1−AS1→
GALNT16→
←CCDC177
PLEKHD1→
SRSF5→
←SLC10A1
69
69.5
70
70.5
Position on chr14 (Mb)

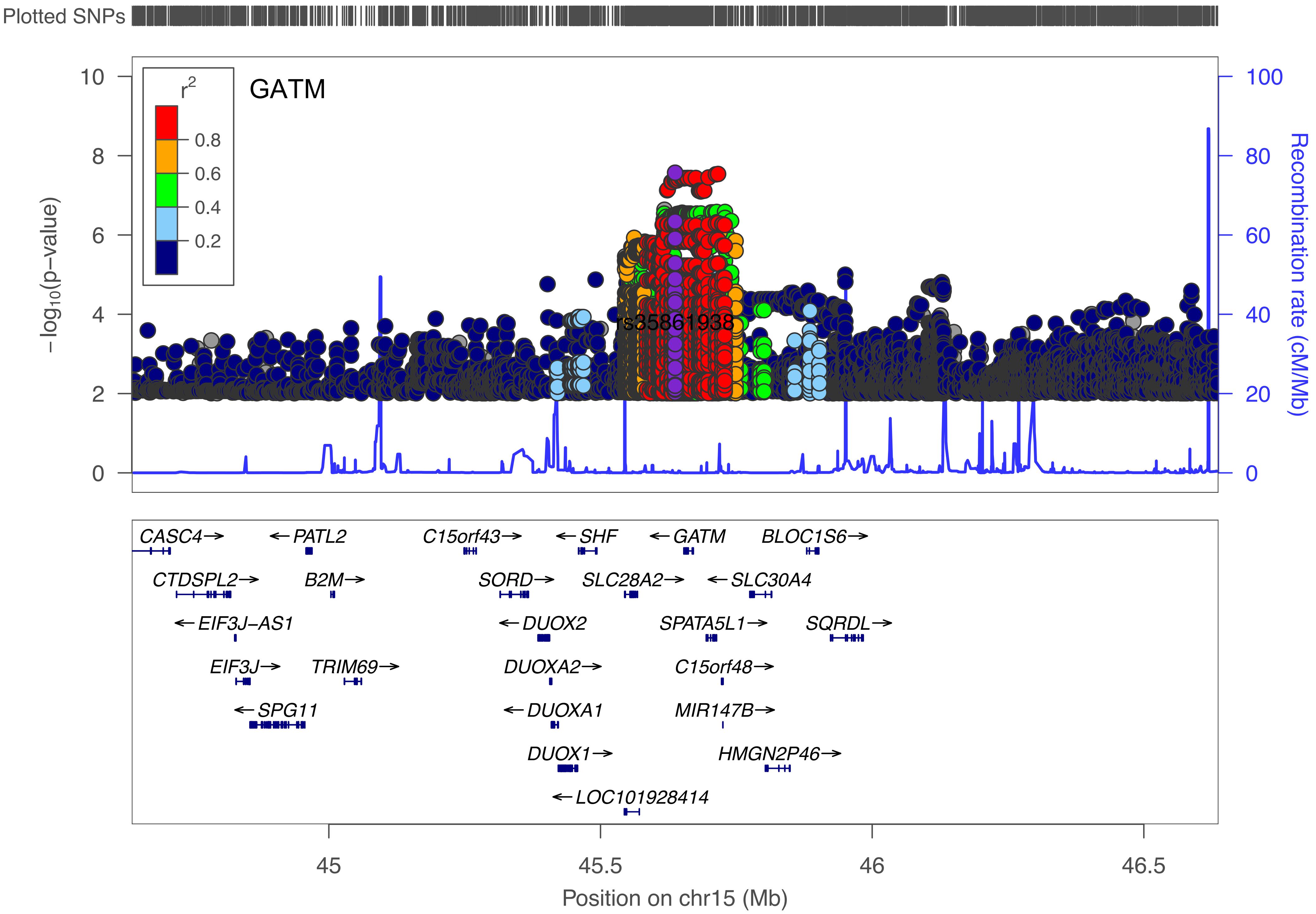

Plotted SNPs
GATM
r^2
0.8
0.6
0.4
0.2
rs35861938
$-\log_{10}$(p-value)
Recombination rate (cM/Mb)
10
8
6
4
2
0
100
80
60
40
20
0
CASC4→
←PATL2
C15orf43→
←SHF
←GATM
BLOC1S6→
CTDSPL2→
B2M→
SORD→
SLC28A2→
←SLC30A4
←EIF3J-AS1
←DUOX2
SPATA5L1→
SQRDL→
EIF3J→
TRIM69→
DUOXA2→
C15orf48→
←SPG11
←DUOXA1
MIR147B→
DUOX1→
HMGN2P46→
←LOC101928414
45
45.5
46
46.5
Position on chr15 (Mb)

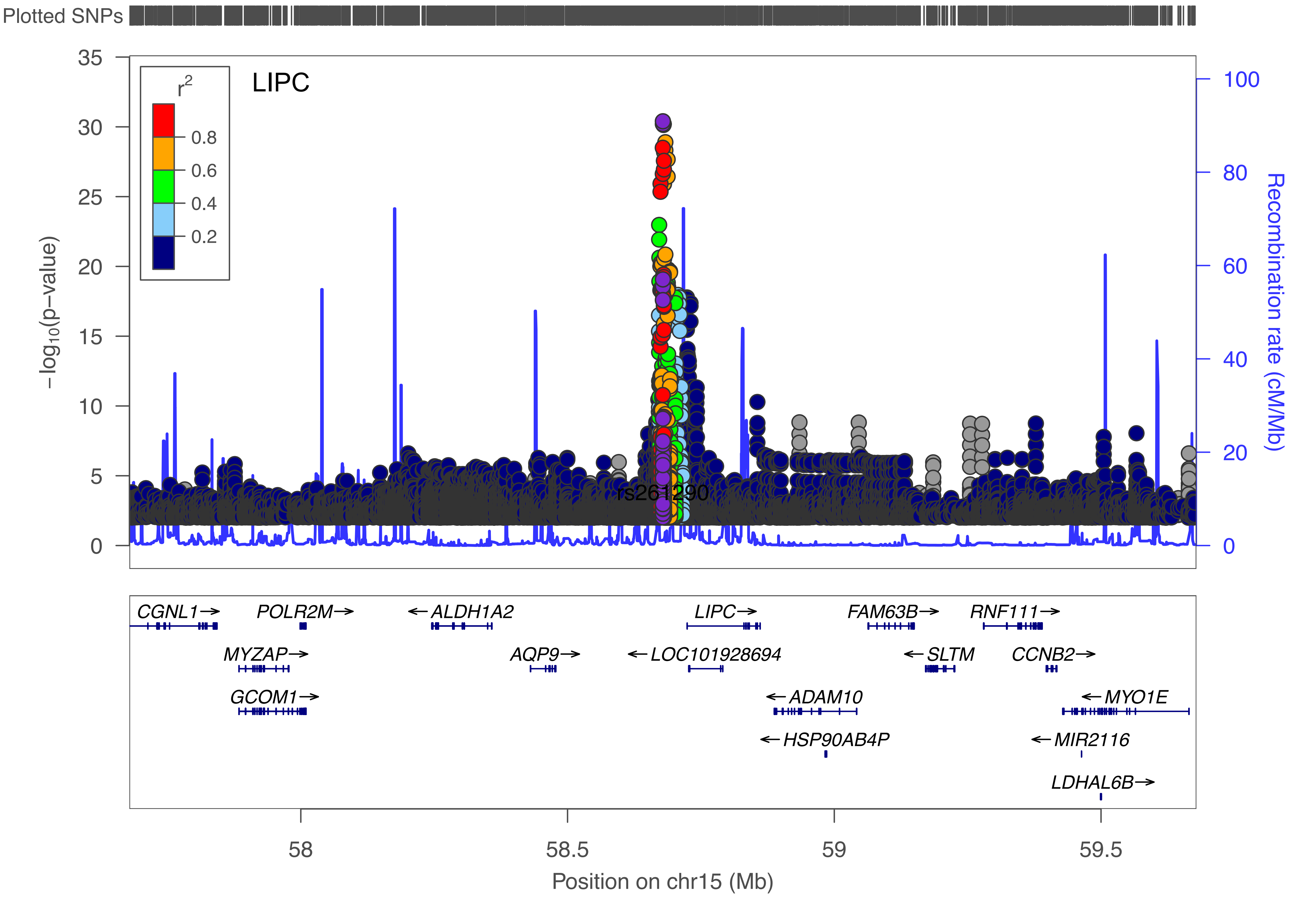

Plotted SNPs
LIPC
r2
0.8
0.6
0.4
0.2
rs261290
−log10(p−value)
Recombination rate (cM/Mb)
35
30
25
20
15
10
5
0
100
80
60
40
20
0
CGNL1→
POLR2M→
←ALDH1A2
LIPC→
FAM63B→
RNF111→
MYZAP→
AQP9→
←LOC101928694
←SLTM
CCNB2→
GCOM1→
←ADAM10
←MYO1E
←HSP90AB4P
←MIR2116
LDHAL6B→
58
58.5
59
59.5
Position on chr15 (Mb)

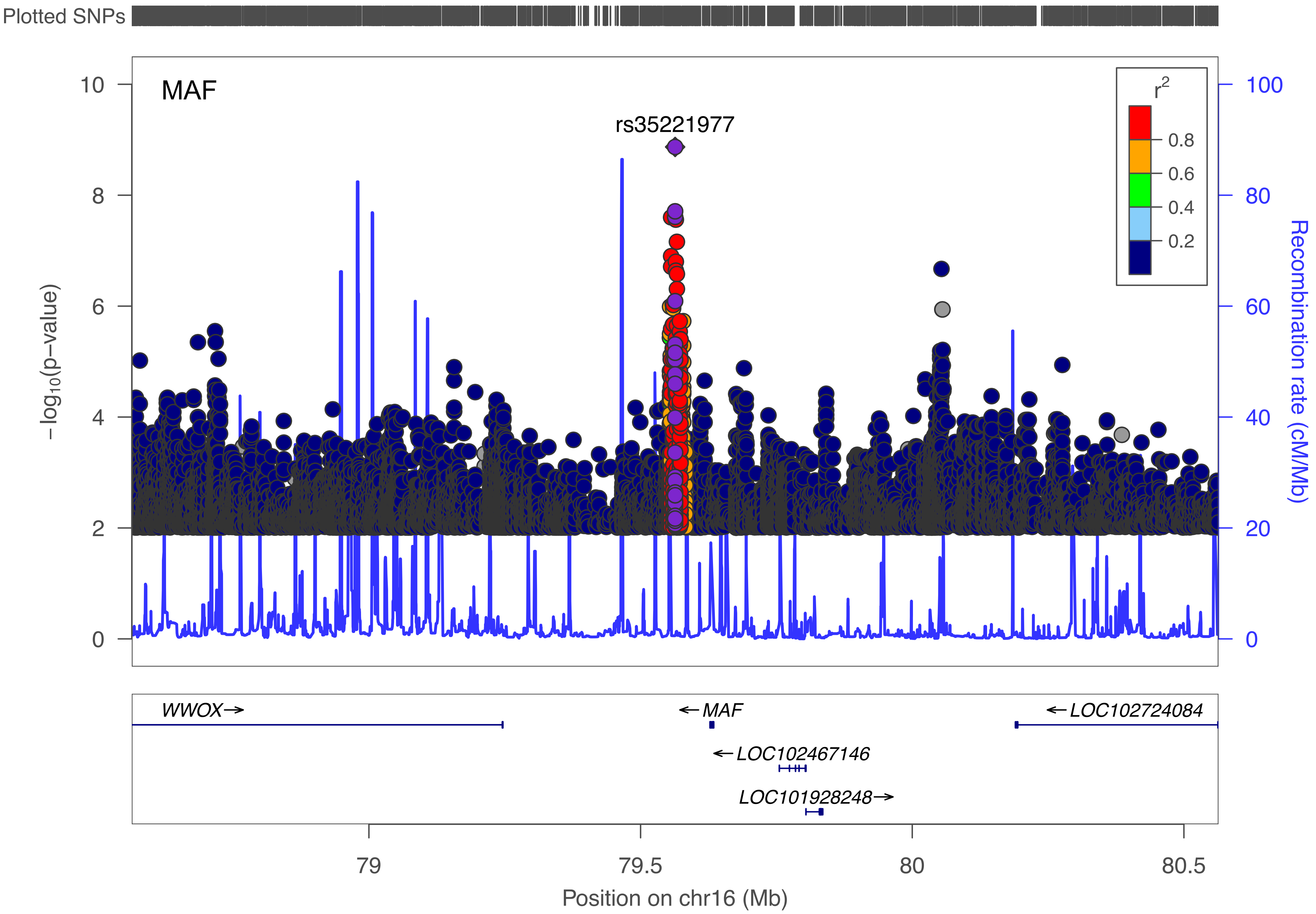

Plotted SNPs
MAF
rs35221977
r2
0.8
0.6
0.4
0.2
−log10(p−value)
Recombination rate (cM/Mb)
10
8
6
4
2
0
100
80
60
40
20
0
WWOX→
←MAF
←LOC102724084
←LOC102467146
LOC101928248→
79
79.5
80
80.5
Position on chr16 (Mb)

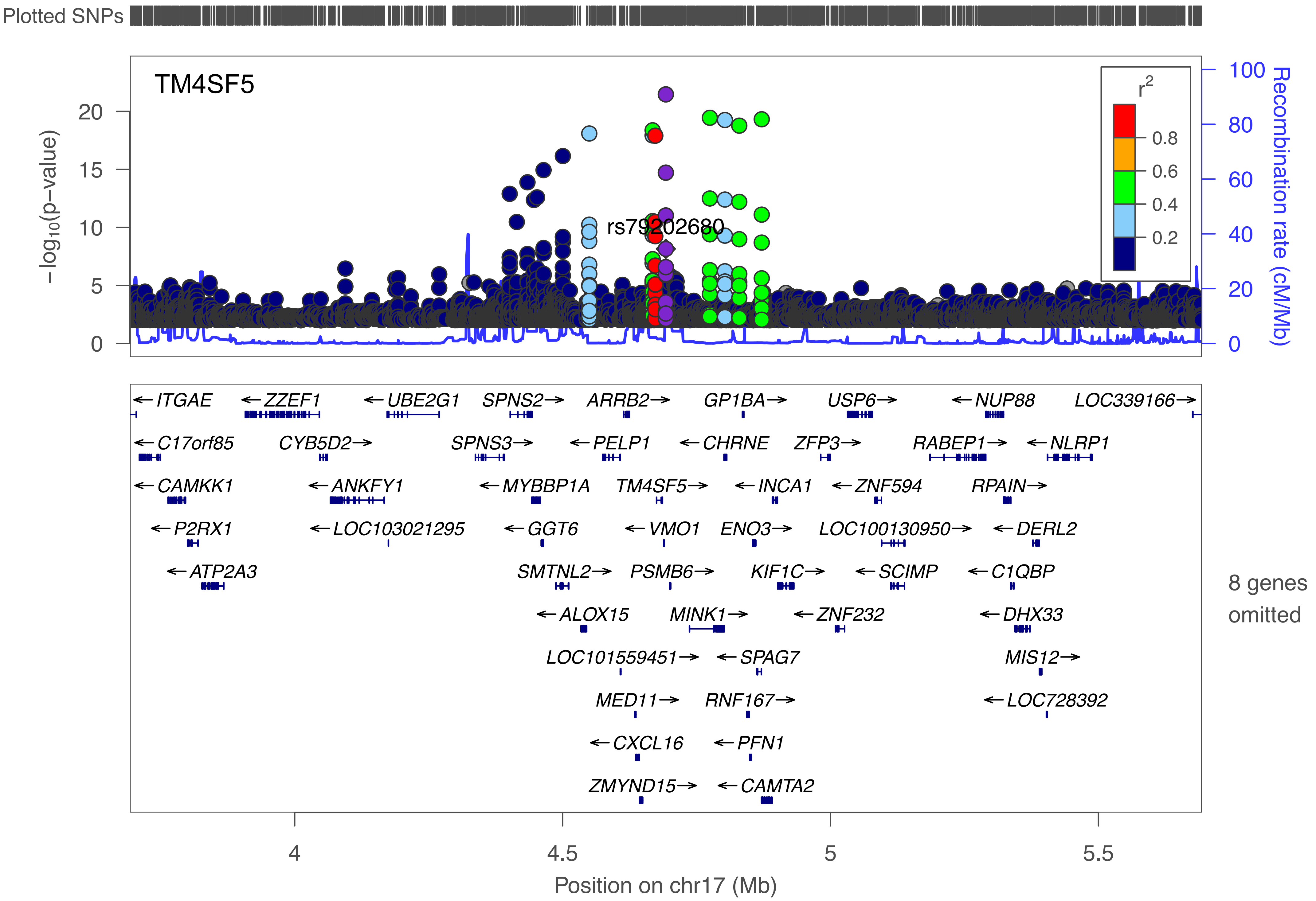

Plotted SNPs
TM4SF5
rs79202680
−log10(p−value)
Recombination rate (cM/Mb)
r2
0.8
0.6
0.4
0.2
ITGAE
ZZEF1
UBE2G1
SPNS2
ARRB2
GP1BA
USP6
NUP88
LOC339166
C17orf85
CYB5D2
SPNS3
PELP1
CHRNE
ZFP3
RABEP1
NLRP1
CAMKK1
ANKFY1
MYBBP1A
TM4SF5
INCA1
ZNF594
RPAIN
P2RX1
LOC103021295
GGT6
VMO1
ENO3
LOC100130950
DERL2
ATP2A3
SMTNL2
PSMB6
KIF1C
SCIMP
C1QBP
ALOX15
MINK1
ZNF232
DHX33
LOC101559451
SPAG7
MIS12
MED11
RNF167
LOC728392
CXCL16
PFN1
ZMYND15
CAMTA2
8 genes omitted
Position on chr17 (Mb)

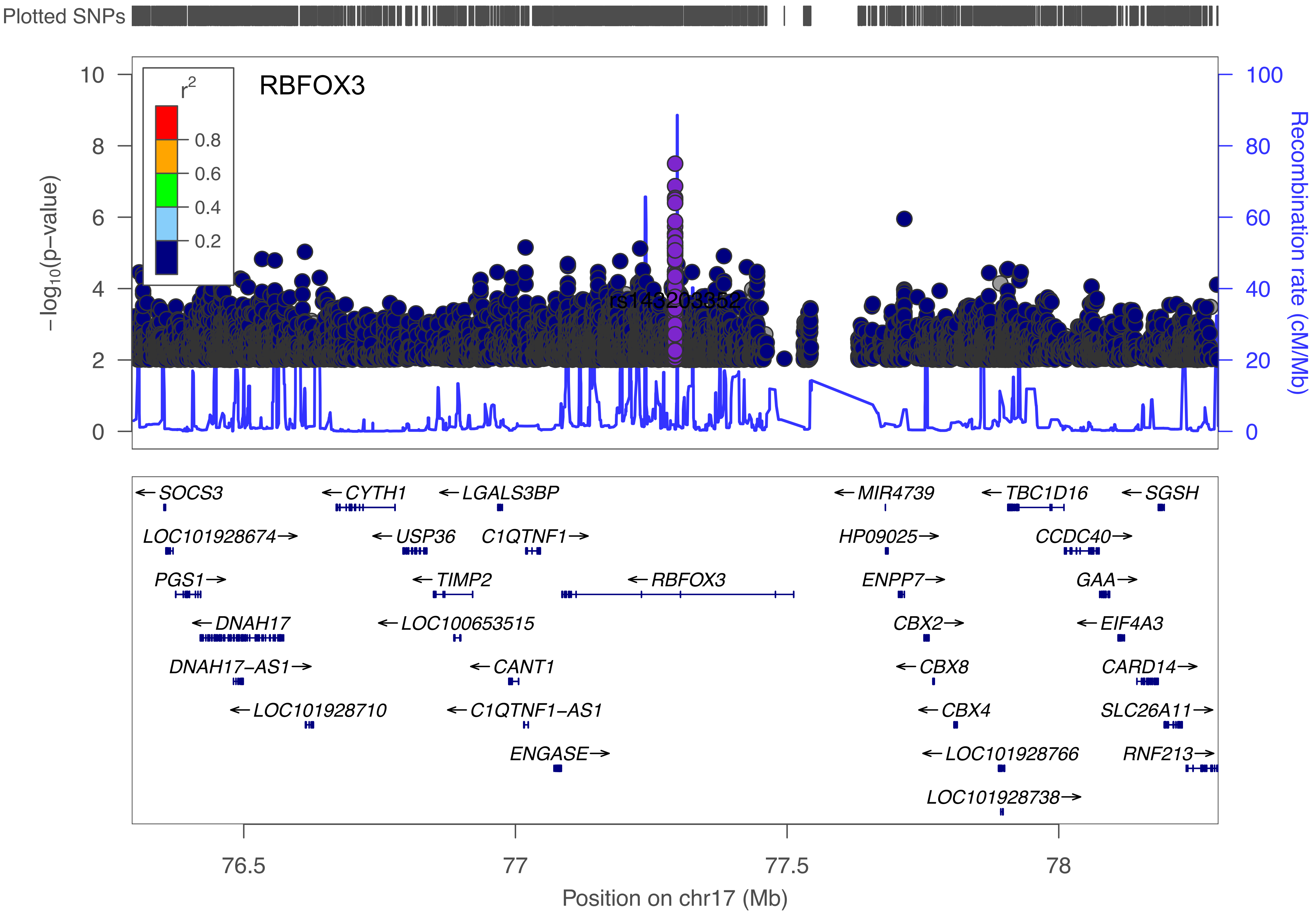

Plotted SNPs
RBFOX3
r2
0.8
0.6
0.4
0.2
rs143203352
−log10(p−value)
Recombination rate (cM/Mb)
SOCS3
CYTH1
LGALS3BP
MIR4739
TBC1D16
SGSH
LOC101928674
USP36
C1QTNF1
HP09025
CCDC40
PGS1
TIMP2
RBFOX3
ENPP7
GAA
DNAH17
LOC100653515
CBX2
EIF4A3
DNAH17−AS1
CANT1
CBX8
CARD14
LOC101928710
C1QTNF1−AS1
CBX4
SLC26A11
ENGASE
LOC101928766
RNF213
LOC101928738
76.5
77
77.5
78
Position on chr17 (Mb)

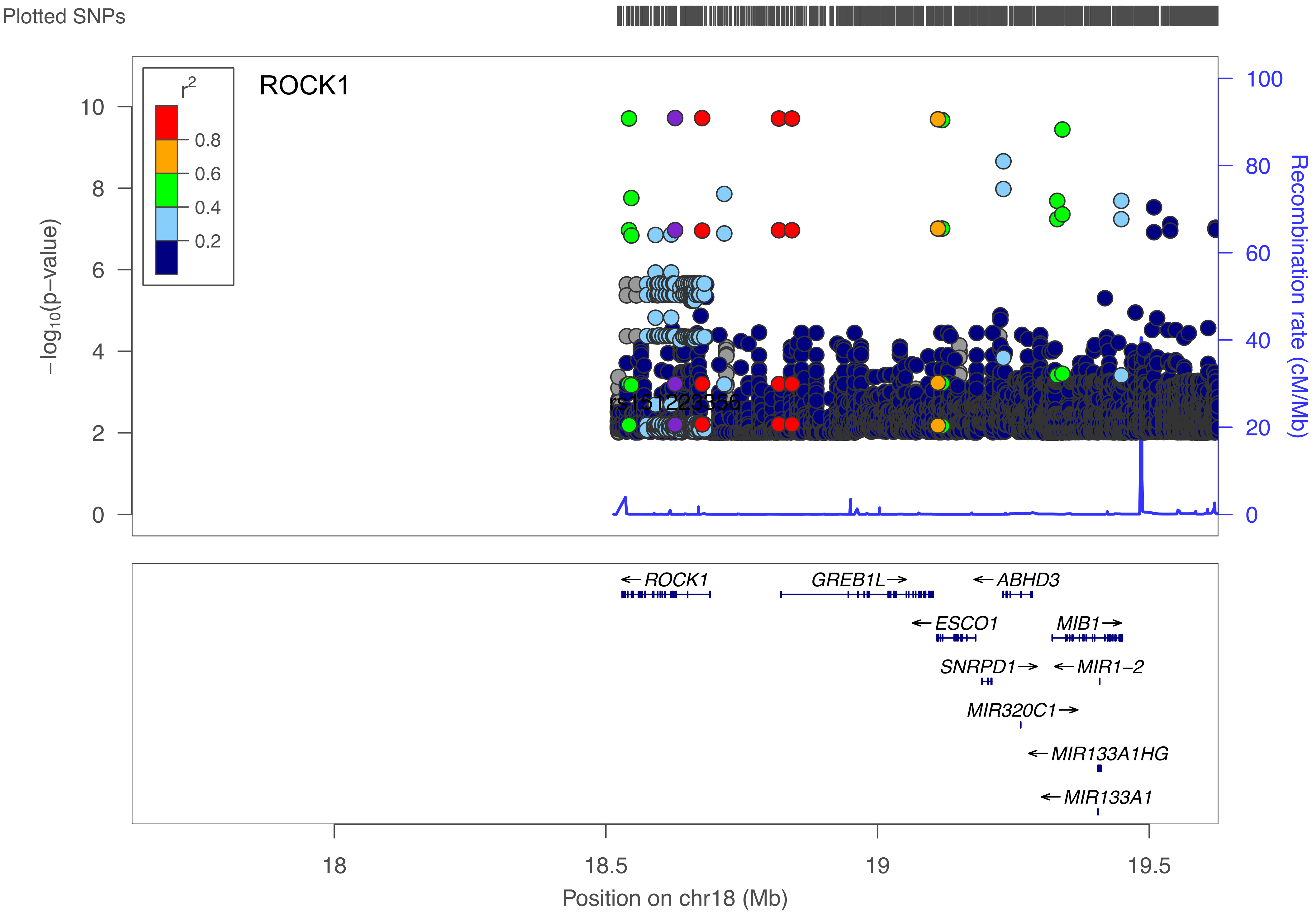

Plotted SNPs
ROCK1
r^2
0.8
0.6
0.4
0.2
10
8
6
4
2
0
−log10(p−value)
100
80
60
40
20
0
Recombination rate (cM/Mb)
rs151223356
←ROCK1
GREB1L→
←ABHD3
←ESCO1
MIB1→
SNRPD1→
←MIR1−2
MIR320C1→
←MIR133A1HG
←MIR133A1
18
18.5
19
19.5
Position on chr18 (Mb)

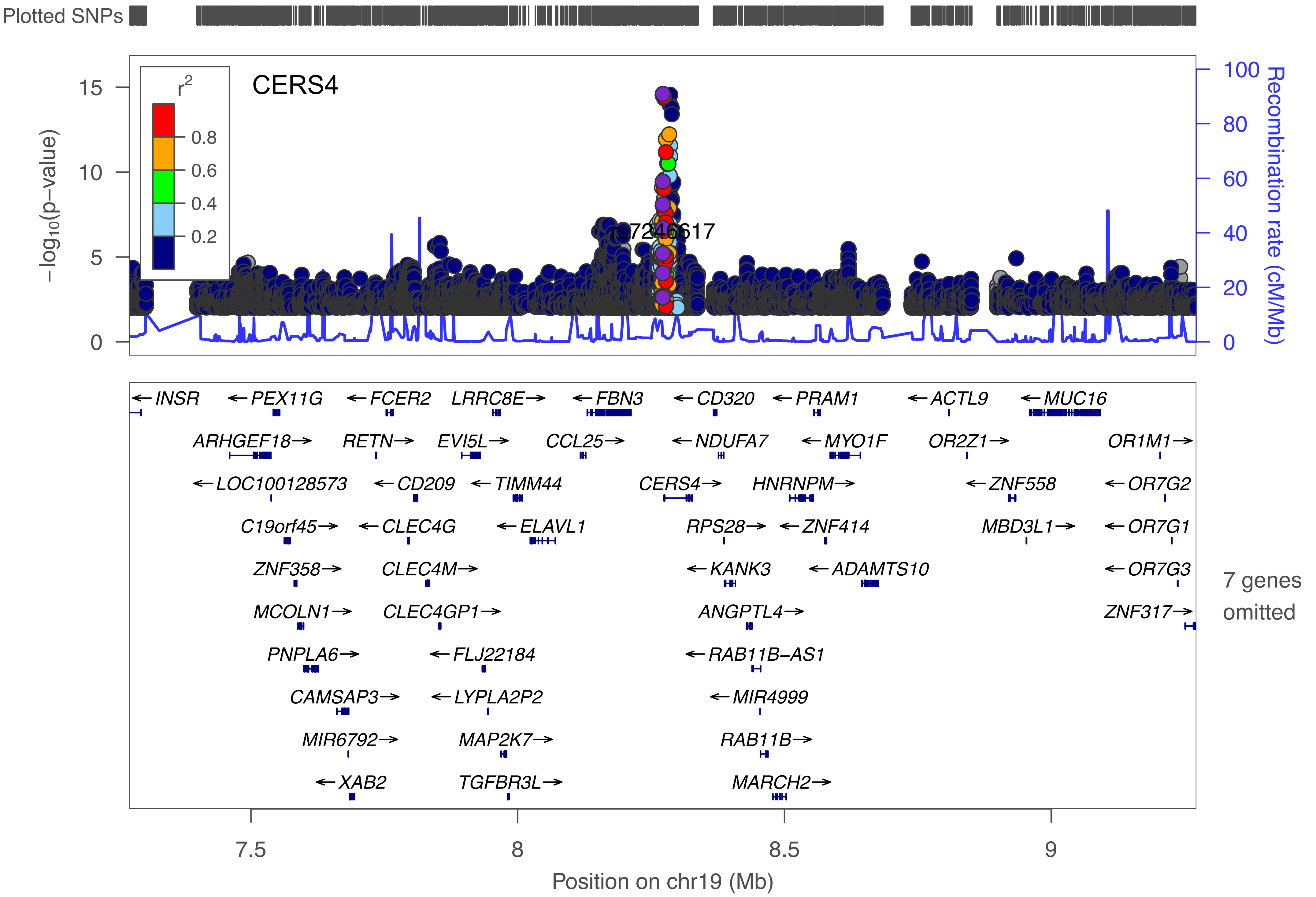
Plotted SNPs
CERS4
r²
0.8
0.6
0.4
0.2
rs7246617
−log10(p−value)
Recombination rate (cM/Mb)
INSR
PEX11G
FCER2
LRRC8E
FBN3
CD320
PRAM1
ACTL9
MUC16
ARHGEF18
RETN
EVI5L
CCL25
NDUFA7
MYO1F
OR2Z1
OR1M1
LOC100128573
CD209
TIMM44
CERS4
HNRNPM
ZNF558
OR7G2
C19orf45
CLEC4G
ELAVL1
RPS28
ZNF414
MBD3L1
OR7G1
ZNF358
CLEC4M
KANK3
ADAMTS10
OR7G3
MCOLN1
CLEC4GP1
ANGPTL4
ZNF317
PNPLA6
FLJ22184
RAB11B-AS1
CAMSAP3
LYPLA2P2
MIR4999
MIR6792
MAP2K7
RAB11B
XAB2
TGFBR3L
MARCH2
7 genes omitted
Position on chr19 (Mb)

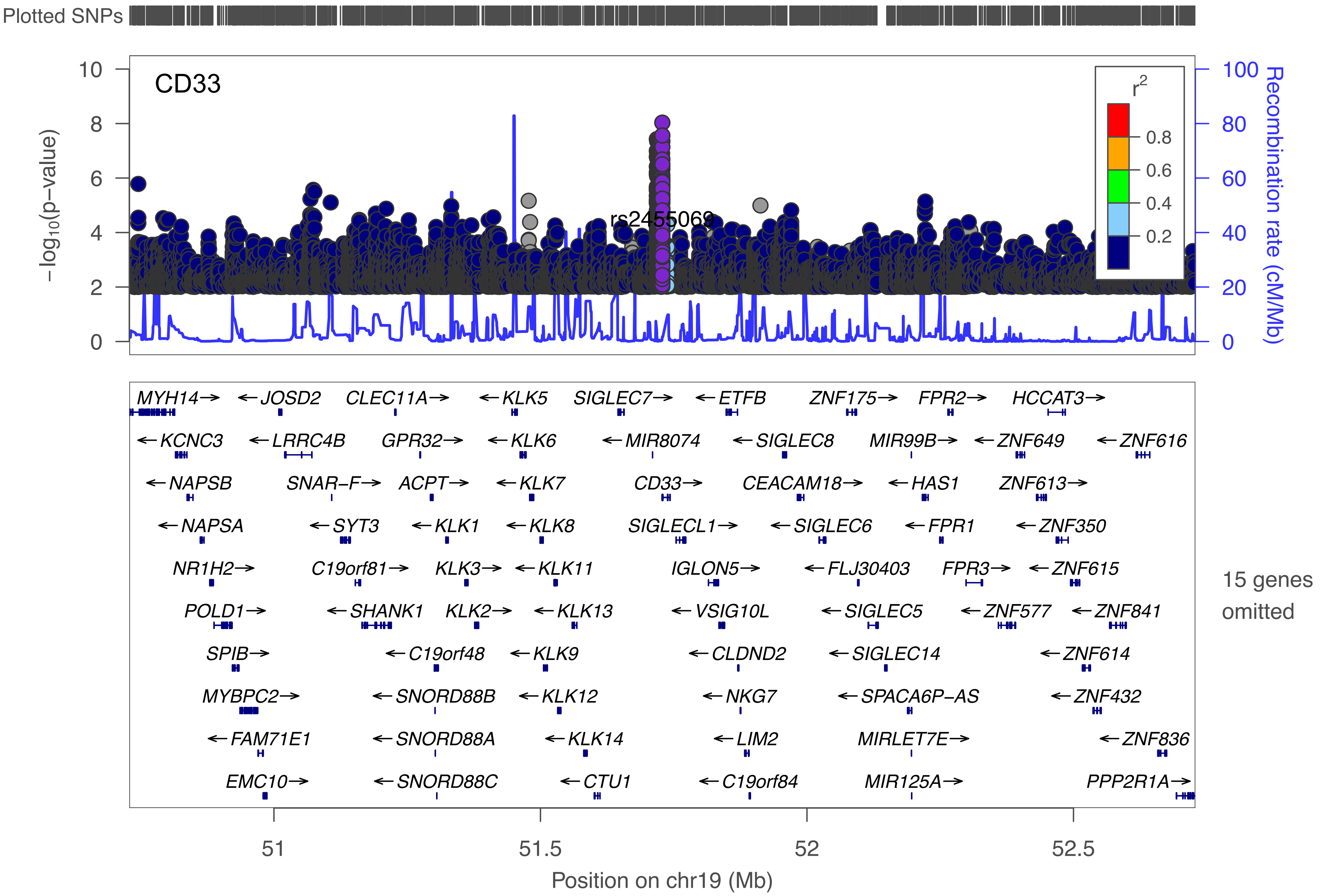

Plotted SNPs
CD33
rs2455069
−log10(p−value)
Recombination rate (cM/Mb)
r2
0.8
0.6
0.4
0.2
10
8
6
4
2
0
100
80
60
40
20
MYH14→ ←JOSD2 CLEC11A→ ←KLK5 SIGLEC7→ ←ETFB ZNF175→ FPR2→ HCCAT3→
←KCNC3 ←LRRC4B GPR32→ ←KLK6 ←MIR8074 ←SIGLEC8 MIR99B→ ←ZNF649 ←ZNF616
←NAPSB SNAR−F→ ACPT→ ←KLK7 CD33→ CEACAM18→ ←HAS1 ZNF613→
←NAPSA ←SYT3 ←KLK1 ←KLK8 SIGLECL1→ ←SIGLEC6 ←FPR1 ←ZNF350
NR1H2→ C19orf81→ KLK3→ ←KLK11 IGLON5→ ←FLJ30403 FPR3→ ←ZNF615
POLD1→ ←SHANK1 KLK2→ ←KLK13 ←VSIG10L ←SIGLEC5 ←ZNF577 ←ZNF841
SPIB→ ←C19orf48 ←KLK9 ←CLDND2 ←SIGLEC14 ←ZNF614
MYBPC2→ ←SNORD88B ←KLK12 ←NKG7 ←SPACA6P−AS ←ZNF432
←FAM71E1 ←SNORD88A ←KLK14 ←LIM2 MIRLET7E→ ←ZNF836
EMC10→ ←SNORD88C ←CTU1 ←C19orf84 MIR125A→ PPP2R1A→
15 genes omitted
51
51.5
52
52.5
Position on chr19 (Mb)

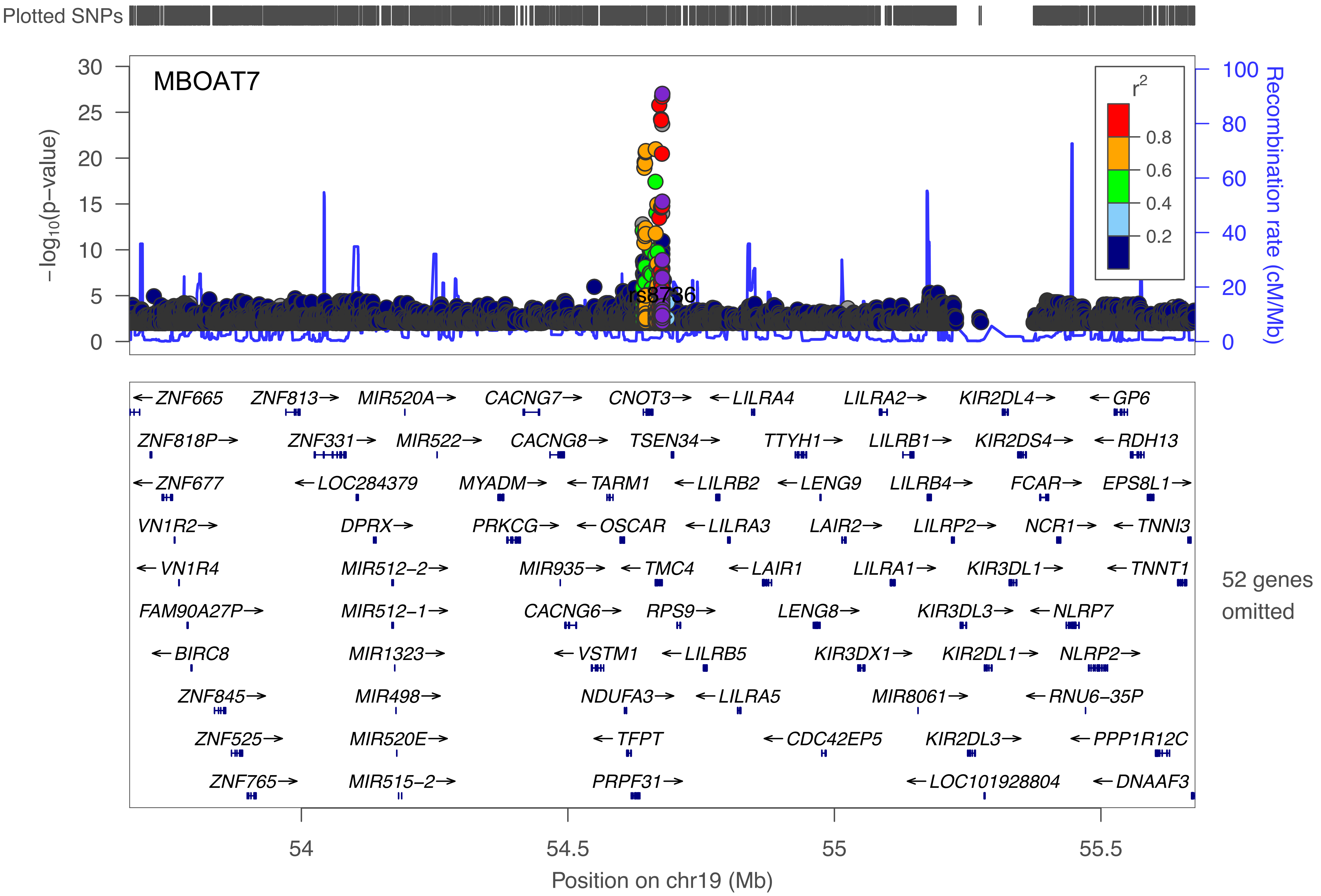

Plotted SNPs
MBOAT7
r2
0.8
0.6
0.4
0.2
rs8736
−log10(p−value)
Recombination rate (cM/Mb)
0
5
10
15
20
25
30
0
20
40
60
80
100
←ZNF665
ZNF813→
MIR520A→
CACNG7→
CNOT3→
←LILRA4
LILRA2→
KIR2DL4→
←GP6
ZNF818P→
ZNF331→
MIR522→
CACNG8→
TSEN34→
TTYH1→
LILRB1→
KIR2DS4→
←RDH13
←ZNF677
←LOC284379
MYADM→
←TARM1
←LILRB2
←LENG9
LILRB4→
FCAR→
EPS8L1→
VN1R2→
DPRX→
PRKCG→
←OSCAR
←LILRA3
LAIR2→
LILRP2→
NCR1→
←TNNI3
←VN1R4
MIR512−2→
MIR935→
←TMC4
←LAIR1
LILRA1→
KIR3DL1→
←TNNT1
FAM90A27P→
MIR512−1→
CACNG6→
RPS9→
LENG8→
KIR3DL3→
←NLRP7
←BIRC8
MIR1323→
←VSTM1
←LILRB5
KIR3DX1→
KIR2DL1→
NLRP2→
ZNF845→
MIR498→
NDUFA3→
←LILRA5
MIR8061→
←RNU6−35P
ZNF525→
MIR520E→
←TFPT
←CDC42EP5
KIR2DL3→
←PPP1R12C
ZNF765→
MIR515−2→
PRPF31→
←LOC101928804
←DNAAF3
52 genes omitted
54
54.5
55
55.5
Position on chr19 (Mb)

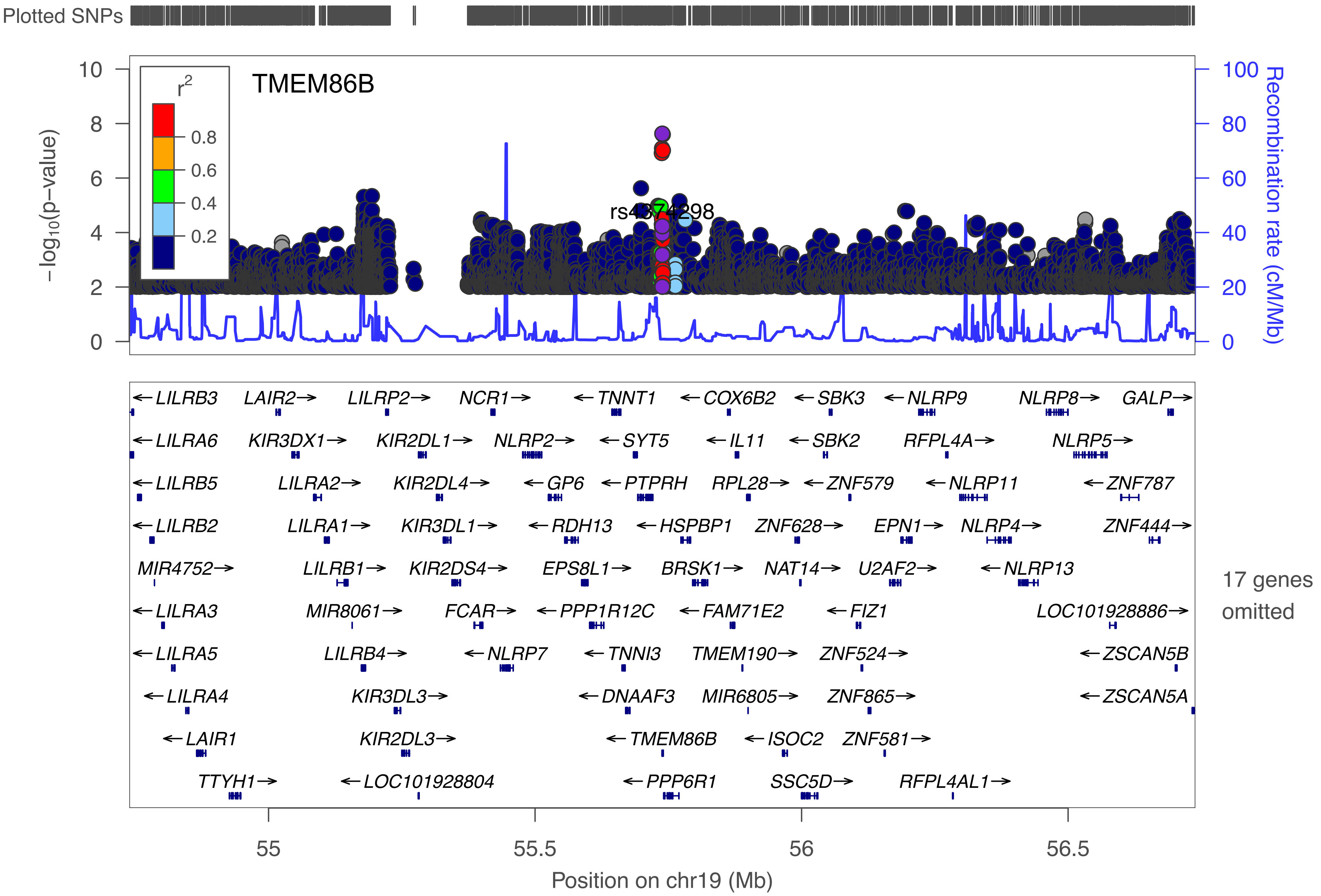

Plotted SNPs
TMEM86B
r2
0.8
0.6
0.4
0.2
rs4374298
−log10(p−value)
Recombination rate (cM/Mb)
0
2
4
6
8
10
0
20
40
60
80
100
LILRB3
LAIR2
LILRP2
NCR1
TNNT1
COX6B2
SBK3
NLRP9
NLRP8
GALP
LILRA6
KIR3DX1
KIR2DL1
NLRP2
SYT5
IL11
SBK2
RFPL4A
NLRP5
LILRB5
LILRA2
KIR2DL4
GP6
PTPRH
RPL28
ZNF579
NLRP11
ZNF787
LILRB2
LILRA1
KIR3DL1
RDH13
HSPBP1
ZNF628
EPN1
NLRP4
ZNF444
MIR4752
LILRB1
KIR2DS4
EPS8L1
BRSK1
NAT14
U2AF2
NLRP13
LILRA3
MIR8061
FCAR
PPP1R12C
FAM71E2
FIZ1
LOC101928886
LILRA5
LILRB4
NLRP7
TNNI3
TMEM190
ZNF524
ZSCAN5B
LILRA4
KIR3DL3
DNAAF3
MIR6805
ZNF865
ZSCAN5A
LAIR1
KIR2DL3
TMEM86B
ISOC2
ZNF581
TTYH1
LOC101928804
PPP6R1
SSC5D
RFPL4AL1
17 genes omitted
55
55.5
56
56.5
Position on chr19 (Mb)

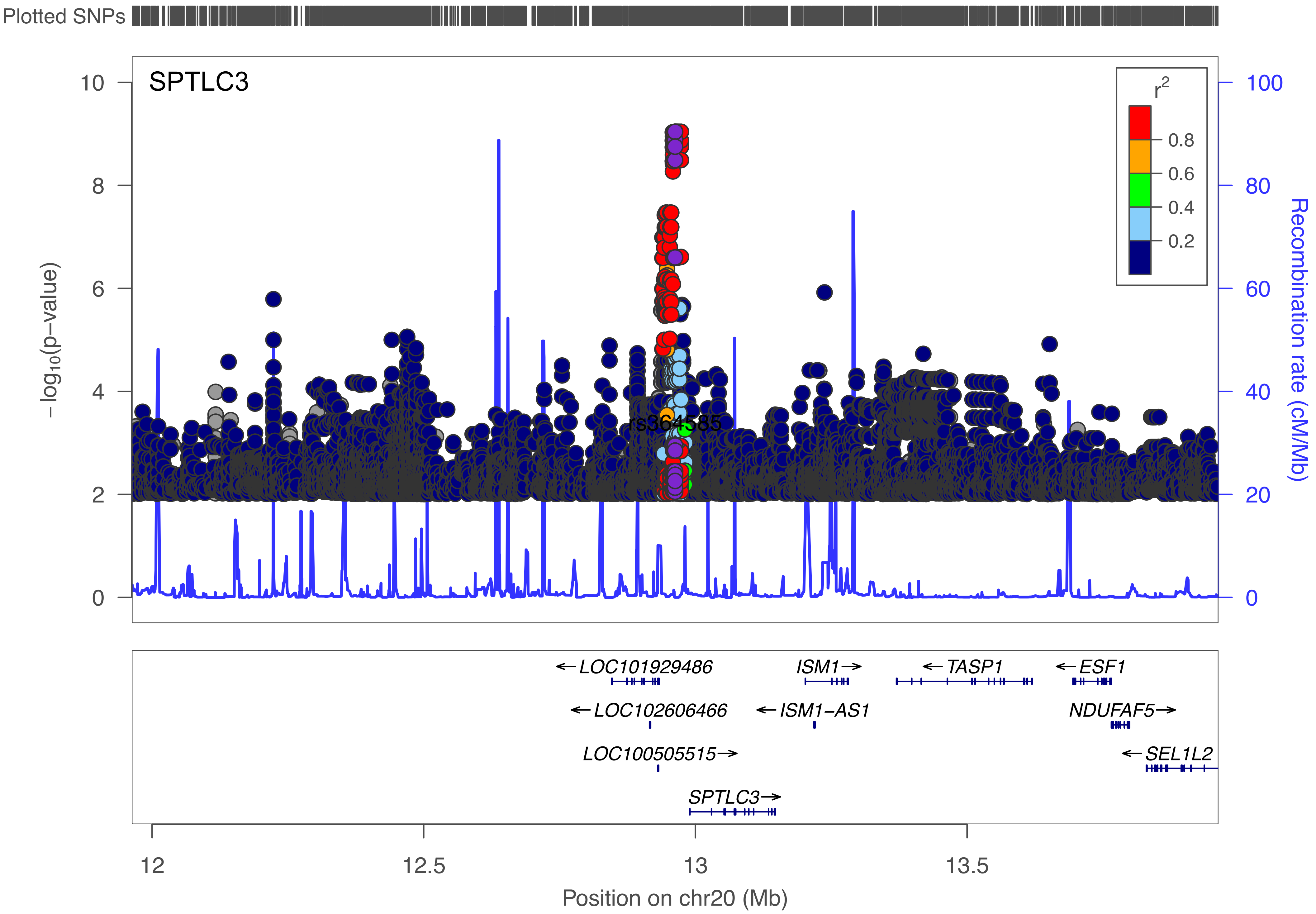
Plotted SNPs
SPTLC3
r^2
0.8
0.6
0.4
0.2
rs364585
$-\log_{10}$(p-value)
Recombination rate (cM/Mb)
10
8
6
4
2
0
100
80
60
40
20
0
← LOC101929486
ISM1 →
← TASP1
← ESF1
← LOC102606466
← ISM1−AS1
NDUFAF5 →
LOC100505515 →
← SEL1L2
SPTLC3 →
12
12.5
13
13.5
Position on chr20 (Mb)

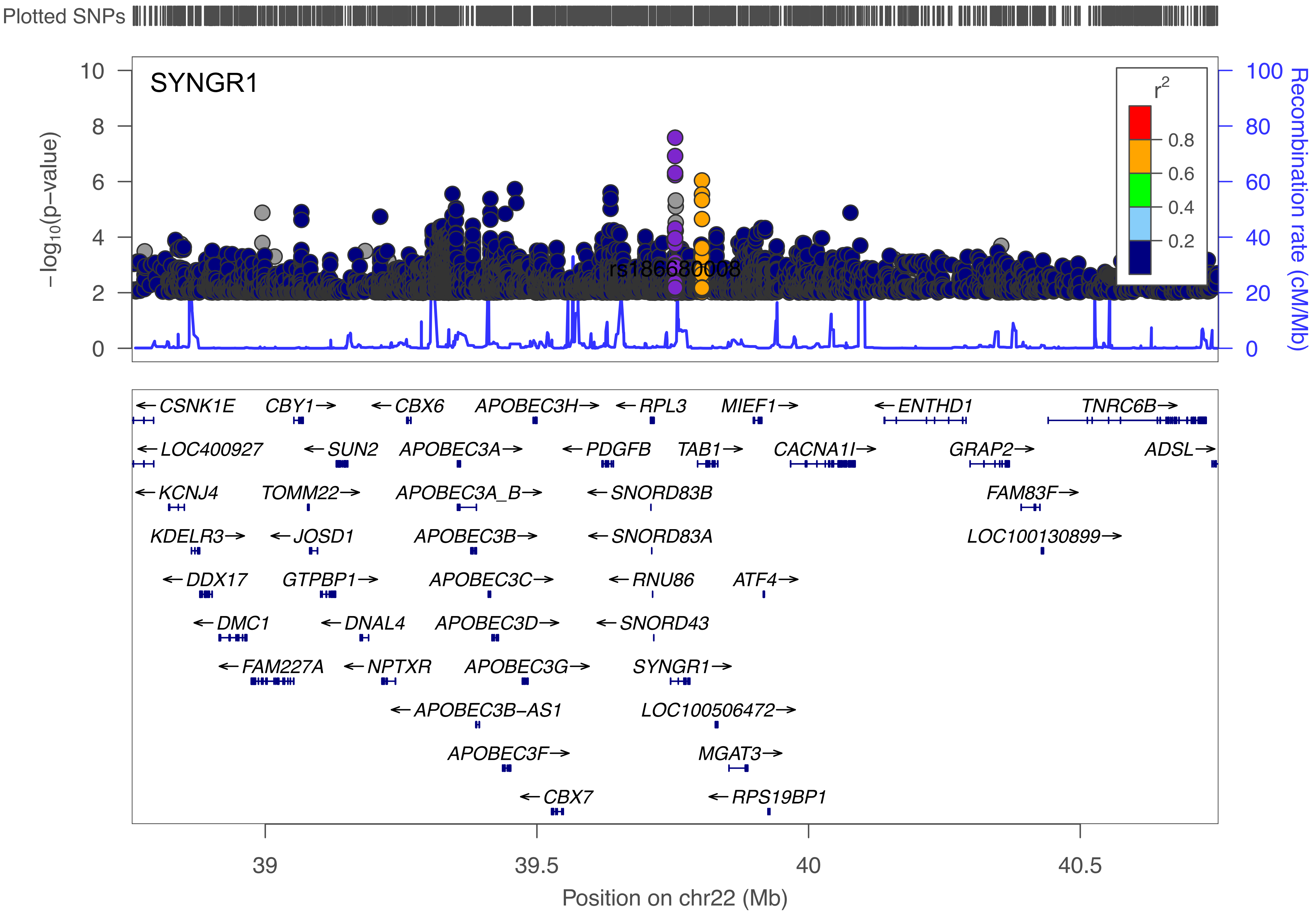

Plotted SNPs
SYNGR1
rs186680008
−log10(p−value)
Recombination rate (cM/Mb)
r2
0.8
0.6
0.4
0.2
10
8
6
4
2
0
100
80
60
40
20
0
←CSNK1E
CBY1→
←CBX6
APOBEC3H→
←RPL3
MIEF1→
←ENTHD1
TNRC6B→
←LOC400927
←SUN2
APOBEC3A→
←PDGFB
TAB1→
CACNA1I→
GRAP2→
ADSL→
←KCNJ4
TOMM22→
APOBEC3A_B→
←SNORD83B
FAM83F→
KDELR3→
←JOSD1
APOBEC3B→
←SNORD83A
LOC100130899→
←DDX17
GTPBP1→
APOBEC3C→
←RNU86
ATF4→
←DMC1
←DNAL4
APOBEC3D→
←SNORD43
←FAM227A
←NPTXR
APOBEC3G→
SYNGR1→
←APOBEC3B−AS1
LOC100506472→
APOBEC3F→
MGAT3→
←CBX7
←RPS19BP1
39
39.5
40
40.5
Position on chr22 (Mb)
